# Double Arylation of the Indole Side Chain of Tri- and Tetrapodal Tryptophan Derivatives Renders Highly Potent HIV-1 and EV-A71 Entry Inhibitors†

**DOI:** 10.1021/acs.jmedchem.1c00315

**Published:** 2021-07-07

**Authors:** Olaia Martí-Marí, Belén Martínez-Gualda, Sofía de la Puente-Secades, Alberto Mills, Ernesto Quesada, Rana Abdelnabi, Liang Sun, Arnaud Boonen, Sam Noppen, Johan Neyts, Dominique Schols, María-José Camarasa, Federico Gago, Ana San-Félix

**Affiliations:** ‡ Instituto de Química Médica (IQM-CSIC), c/ Juan de la Cierva 3, E-28006 Madrid, Spain; § Área de Farmacología, Departamento de Ciencias Biomédicas y Unidad Asociada IQM-UAH, Universidad de Alcalá, E-28805 Alcalá de Henares, Madrid, Spain; ∥ Laboratory of Virology and Chemotherapy, Department of Microbiology and Immunology, Rega Institute for Medical Research, University of Leuven, B-3000 Leuven, Belgium

## Abstract

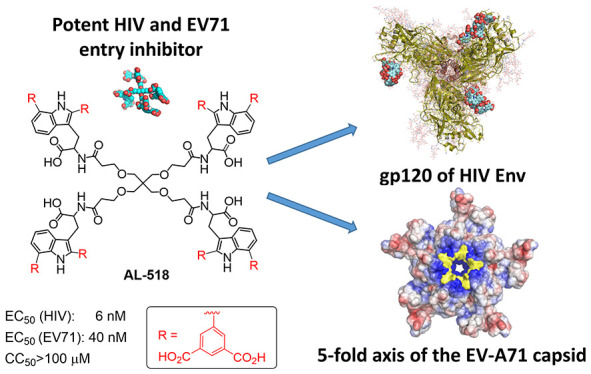

We have recently described a new generation of potent human immunodeficiency virus (HIV) and EV-A71 entry inhibitors. The prototypes contain three or four tryptophan (Trp) residues bearing an isophthalic acid moiety at the C2 position of each side-chain indole ring. This work is now extended by both shifting the position of the isophthalic acid to C7 and synthesizing doubly arylated C2/C7 derivatives. The most potent derivative (50% effective concentration (EC_50_) HIV-1, 6 nM; EC_50_ EV-A71, 40 nM), **33** (**AL-518**), is a C2/C7 doubly arylated tetrapodal compound. Its superior anti-HIV potency with respect to the previous C2-arylated prototype is in consonance with its higher affinity for the viral gp120. **33** (**AL-518**) showed comparable antiviral activities against X4 and R5 HIV-1 strains and seems to interact with the tip and base of the gp120 V3 loop. Taken together, these findings support the interest in **33** (**AL-518**) as a useful new prototype for anti-HIV/EV71 drug development.

## Introduction

The entry of human immunodeficiency virus (HIV) into host cells is a complex multistage process mediated by the viral envelope (Env) spike glycoproteins gp120 and gp41.^1−4^ Each of the entry steps is critical in the HIV life cycle and consequently represents an attractive target for the development of new antiviral agents.^5−10^ In fact, compounds that interfere with these early steps have several advantages over other existing therapeutic approaches that target intracellular viral enzymes such as reverse transcriptase or protease. First, they may prevent healthy cells from being infected with HIV and thereupon block the spread of infection. Second, entry inhibitors have the advantage of acting before the virus is inside the cell, thus eliminating the need to cross the cell membrane. Finally, because of their distinct mechanism of action, entry/fusion inhibitors are likely to show remarkable efficacy against viruses resistant to other classes of antiretroviral drugs (e.g., reverse transcriptase, protease, and integrase inhibitors).

At present, four entry inhibitors have been approved by the Food and Drug Administration (FDA) for the treatment of HIV infection: enfuvirtide (T20),^11^ which targets the envelope glycoprotein gp41; maraviroc,^12^ which targets the host cell chemokine receptor type 5 (CCR5); ibalizumab, a novel anti-CD4 monoclonal antibody (mAb),^13,14^ and fostemsavir (Rukobia), a prodrug of temsavir that has been very recently approved for the treatment of patients with HIV who cannot be treated with other therapies.^15^ This drug binds directly to gp120 and avoids the interaction between the virus and cellular CD4 receptors, thus preventing attachment.^16^

The Env glycoprotein gp120 is crucial for the HIV entry process because it serves as the first point of contact with the host cell’s CD4 receptor prior to binding to the chemokine coreceptors, CCR5 or CXCR4, and subsequent triggering of the membrane fusion event.^17,18^ The third variable loop (V3) of gp120 is particularly important because it has been implicated in many viral functions, including coreceptor binding and determination of cell tropism.^19^ In fact, positively charged residues are frequently found at critical positions in the V3 loop in CXCR4-tropic viruses^20^ while neutral or negatively charged residues are found at these positions in CCR5-tropic viruses.^21^ This loop is also a major antigenic determinant for inducing the formation of anti-HIV-1 antibodies.^22^ For this reason, the V3 loop is considered an important candidate epitope for anti-HIV-1 vaccine design purposes.

Many broadly neutralizing antibodies (bnAbs) isolated from HIV-1-infected individuals target epitopes that combine host-derived glycans with Env components.^23^ Although the glycans serve to block much of the polypeptide surface from antibody recognition, they frequently become a target of recognition themselves. One of these epitopes is the so-called “V3-glycan supersite”, an oligomannose patch around the V3 loop that is centered on the Asn332 residue.^24^ In this respect, structural studies have demonstrated that different bNAbs can adopt distinct binding orientations when targeting this supersite through specific interactions between residues in their complementarity-determining regions (CDR) and both polar and apolar faces of the sugar rings, which vary from complex to complex regarding density, clustering, and conformation of the constituent glycans.^25^

**Figure 1:**
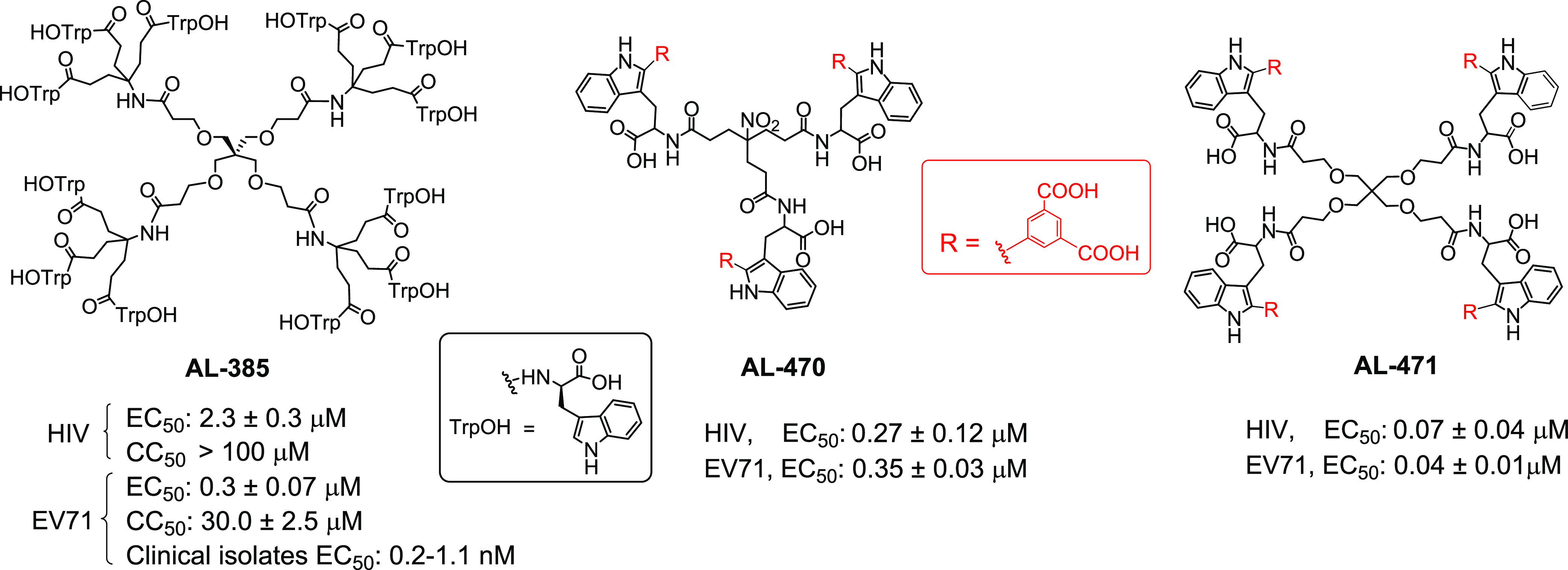
Structures of tetrapodal **AL-385** and reduced size prototypes (tripodal **AL-470** and tetrapodal **AL-471**).

These observations raise the prospect that similar intermolecular interactions, namely, ring stacking and hydrogen bonds, can be achieved by means of (i) flat surfaces such as those provided by indole and phenyl rings and (ii) hydrogen-bond acceptors such as carboxylic acids. Building on these premises, we have previously reported that a family of tri- and tetrapodal tryptophan (Trp) derivatives inhibits an early step in the infection of HIV by interacting with gp120 and preventing cell entry.^26^ The prototype is **AL-385**, which displays 12 Trp moieties attached to a tetrapodal central scaffold via their amino groups (Figure 1). Remarkably, these molecules also block entry of EV71,^27^ a completely unrelated neurotropic enterovirus that causes serious health problems in children under 5 years of age.^28−32^ In the last two decades, the increasing number of EV71 cases and the spread of the virus across Asia, followed by case reports in different European countries, raised major concerns about its pandemic potential.^33−35^ Because no antiviral therapies are so far available for the prevention or treatment of EV71 infection, the development of effective and specific antiviral drugs is urgently needed.^36,37^

Using a scaffold simplification strategy, we recently described a second family of Trp derivatives of reduced size and dual anti-HIV and anti-EV71 activity.^38^ Representative prototypes of this second family are the tripodal **AL-470** and tetrapodal **AL-471** derivatives, which contain either three or four Trp residues, respectively, each bearing an isophthalic acid moiety at the C2 position of the indole ring (Figure 1). By a surface plasmon resonance (SPR) assay, we have demonstrated that the anti-HIV potency of both compounds is related to their interaction with gp120.^38^ Moreover, cross-resistance experiments, followed by cryo-electron microscopy (cryo-EM) and computer-assisted modeling studies, revealed that **AL-471** interacts with the fivefold axis of the EV-A71 capsid, in particular with VP1 residues Lys244 (K244) and Tyr245 (Y245).^38^ Thus, binding of **AL-471** to this region prevents the interaction of the virus with its cellular (co)receptors P-selectin glycoprotein ligand-1 (PSGL-1) and heparan sulfate, thereby blocking the attachment of EV-A71 to the host cells. These results highlighted the promising potential of **AL-470** and **AL-471** as lead compounds for further development of novel HIV and EV71 entry inhibitors that could also be potentially active against other viruses that share common structural features in their capsids.

Preliminary structure–activity relationship (SAR) studies on the series encompassing **AL-470** and **AL-471** prototypes demonstrated that the presence of extra phenyl rings bearing one or two carboxylates at the C2 position of the indole ring of each Trp residue is critical for anti-HIV/EV71 activity. In stark contrast, this type of substitution at the N1 position of the indole moiety led to a substantial decrease of anti-HIV/EV71 efficacy. Incidentally, placing phenyl rings with substituents other than COOH at positions N1 or C2 led to inhibitors of dengue and Zika virus infection that also interfere with viral attachment processes.^39^

**Figure 2:**
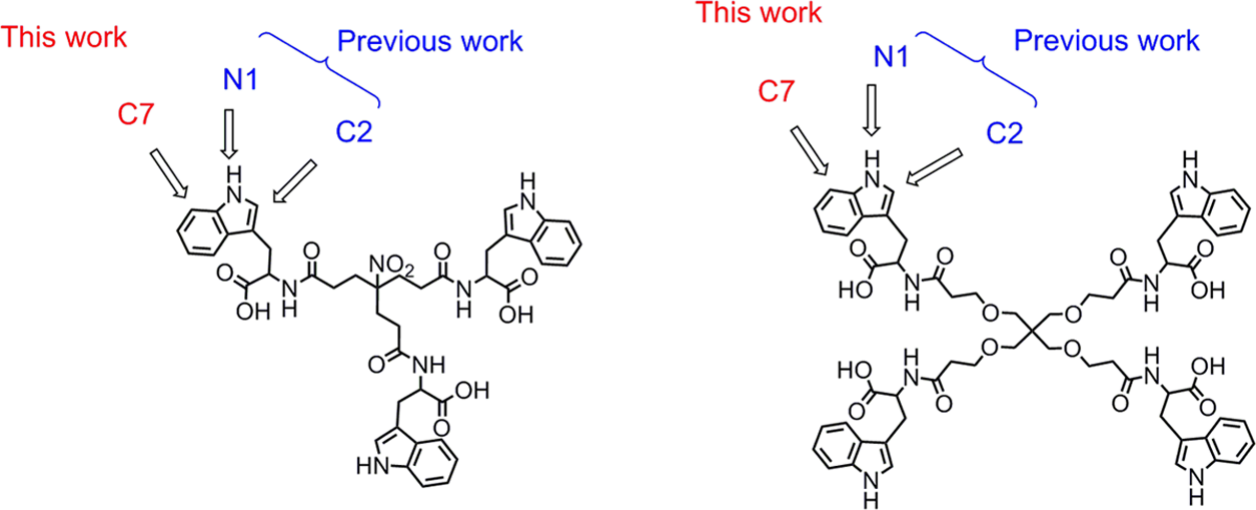
Indole positions explored for arylation in previous work (blue color) and in the present work (red color).

The global aim of the present work was to expand the previous SAR studies by exploring other positions of the side-chain indole of Trp, different from C2 or N1, for introducing the crucial carboxyl-containing phenyl rings. To this end, position C7 was chosen (Figure 2), and the impact of this novel structural modification on the anti-HIV and anti-EV71 activities was investigated.

## Results and Discussion

### Chemistry

**Scheme 1:**
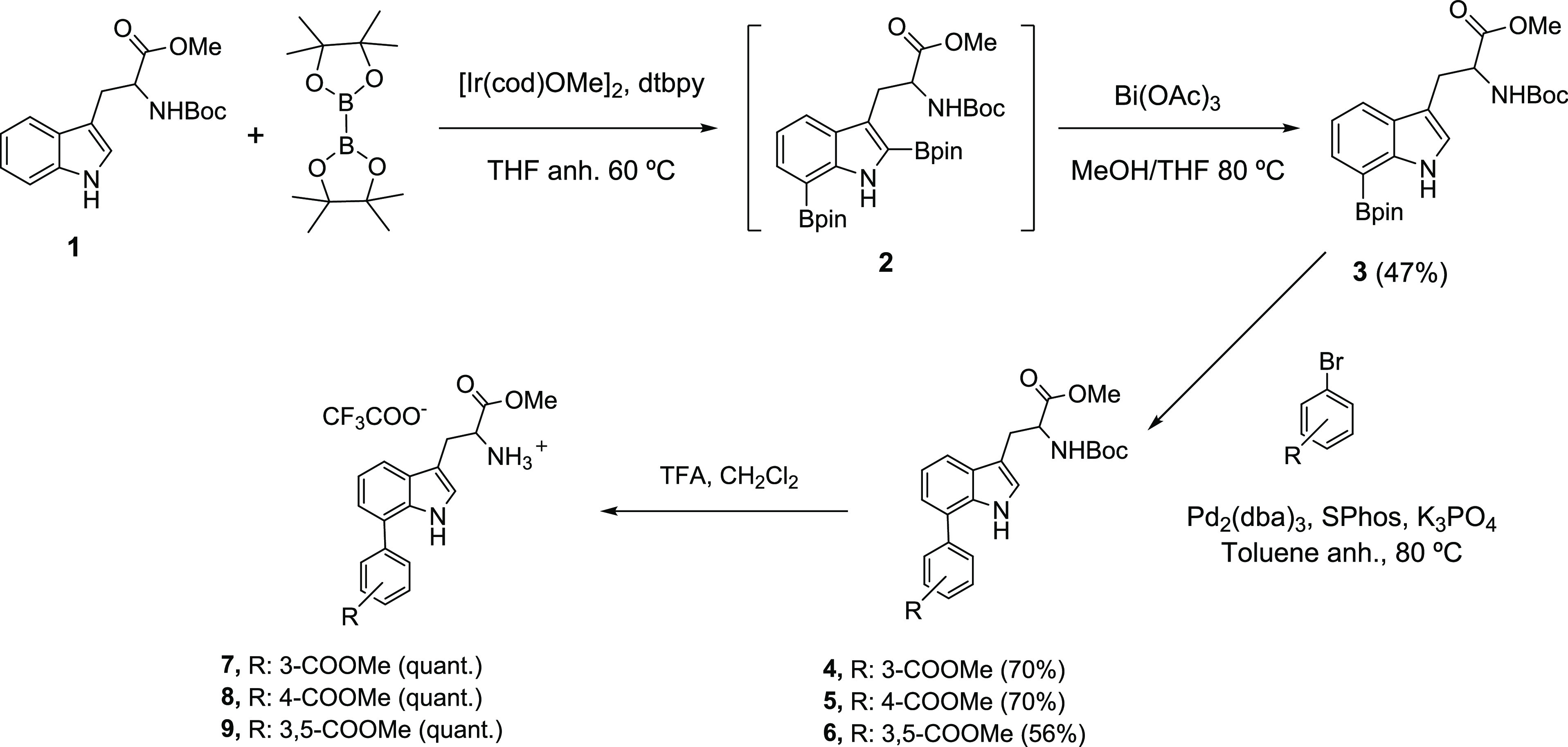
Synthesis of C7 Organoboron **3** and Arylated Trp Intermediates **7**–**9**

**Scheme 2:**
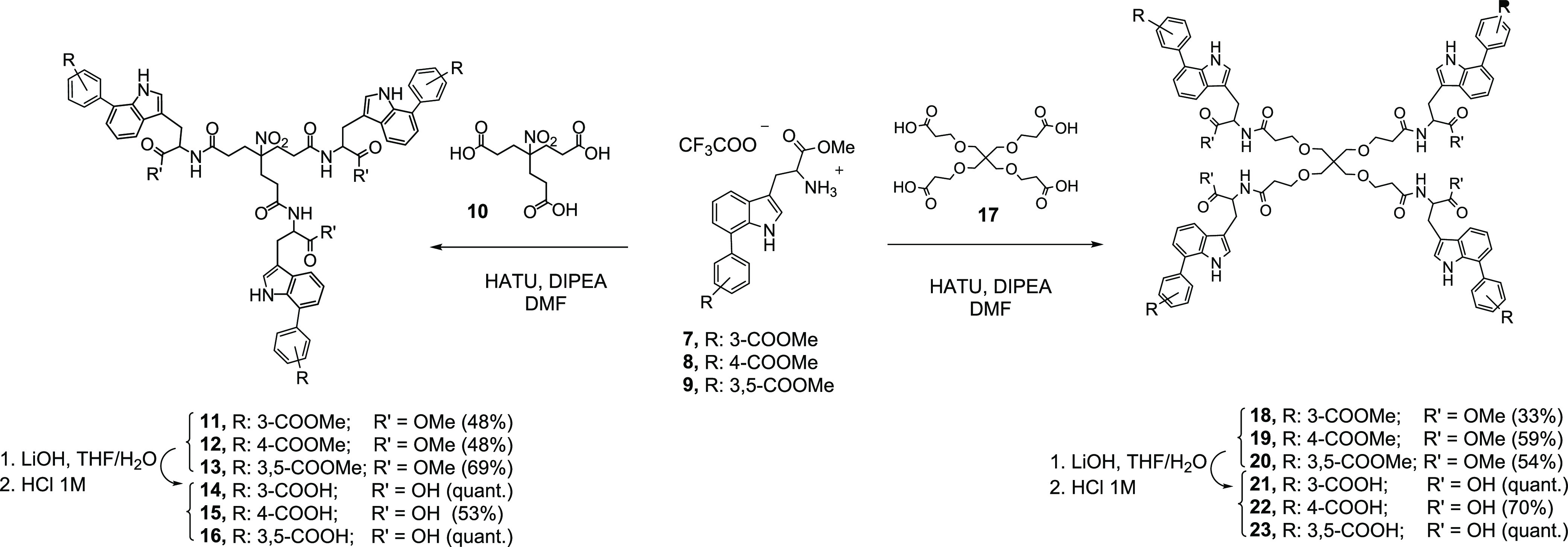
Synthesis of C7-Arylated Tripodal (**14**–**16**) and Tetrapodal (**21**–**23**) Derivatives

For the synthesis of the C7-arylated compounds, a novel chemical approach consisting of several sequential steps was followed (Schemes 1 and 2). First, the C7-arylated Trp derivatives **7**–**9**, with a free amino group, were obtained (Scheme 1). Thereafter, these key intermediates were linked to the corresponding central scaffold (Scheme 2). Selective C7 arylation of Trp to afford the C7-arylated Trp derivatives **7**–**9** proved to be particularly challenging due to the lesser reactivity of the C7 position compared to the inherently greater reactivity of the azole C2 position. Several methods, based on metal-catalyzed C–H boronation, have been envisaged to meet this end.^40^ Of particular relevance to this work was a two-step methodology developed by Movassaghi–Smith–Maleczka that involves the synthesis of the C7-boronated Trp derivative **3** (Scheme 1).^41,42^ This route started with the commercially available *N*-Boc-Trp methyl ester **1**. The first synthetic step consisted of diboronation of **1**, which proceeded smoothly (60 °C, 12 h) under iridium-catalyzed conditions to afford the C2/C7 diboronated intermediate **2**. In our hands, **2** was obtained more efficiently when bis(pinacolate)diboron (B_2_pin_2_)^43^ was used in place of pinacolborane (HBpin)^41^ (Scheme 1). The second step involved selective C2 protodeboronation of the crude product **2** in the presence of bismuth acetate as the catalyst to afford the 7-borylated Trp derivative **3** in 47% yield.^41^ Mild reaction conditions, under bismuth acetate catalysis, were crucial to avoid the formation of complex mixtures and simultaneous elimination of the *N*-Boc-protecting group that was observed when acetic acid was used in place of bismuth acetate.

Next, a Suzuki–Miyaura reaction (SMR)^44^ involving the coupling of organoboron Trp intermediate **3**(41) to the corresponding aryl bromide using (Pd_2_(dba)_3_) as the palladium catalyst and SPhos at 80 °C in the presence of potassium phosphate (K_3_PO_4_) as the base afforded C7 aryl intermediates **4**–**6** in good yield (56–70%) (Scheme 1). Aryl bromides bearing only one carboxylic acid substituent at the meta (*m*-COOH) or para position (*p*-COOH) or two carboxylic acid substituents at positions 3 and 5 on the aromatic ring were used because previous work had shown that this type of substituents renders the most potent compounds.^38^ Subsequent NHBoc deprotection afforded intermediates **7**–**9**, with a free amino group, in quantitative yields. Thereafter, coupling of these amino intermediates **7**–**9** to the tripodal (**10**)^45^ or tetrapodal (**17**)^46,47^ central scaffolds afforded tripodal **11**–**13** and tetrapodal **18**–**20** derivatives, respectively (Scheme 2). Finally, saponification of the methyl ester groups (LiOH/H_2_O) furnished the desired tripodal and tetrapodal end products **14**–**16** and **21**–**23**, respectively, in high yields (Scheme 2).

**Scheme 3:**
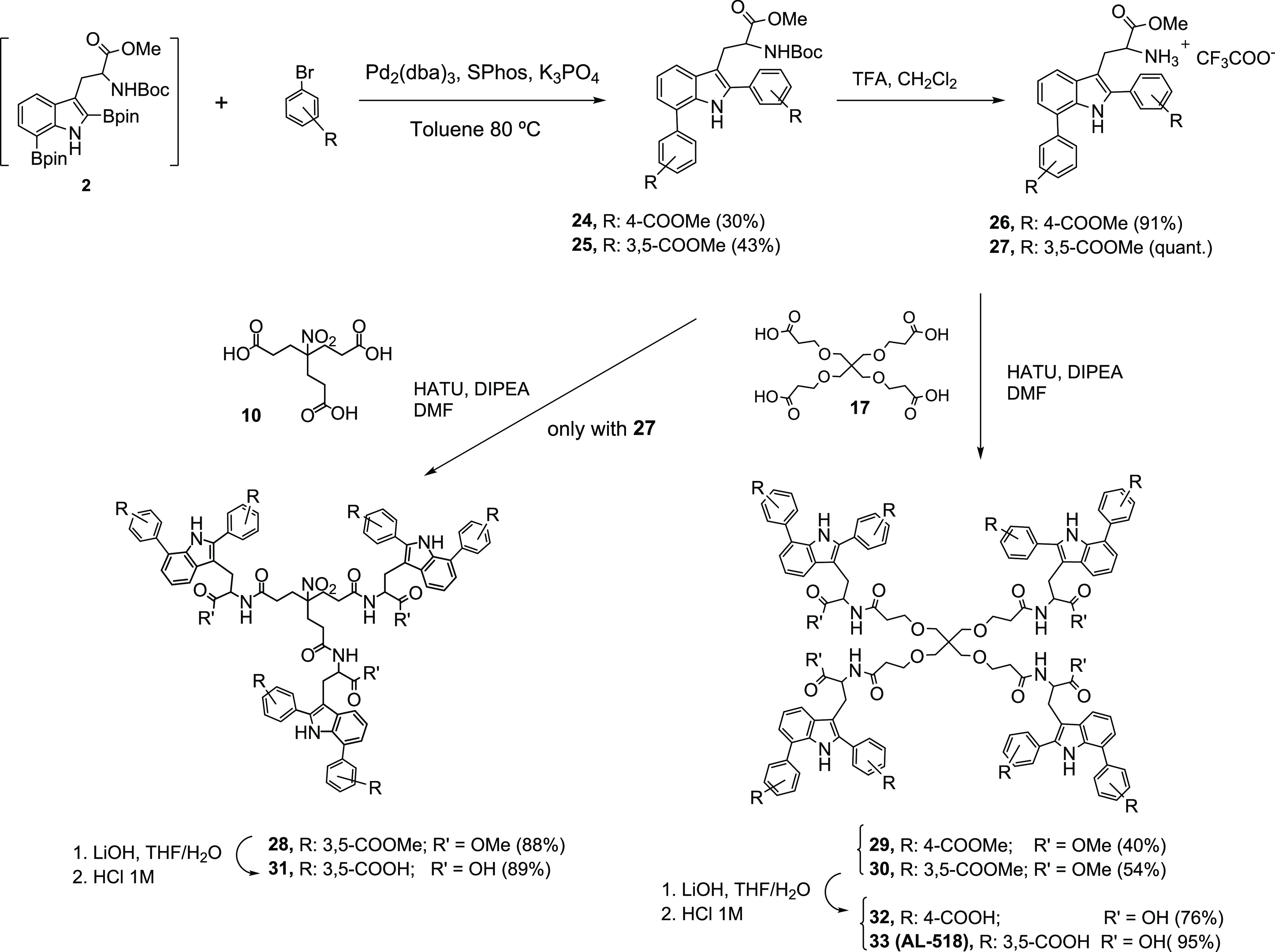
Synthesis of C2/C7-Diarylated Tripodal (**31**) and Tetrapodal (**32**, **33**) Derivatives

Doubly arylated Trp intermediates **24** and **25** were obtained through an SMR between the crude C2/C7 diboronated intermediate **2** and two different aryl bromides (Scheme 3). Subsequent removal of the Boc-protecting group and coupling of the resulting amino intermediates **26** and **27** to the tripodal (**10**)^45^ or tetrapodal (**17**)^46,47^ cores afforded **28** and **29**, **30**, respectively. Methyl ester saponification afforded the desired final C2/C7-arylated tripodal (**31**) and tetrapodal (**32**, **33**) derivatives in high yields.

**Scheme 4:**
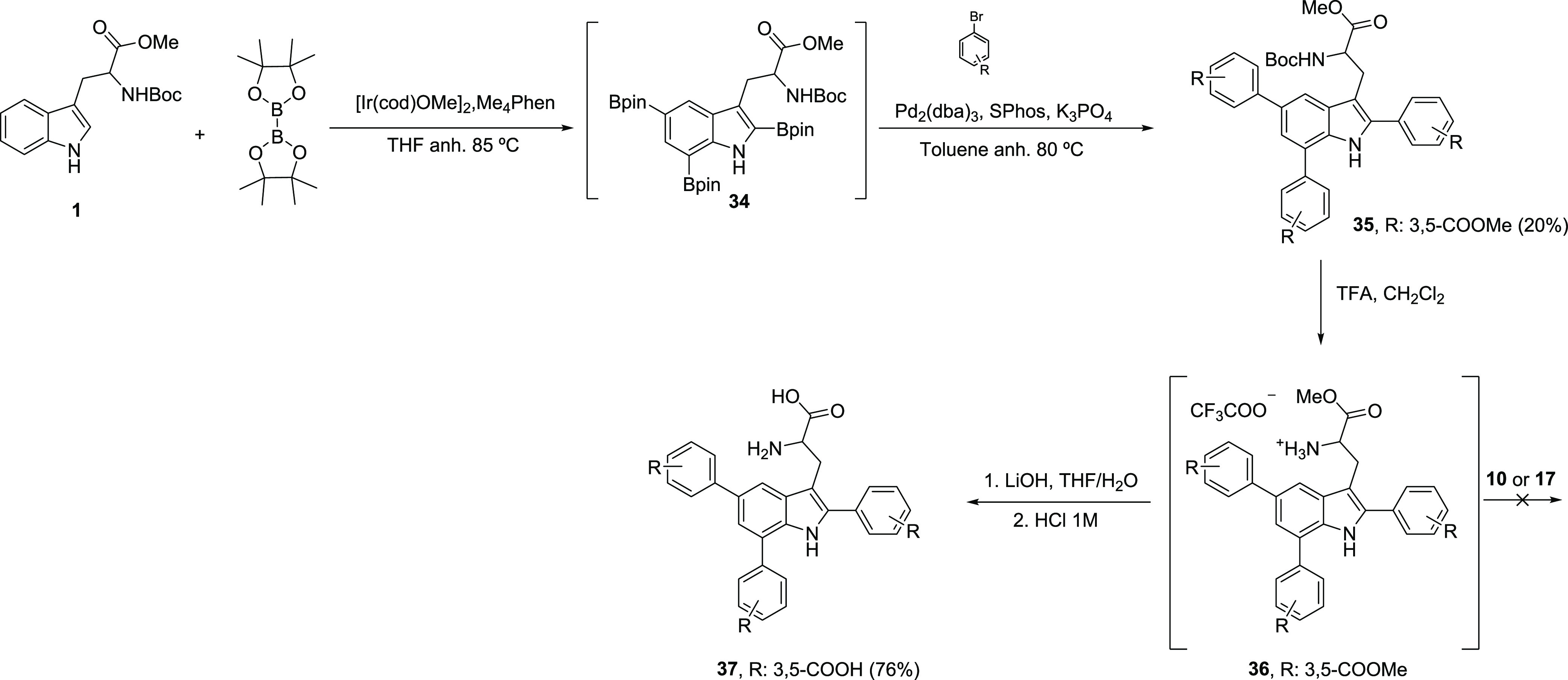
Synthesis of (C2/C5/C7)-Triarylated Trp Derivative **37**

To complete the SAR study, we decided to synthesize and test a triply arylated Trp derivative whose obtention would be accomplished through a convergent synthetic strategy similar to that described above (Scheme 4). The key intermediate in this route was triply arylated Trp derivative **35**, which was obtained through an SMR between the crude organoboron Trp intermediate **34**,^48^ bearing three Bpin substituents at the C2/C5/C7 positions, and isophthaloyl bromide. Subsequent removal of the Boc-protecting group afforded amino intermediate **36**. Coupling of this compound to the tripodal (**10**)^45^ or tetrapodal (**17**)^46,47^ central scaffolds was unsuccessful, probably due to steric hindrance. Instead, full methyl ester deprotection was performed to give functionalized Trp derivative **37**.

All of the final compounds had purities >95% based on high-performance liquid chromatography (HPLC), liquid chromatography/mass spectrometry (LC/MS), and ^1^H NMR analyses.

### Antiviral Evaluation

#### Activity against EV71

First, the C7-arylated tripodal (**14**–**16**) and tetrapodal (**21**–**23**) derivatives, together with their C2/C7 doubly arylated counterparts (**31**–**33**) and the C2/C5/C7 triply arylated Trp derivative **37**, were evaluated for their in vitro inhibitory activity against EV-A71 (Table 1). The previously described prototypes (**AL-470**, **AL-471**, and **AL-385**) (Figure 1), as well as pirodavir, an entry inhibitor that interacts with the viral capsid, were also assayed under the same conditions for comparative purposes.^49,50^ These tests were carried out in cell culture using rhabdomyosarcoma (RD) cells, which are known for their high susceptibility to EV71-induced cell death.^51^

**Table 1:** Antiviral Activity of the Synthesized Compounds against the BrCr Lab Strain of EV-A71 in RD Cells^a^

compound	EC_50_ (μM)^b^	CC_50_ (μM)^c^	SI^d^
**14**	54.8 ± 34.1	>84	1
**15**	61.6 ± 25.1	>61	1
**16**	1.2 ± 0.2	>75	63
**21**	3.1 ± 1.1	>61	20
**22**	1.7 ± 0.4	>100	60
**23**	0.3 ± 0.04	>55	157
**31**	0.2 ± 0.03	>100	526
**32**	0.1 ± 0.02	>100	667
**33** (**AL-518**)	0.04 ± 0.01	>100	2500
**37**	>90	ND^e^	ND
**AL-470**	0.3 ± 0.03	>75.3	203
**AL-471**	0.04 ± 0.01	>100	2500
**AL-385**	0.3 ± 0.1	24.9	83
pirodavir	0.3 ± 0.1	>100	333

a All average values are in micromolar (μM) and are a summary of multiple dose–response curves (>2) in multiple independent (>1) experiments.

b EC_50_: concentration of the compound at which the virus-induced cytopathic effect (CPE) is reduced by 50%.

c CC_50_: concentration of the compound at which a 50% reduction in cell viability is observed.

d SI: selectivity index (CC_50_/EC_50_).

e ND: not determined.

C2/C5/C7 triply arylated Trp derivative **37** did not show any anti-EV71 activity at concentrations up to 90 μM. The lack of activity of this molecule could be attributed to its small size (only one indole ring) or rigidity. This finding is consistent with our previous results suggesting an important role for multivalency in the eventual antiviral activity of this type of compound.^26,38^ Consequently, the presence of a central scaffold radiating three or four Trp units decorated with phenyl rings bearing carboxylic groups, particularly isophthalic acid moieties, is crucial for activity.

Moving the COOH aryl substituent from C2 to C7 resulted in less potent compounds, as can be seen by comparing the novel C7-arylated tripodal **16** (EC_50_: 1.2 μM) with C2-arylated prototype **AL-470** (EC_50_: 0.3 μM) and the novel C7-arylated tetrapodal **23** (EC_50_: 0.3 μM) with **AL-471** (EC_50_: 0.04 μM).

The derivatives possessing a tetrapodal skeleton [**21**–**23**, **32**, and **33** (**AL-518**)] are considerably more potent (EC_50_: 0.04–3.1 μM) than their counterparts with a tripodal skeleton **14**–**16**, **31** (EC_50_: 0.2–61.6 μM). Tetrapodal **21** (EC_50_: 3.1 μM) and tripodal **14** (EC_50_: 54.8 μM), substituted on C7 with phenyl rings carrying one COOH at the meta position, have a potency similar to that of their counterparts **22** (EC_50_: 1.7 μM) and **15** (EC_50_: 61.6 μM), with phenyl rings carrying one COOH at the para position. This data seems to indicate that the position of the carboxylic acid group on the phenyl ring does not have a great impact on anti-EV71 activity. Finally, **33** (**AL-518**), which is doubly arylated on C2 and C7 with isophthalic acid moieties, turned out to be as potent (EC_50_: 0.04 μM) as C2-arylated prototype **AL-471** (EC_50_: 0.04 μM). Taken together, the SAR data indicate that 3,5-COOH substitution on the phenyl ring is advantageous over single 3- or 4-substitutions (cf. **23** vs **21** and **22**, and **33** vs **32**) and also that C2/C7 arylation is superior to C7 arylation alone (cf. **33** vs **23**) but not to C2-arylation with isophthalic acid (cf. **33** vs **AL-471**).

Subsequently, compound **33** (**AL-518**), with the best activity/toxicity profile, was evaluated in cell-based assays against (i) a panel of other representative enteroviruses, (ii) a panel of clinical EV71 isolates representative of (sub)genogroups B2, B5, C2, and C4, and (iii) EV-A71 strains that harbor single (S184T and P246S) and double (S184T/P246S) mutations in VP1 that confer resistance to the **AL-385** prototype,^26,27^ which has been demonstrated to bind to the fivefold vertex of the viral capsid.^52^ The prototypes **AL-470** and **AL-471** were also included as reference compounds.

The summary of the results obtained is that **33** (**AL-518**) is (i) quite a specific inhibitor of EV71 replication because no antiviral activity was observed against the other viruses assayed (Table 2); (ii) active against all of the clinical strains (B2, B5, C2, and C4) (Table 3), but in contrast to prototypes **AL-470** and **AL-471**, it is less potent against B2 and C2 viruses than against the BrCr lab strain (sub-genogroup A)—although its activity on the clinical isolates B5 and C4 was improved with respect to that found for the BrCr lab strain albeit in a lesser degree than that found for the prototypes **AL-470** and **AL-471**; and (iii) more resilient to the resistance-conferring mutations because the loss of sensitivity against these strains was only two- to fourfold (Table 4). Considering the structural similarity between **33** (**AL-518**) and **AL-471**, we surmise that **33** (**AL-518**) also binds to VP1 residues around the fivefold axis but its binding affinity (or avidity) does not diminish significantly in the presence of these amino acid replacements. Our molecular modeling results (see below) offer a plausible explanation to these findings.

**Table 2:** Evaluation of Compound **33** (**AL-518**) against a Panel of Representative Enteroviruses

species	virus	host cell	EC_50_ (μM)^a^
enterovirus A	enterovirus A71 BrCr strain	RD	0.04 ± 0.007^b^
enterovirus B	coxsackievirus B3 Nancy strain	Vero A^c^	>50
enterovirus D	enterovirus D68 CU70 strain	HeLa Rh	>50
rhinovirus A	rhinovirus 2	HeLa Rh	>50
rhinovirus B	rhinovirus 14	HeLa Rh	>50

a All values were obtained from multiple (>2) independent (>1) experiments.

b Following microscopic quality control, at least at one concentration of compound, no virus-induced cell death was observed, and the compound did not cause an adverse effect on the host cell on monolayer morphology.

c Vero cells, African green monkey kidney cells.

**Table 3:** Evaluation of the Broad-Spectrum Antiviral Effect of **33** (**AL-518**) against a Representative Panel of EV71 Clinical Isolates in RD Cells

EV71 genogroup	virus strain	**33** (**AL-518**) EC_50_ (nM)^a^	**AL-47**0 EC_50_ (nM)^a^	**AL-471** EC_50_ (nM)^a^
A	BrCr	43 ± 6	353 ± 31	109 ± 34
B2	11316	101 ± 11	179 ± 11	19 ± 0.6
B5	TW/70902/08	10 ± 1.5	81 ± 13	5.9 ± 0.7
C2	H08300 461#812	120 ± 6	57 ± 0.2	2.6 ± 0.6
C4	TW/1956/05	11 ± 0.2	38 ± 3	1.4 ± 0.7

a All values are in nanomolar (nM) and were obtained in multiple (>2) independent (>1) experiments. Following microscopic quality control, at least at one concentration of compound, no virus-induced cell death was observed, and the compound did not cause an adverse effect on the host cell or monolayer morphology.

**Table 4:** Cross-Resistance Data and Resilience of **33** (**AL-518**) to Mutations in VP1

	EC_50_ (μM)^a^		
EV-A71	**AL-385**	**AL-471**	**33** (**AL-518**)
BrCr wild type	0.3 ± 0.01	0.1 ± 0.03	0.03 ± 0.01
VP1 (S184T)	2.0 ± 0.1 (*7.1*)	0.9 ± 0.05 (*8.5*)	0.1 ± 0.01 (*4.3*)
VP1 (P246S)	4.7 ± 0.2 (*16.8*)	1.9 ± 0.04 (*5.6*)	0.03 ± 0.01 (*1.0*)
VP1 (S184T/P246S)	8.9 ± 0.5 (*31.9*)	2.2 ± 0.08 (*6.2*)	0.06 ± 0.04 (*2.0*)

a Averages and standard deviations (SDs) were calculated from data obtained from three independent antiviral assays. Italicized numbers in parentheses indicate fold resistance.

#### Activity against HIV

**Figure 3:**
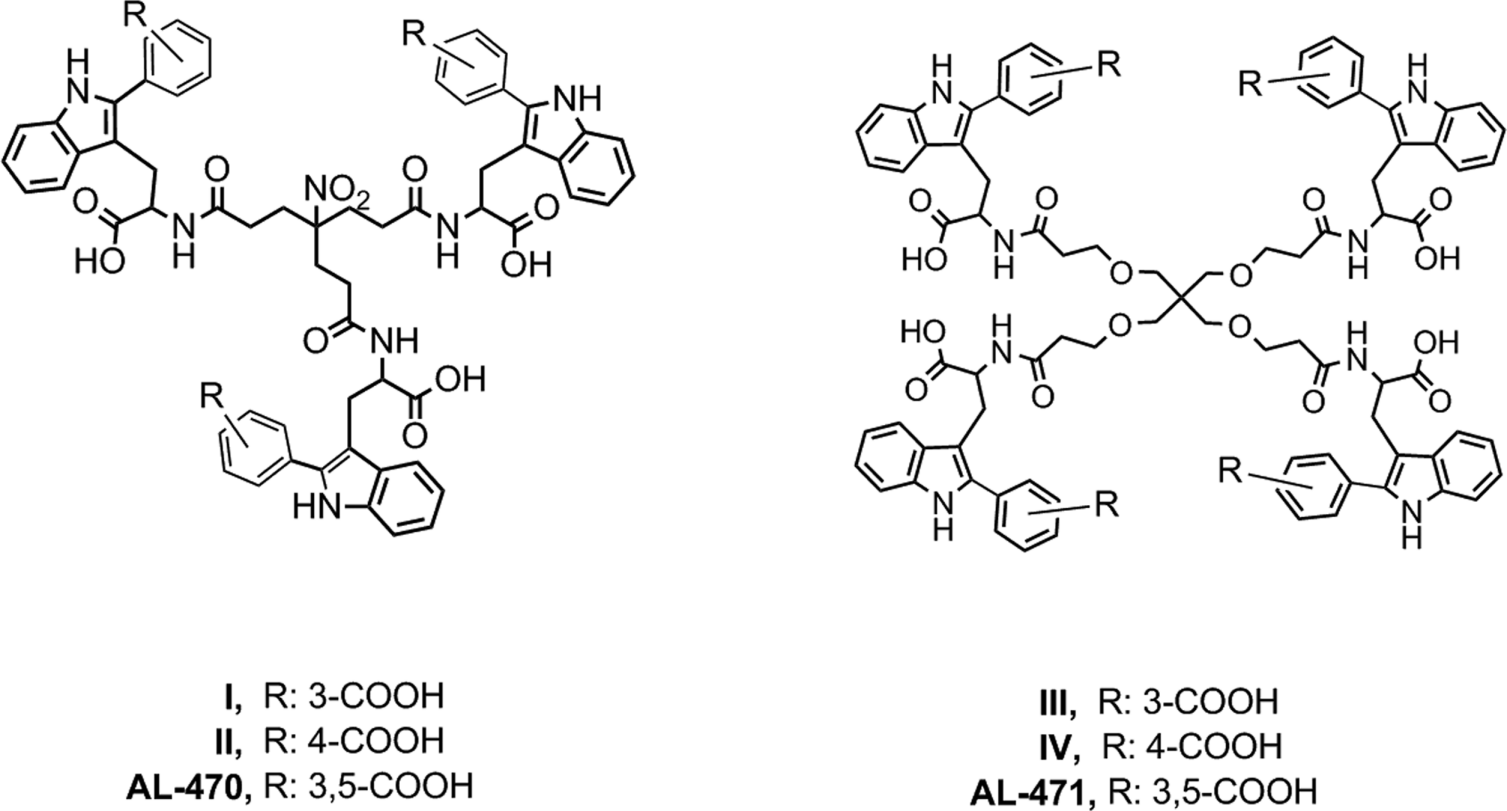
Structure of the previously reported C2-arylated Trp derivatives **I**–**IV** and prototypes **AL-470** and **AL-471**.

The newly synthesized compounds were also evaluated for their in vitro inhibitory activity against HIV-1 and HIV-2 replication in cultured CD4^+^ T cells (Table 5). The previously described C2-arylated tripodal (**I**, **II**) and tetrapodal (**III**, **IV**) derivatives (Figure 3), as well as prototypes **AL-470** and **AL-471** (Figure 1), were assayed under the same conditions for comparative purposes. The reference compounds dextran sulfate-5000 (DS-5000), a negatively charged HIV adsorption inhibitor,^53^ and pradimicin A (PRM-A), a gp120 carbohydrate-binding entry inhibitor,^54^ showed antiviral activities within previously reported ranges.^53,54^

**Table 5:** Anti-HIV-1 and HIV-2 Activities of the Synthesized Compounds against HIV-1 and HIV-2 in MT-4 Cells

compound	HIV-1 (NL4.3) EC_50_ (μM)^a^	HIV-2 (ROD) EC_50_ (μM)^a^	CC_50_ (μM)^b^	SI^c^
**14**	45.1 ± 10.2	>30	>100	24.4
**15**	12.0 ± 10.2	49.7 ± 8.6	>100	8.3
**16**	0.4 ± 0.2	1.9 ± 0.4	>100	232.6
**21**	14.1 ± 0.6	7.9 ± 0.9	>100	7.1
**22**	6.5 ± 2.5	0.4 ± 0.3	>100	15.5
**23**	0.02 ± 0.01	0.02 ± 0.02	75.00	4687.5
**31**	0.04 ± 0.01	0.07 ± 0.01	>100	2564.1
**32**	0.2 ± 0.2	0.02 ± 0.02	>100	476.2
**33** (**AL-518**)	0.006 ± 0.005	0.06 ± 0.02	>100	16 129
**37**	>100	>100	>100	
**I**	16.0 ± 5.7	64 ± 28	>100	6
**II**	2.4 ± 1.3	>71	71	29.6
**III**	2.0 ± 0.6	0.8 ± 0.5	>100	50
**IV**	0.7 ± 0.5	0.7 ± 0.8	>100	137
**AL-470**	0.3 ± 0.1	0.6 ± 0.8	>100	370
**AL-471**	0.07 ± 0.04	0.02 ± 0.09	>100	1429
DS-5000	0.07 ± 0.02	0.03 ± 0.01	>20	285.7
PRM-A	3.3 ± 1.2	5.9 ± 3.7	>100	30.3

a EC_50_: concentration of the compound at which the virus-induced cytopathic effect is reduced by 50%.

b CC_50_: concentration of the compound at which a 50% reduction in cell viability is observed.

c SI: selectivity index (CC_50_/EC_50_).

The C2/C5/C7 triply arylated Trp derivative **37** did not show any anti-HIV activity at concentrations up to 100 μM (Table 5). This finding demonstrates, as reported above for EV71, the importance of multivalency for activity. For the multivalent derivatives, moving the COOH aryl substituent from C2 to C7 resulted in decreased anti-HIV potency, as can be seen by pairwise comparison of the range of activities displayed by the novel C7-arylated tripodal **14**–**16** (EC_50_: 45.1–0.4 μM) and tetrapodal **21** and **22** (EC_50_: 14.1–6.5 μM) derivatives versus those of the C2-arylated tripodal **I**, **II**, and **AL-470** (EC_50_: 16.0–0.3 μM) and tetrapodal **III** and **IV** (EC_50_: 2.0–0.7 μM) derivatives (Table 5). An exception was the C7-arylated tetrapodal compound **23** (EC_50_: 0.02 μM), which turned out to be 3.5-fold more potent than the respective C2 counterpart **AL-471** (EC_50_: 0.07 μM).

In contrast to what was observed for EV71, the compounds with the COOH aryl substituent at the para position were more potent than those with the COOH aryl substituent at meta, as can be seen by comparing tripodal **14** (EC_50_: 45.1 μM) and **15** (EC_50_: 12.0 μM), on the one hand, and tetrapodal **21** (EC_50_: 14.1 μM) and **22** (EC_50_: 6.5 μM), on the other.

Interestingly, tripodal **16** (EC_50_: 0.4 μM) and tetrapodal **23** (EC_50_: 0.02 μM) derivatives, both arylated at C7 of the indole ring with isophthalic acid, were the most potent of the C7 series. This result corroborates the previous finding that this moiety is so far the optimal aryl substituent for attachment to the Trp indole ring.^38^

Potency enhancement upon double arylation at C2 and C7 was observed in all members of the tripodal and tetrapodal series, as can be seen by comparing the activities shown by the doubly arylated **31** (EC_50_: 0.04 μM) with those of the C7-arylated **16** (EC_50_: 0.4 μM) and C2-arylated **AL-470** (EC_50_: 0.3 μM) and also those of the doubly arylated **32** (EC_50_: 0.2 μM) with those of the C7-arylated **22** (EC_50_: 6.5 μM) and C2-arylated **IV** (EC_50_: 0.7 μM). Finally, double arylation at C2 and C7 with isophthalic acid moieties led to **33** (**AL-518**) (EC_50_: 0.006 μM), which demonstrated significantly improved anti-HIV activity over both the C2-arylated prototype **AL-471** (EC_50_: 0.07 μM) and the C7-arylated **23** (EC_50_: 0.02 μM). Interestingly, the anti-HIV-1 activity displayed by **33** (**AL-518**) was found to be far below its toxicity threshold (CC_50_ > 100 μM), and for this reason, the selectivity index (CC_50_/EC_50_ ratio, SI = 16 129) was greatly improved with respect to **AL-471** (SI = 1429). Moreover, **33** (**AL-518**) also demonstrated potent in vitro antiviral activity against HIV-2 (EC_50_ ≈ 0.06 μM).

Taken together, these data indicate that, of all of the Trp derivatives synthesized to date, the most potent against HIV (EC_50_: 6 nM) is **33** (**AL-518**), which can thus be considered a novel and promising lead compound.

### Mode of Action in the Context of HIV Infection

#### Surface Plasmon Resonance Analysis

As mentioned above, the prototype compounds **AL-470** and **AL-471** inhibit an early step in the replicative cycle of HIV by interacting with gp120 of the HIV envelope.^26,38^ To investigate whether this glycoprotein is also the potential target of the novel compounds, recombinant HIV-1 IIIB gp120 (produced in Chinese hamster ovary cells) was bound to a sensor chip as a monomer and its interaction with **33** (**AL-518**) was measured using SPR. The prototype **AL-471** was used as a positive control. The results confirm that compound **33** (**AL-518**), similarly to **AL-471**, binds to gp120 and the interaction is concentration-dependent.

**Figure 4:**
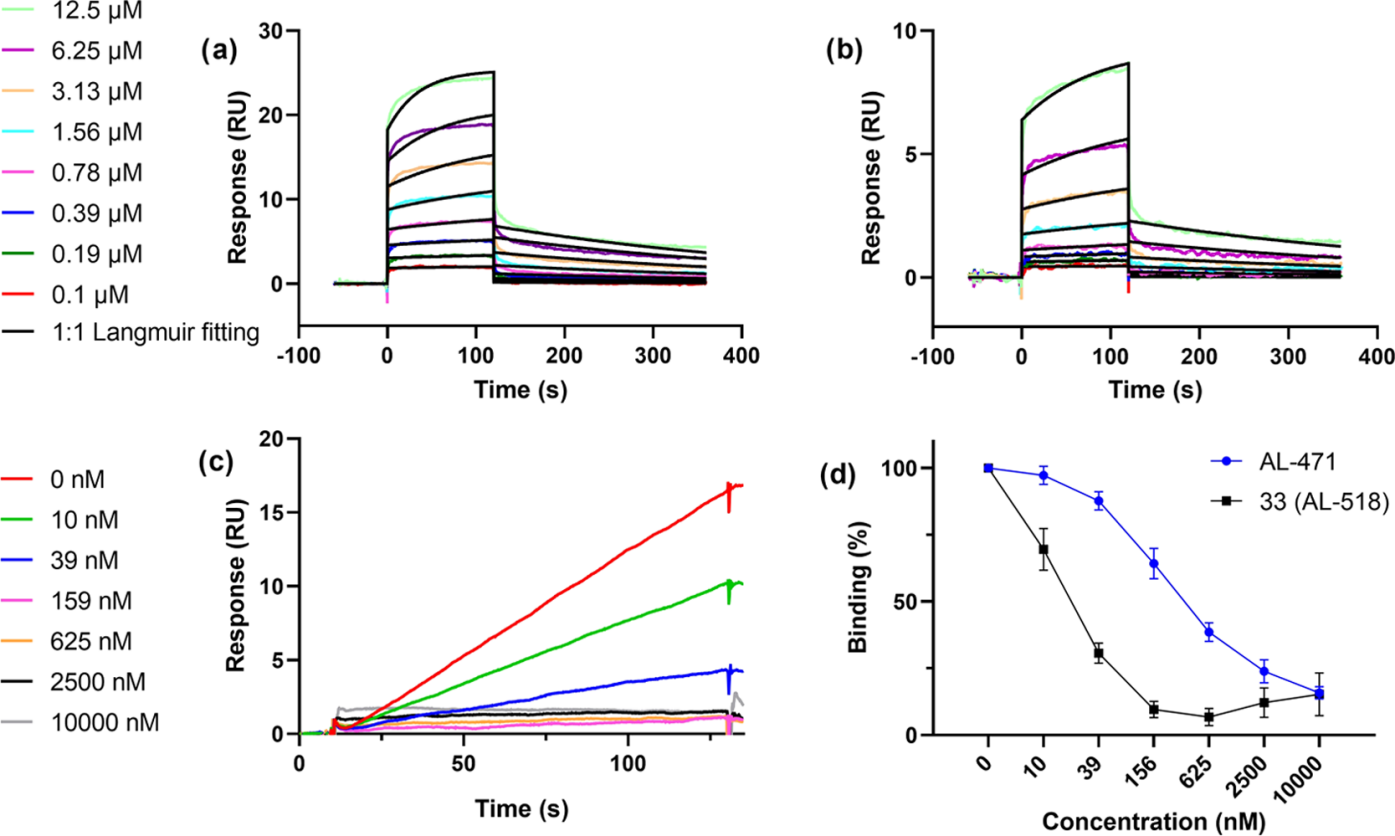
Multiple cycle kinetics of (a) **33** (**AL-518**) and (b) **AL-471** binding to HIV gp120 (results from one of four replicate experiments). Note the different scale of the *Y*-axes in left and right panels. The colored curves represent the real-time binding responses, whereas the black curves were obtained by fitting to a 1:1 Langmuir model. The concentrations ranged from 0.1 to 12.5 μM, and both analytes were diluted using twofold dilution steps. For both analytes, the ability to bind to gp120 was confirmed. (c) SPR sensorgram of **33** (**AL-518**) at varying concentrations mixed with 25 nM gp120 binding to immobilized CD4. Serial concentrations ranged from 10 000 to 10 nM using fourfold dilution steps. Results are shown from one of three replicate experiments. (d) Dose-dependent inhibition by **AL-471** and **33** (**AL-518**) of the binding of gp120 to CD4, calculated by the responses of three replicate SPR experiments.

Next, detailed SPR-directed affinity was determined using concentrations of **33** (**AL-518**) ranging from 12.5 to 0.1 μM (Figure 4a,b and Table 6). Tetrapodal **33** (**AL-518**) showed a higher association rate constant (*k*_a_) than did **AL-471**, which means that the former binds faster to gp120, and also a lower dissociation rate constant (*k*_d_), which entails a slower detachment from gp120. These kinetic results indicate that the interaction of gp120 with **33** (**AL-518**) (*K*_D_ = 0.84 μM) is around sixfold stronger than with **AL-471** (*K*_D_ = 5.31 μM).

**Table 6:** Apparent *K*_D_ for the Interaction between HIV-1 gp120 and **33** (**AL-518**)^a^

compound	*k*_a_ (×10^3^ M^–1^ s^–1^)	*k*_d_ (×10^–3^ s^–1^)	*K*_D_ (μM)
**33** (**AL-518**)	3.34 ± 1.02	2.45 ± 0.34	0.84 ± 0.34
**AL-471**	1.20 ± 0.55	6.97 ± 4.59	5.31 ± 1.89

a Average and standard deviation of kinetic parameters obtained from four replicate SPR experiments measuring the binding response between gp120 and **33** (**AL-518)**/**AL-471**.

Overall, the higher affinity (i.e., smaller *K*_D_) for gp120 of **33** (**AL-518**) than that of the prototype **AL-471** nicely correlates with the superior anti-HIV-1 activity that was observed for the former compound in the cell-based antiviral assay.

Direct inhibition of viral gp120 binding to the human cell receptor CD4 by **33** (**AL-518**) and **AL-471** was demonstrated by SPR. A dose-dependent decrease of binding was observed when 25 nM gp120 was mixed with varying concentrations of **33** (**AL-518**) (Figure 4c). These results, together with those of **AL-471** were plotted on a graph to investigate the differences in potency between the two compounds (Figure 4d). **33** (**AL-518**) showed a stronger inhibition profile compared to **AL-471**. Indeed, **33** (**AL-518**) inhibited 50% of gp120-CD4 binding in the 10–40 nM range, whereas 156–625 nM was needed to obtain the same degree of inhibition with **AL-471**. This means that **33** (**AL-518**) is at least 16 times more potent than **AL-471**.

#### Activity against CXCR4- and CCR5-Tropic HIV-1 Strains

We next investigated whether the action of **33** (**AL-518**) is dependent on the coreceptor used by HIV-1 for entry. To this end, the compound was tested against CXCR4- (HIV-1/NL4.3) and CCR5-tropic (HIV-1/Ba-L) HIV-1 strains in peripheral blood mononuclear cells (PBMCs). The reference compounds maraviroc, an HIV-1 entry inhibitor that works as a negative allosteric modulator of the chemokine receptor type 5 (CCR5),^12^ and bicyclam AMD3100 (aka plerixafor), which blocks HIV-1 entry and membrane fusion by acting as a partial agonist of the C-X-C α-chemokine receptor type 4 (CXCR4),^55^ showed antiviral activities within previously reported ranges.^12,55^

As shown in Table 7, **33** (**AL-518**) inhibited the replication of both CXCR4- and CCR5-tropic HIV-1 strains with similar potencies (EC_50_: 33 and 36 nM, respectively). In contrast, the CXCR4-tropic HIV-1 strain (NL4.3) was insensitive to the action of maraviroc, as expected, while the CCR5-tropic HIV-1 strain (HIV-1/Ba-L) was unaffected by AMD3100 at concentrations up to 1000 ng/mL. Thus, it seems that the action of **33** (**AL-518**) is independent of the coreceptor used by HIV-1 for entry. The prototype **AL-471** also inhibits both CXCR4- and CCR5-tropic HIV-1 strains, although it seems to be tenfold more potent against the CXCR4-tropic HIV-1 strain (NL4.3).

**Table 7:** Activities of **AL-471** and **33** (**AL-518**) against CXCR4- and CCR5-Tropic HIV-1 Strains^a^

compound	concentration units	HIV-1 (NL4.3) EC_50_^b^	HIV-1 (Ba-L) EC_50_^b^
**AL-471**	nM	76	280
**33** (**AL-518**)	nM	33	36
maraviroc	nM	>1000	4.48
AMD3100	ng/mL	3.57	>1000

a These values were obtained from four independent experiments.

b EC_50_: 50% inhibitory concentration or compound concentration required to inhibit the HIV-induced cytopathogenic effect in PBMC cells.

As mentioned in the Introduction section, positively charged residues are frequently found at critical positions in the V3 loop of CXCR4-tropic viruses,^20^ while neutral or negatively charged residues are found at these positions in CCR5-tropic viruses.^21^ The finding that **33** (**AL-518**) inhibits the productive infection of cells by both CXCR4- and CCR5-tropic HIV-1 strains is strongly suggestive of its binding to certain highly conserved structural features, e.g., *N*-glycans, even though the involvement of specific gp120 amino acids cannot be excluded.

#### Competition-Binding Experiments with Monoclonal Antibodies Directed to Specific Regions of the HIV-1 gp120 Envelope

We then investigated the putative gp120 binding site for this class of compounds using competition-binding experiments with mAbs directed to different protein regions: (i) human mAb clone 2G12, whose epitope, composed entirely of N-linked glycans, is centered at position N332;^56^ (ii) human mAb clone 447-52D, which recognizes the tip of the V3 loop;^57,58^ and (iii) the mouse clones 9284 and 9305, which interact with the base of the V3 loop (amino-terminal side).^59^

These tests were carried out in cell culture using CD4^+^ MT-4 T cells that express high levels of gp120 on their membrane when they become infected. Compound **33** (**AL-518**) was added and the staining results in the presence of the abovementioned antibodies were analyzed by flow cytometry. The prototype **AL-471** and the mannose-specific lectin *Hippeastrum* hybrid agglutinin (HHA)^60^ were also assayed under the same conditions for comparative purposes. The results of these evaluations are summarized in Table 8, as well as in Tables S1 and S2 (Supporting Information). It can be seen that the interaction of all of these mAbs with gp120 was unaffected by **AL-471** at concentrations up to 100 μM. In contrast, **33** (**AL-518**) caused dose-dependent inhibition of the binding of mAbs 447-52D and 9284 (average IC_50_ values of 6.8 and 3.8 μM, respectively). These results strongly suggest that **33** (**AL-518**) can interact with the same epitopes that are recognized by these mAbs, which implies some degree of overlap of their binding regions. The core epitope of mAb 447-52D is the highly conserved motif Gly–Pro–Gly–Arg/Gln (GPGR/Q, residues 312–315) at the tip of the V3 loop,^58,61^ while antibody 9284 interacts with the base of this same loop.^59^ Interestingly, **33** (**AL-518**) did not prevent the binding of the mAb 2G12, whose binding—as well as that of mAb 9284—was significantly affected by HHA.

**Table 8:** Inhibition Profile of **AL-471** and **33** (**AL-518**) on HIV-1-Envelope Directed Anti-gp120 mAbs^a^

compound	concentration units	2G12 IC_50_	447-52D IC_50_	9284 IC_50_	9305 IC_50_
**AL-471**	μM	>100	>100	>100	>100
**33** (**AL-518**)	μM	>100	6.8	3.8	>100
**HHA**	μg/mL	2.0	ND	2.1	>20

a MT-4 cells were infected with HIV-1 NL4.3 and 4 days after infection stained with or without the addition of the compounds in the presence of the selected set of specific anti-gp120 mAbs. Percentage inhibition was calculated on the mean fluorescence intensity by flow cytometry.

#### Putative Binding Modes Using Computational Analysis

**Figure 5:**
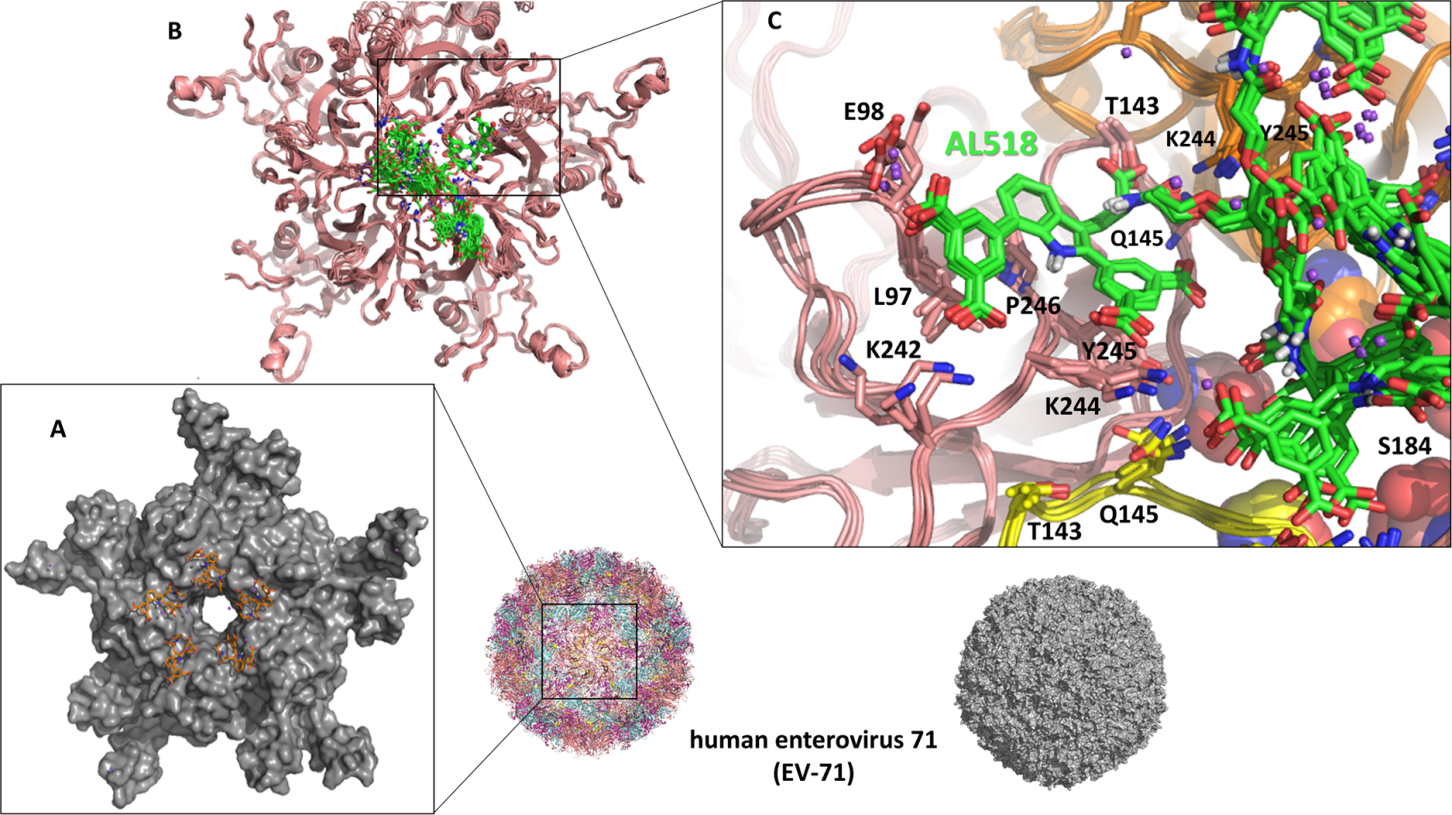
Molecular models of EV71. (A) Proposed binding mode of the decorated Trp moiety of **33** (**AL-518**) (stick representation, C atoms in orange) at the interface between subunits of the experimentally determined structure (PDB entry 6UH7(38)) of the VP1 pentamer (gray envelope). (B) Cartoon representation of VP1 (pink) in complex with **33** (**AL-518**) (sticks, with C atoms in green), as found along the molecular dynamics simulations. The ten superimposed structures represent a conformational ensemble made up of snapshots extracted from the molecular dynamics trajectory every 5 ns and then cooled down to 273 K and energy-minimized. (C) Detail of the proposed binding mode for **33** (**AL-518**) showing the extended aromatic surface and the functional groups responsible for specific interactions with protein residues, the most important of which have been labeled. The small spheres in violet represent sodium ions. Note the spatial positions of Ser184 and Pro246.

For attachment of **33** (**AL-518**) to EV-A71, we propose a binding mode similar to that reported for **AL-385**,^38^ consisting of the location of a single molecular entity at each capsid vertex where at least one of the decorated Trp moieties can establish multiple specific interactions with selected residues, while the rest of the molecule engages in additional contacts with neighboring amino acids (Figure 5). Thus, interaction of the virus with its cellular receptors PSGL-1 and heparan sulfate on the host cell membrane is prevented and EV-A71 entry is blocked.

**Figure 6:**
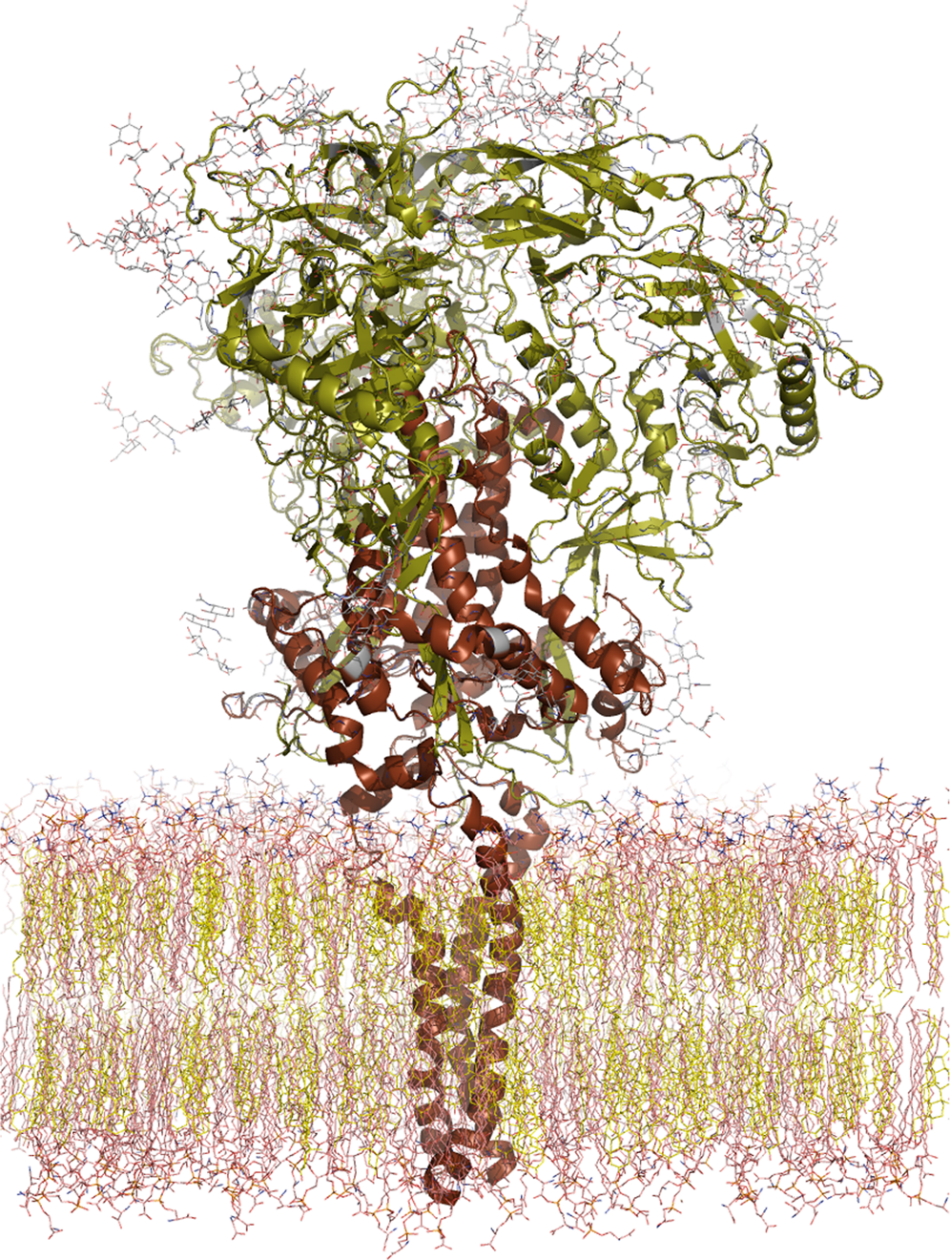
Molecular model of HIV-1 Env (cartoon representation), a trimer of gp120 (green) and gp41 (brown) dimers, embedded in a cholesterol-rich lipid bilayer (phospholipid and cholesterol C atoms colored in pink and yellow, respectively). Water molecules and counterions on both sides of the membrane have been omitted for clarity. The oligosaccharide residues GlcNAc_2_, Man_1_GlcNAc_2_, Man_3_GlcNAc_2_, and Man_9_GlcNAc_2_ attached to several asparagine side chains in both gp120 and gp41 are displayed as sticks, with C atoms in gray. These sugars represent only a fraction of the N-linked “high-mannose patches” that shield the HIV-1 envelope from immune surveillance^64^ but at the same time provide specific recognition by lectins, bNAbs, and, possibly, tetrapodal Trp derivatives such as **33** (**AL-518**).

In the case of HIV-1, we must bear in mind that the gp120 and gp41 subunits that make up the Env protein of the different HIV-1 subtypes or clades exist as populations of glycosylated variants (glycoforms) that are characterized by heterogeneous patches of oligomannose-type glycans at each glycosylation site whose composition is dependent on trimerization and trimming by mannosidases.^62^ Since we postulated that the decorated Trp residues in our series of derivatives provide not only a flat surface for CH···π interactions^63^ with the pyranose rings of the numerous asparagine-linked oligomannose glycans but also multiple hydrogen bonding possibilities and ionic interactions via their free carboxylic acid groups, we tested this hypothesis by first modeling and simulating the dynamic behavior of **33** (**AL-518**) in the presence of the high-mannose glycan Man_9_GlcNAc_2_-OMe. Thereafter, we modeled and simulated a membrane-embedded and heavily glycosylated HIV-1 Env trimer (Figure 6) to assess the extent of protein surface coverage as well as the extracellular region in the presence of three separate **33** (**AL-518**) molecules initially placed at the CD4-binding site in three different orientations. By using this procedure, our sampling of ligand–receptor interactions was effectively run in triplicate and the feasibility of intermonomer bridging by the four flexible arms radiating from the central scaffold could be assessed.

**Figure 7:**
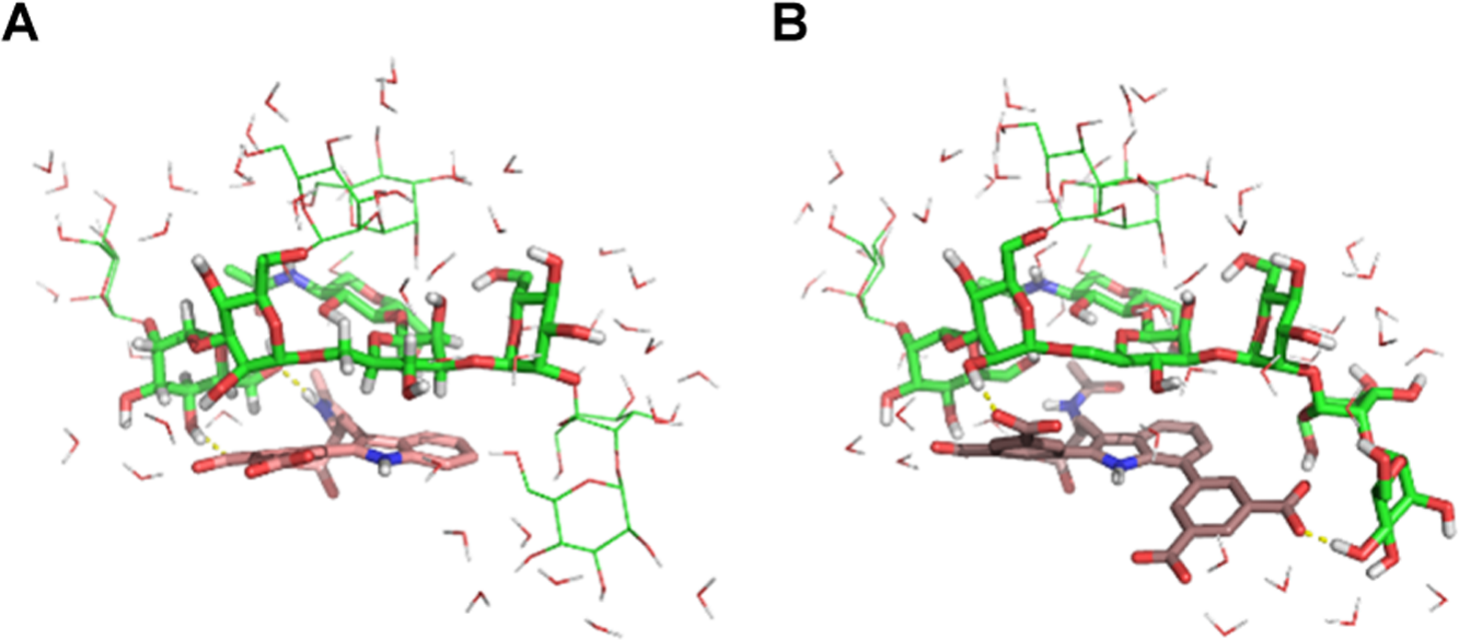
Simultaneous stacking of the central mannose in Man_9_GlcNAc on either a C2 singly arylated (A) or a C2/C7 doubly arylated (B) N-capped Trp residue and distinct hydrogen-bonding interactions with surrounding hydroxyl groups.

**Figure 8:**
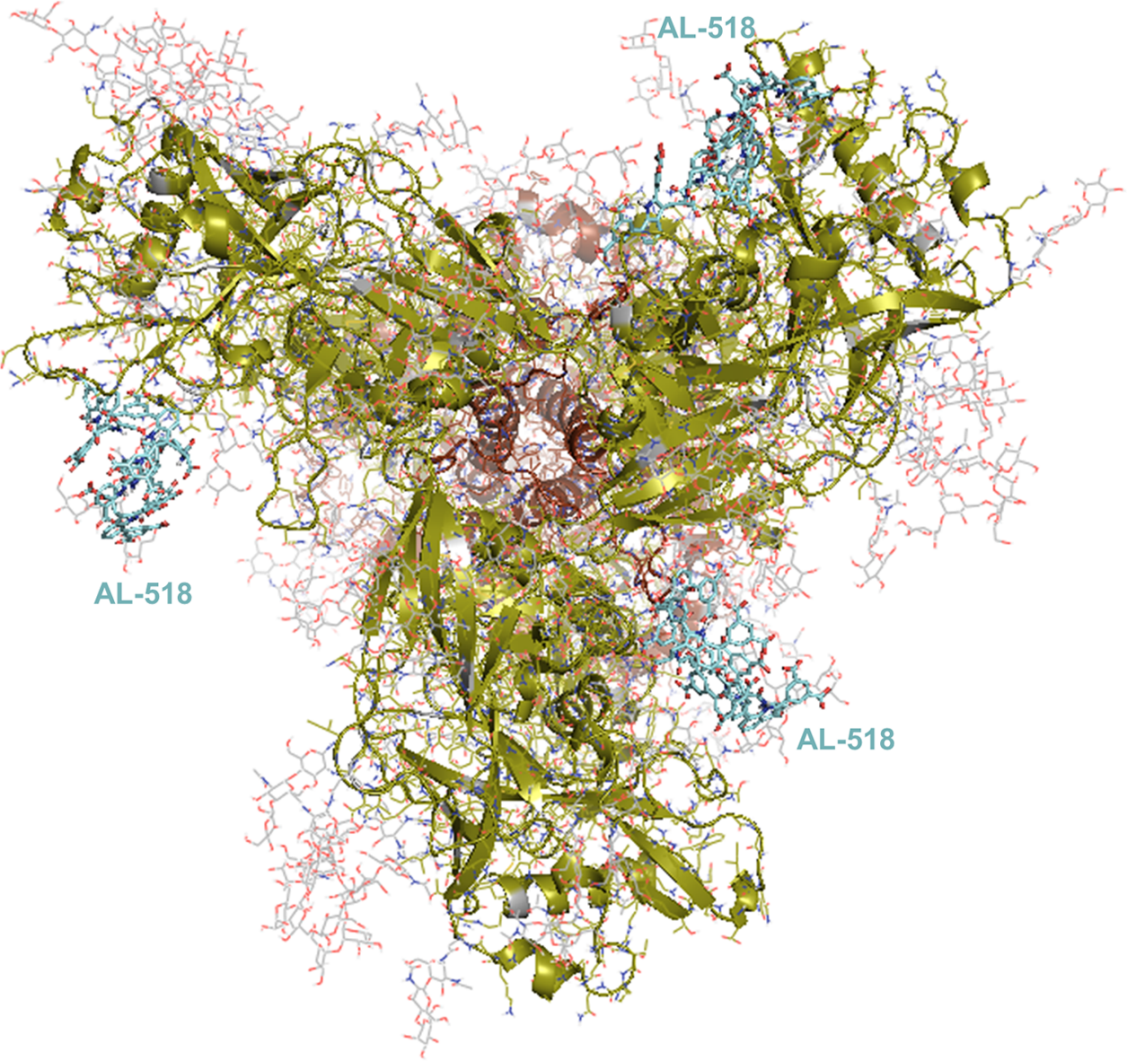
Observed binding modes for three **33** (**AL-518**) molecules (sticks, C atoms in cyan) after 400 ns of unrestrained molecular dynamics simulations of the complex. Note that this top view corresponds to a 90° rotation about the *X*-axis with respect to the lateral view shown in Figure 6.

Our molecular dynamics simulation results show (i) the occurrence of multiple patterns of simultaneous CH···π stacking and hydrogen-bonding interactions between the carbohydrate units—mostly the central mannose in the α-Man-(1 → 3)-[α-Man-(1 → 6)]-Man trisaccharide—and the isophthalic acid-decorated Trp residues. These interactions are enhanced and strengthened when the isophthalic acid moiety is attached not only to C2 but also to the C7 position (Figure 7 and Movie S1); (ii) the broad protein surface coverage provided by the highly mobile gp120 and gp41 glycan “cloud” (Movie S2), which is likely to be encountered by the approaching ligands; and (iii) the targeting of gp120 by the Trp-decorated branches radiating from the **33** (**AL-518**) core using various angles of approach and alternative accommodations within the glycans surrounding the V3 loop (Figure 8 and Movie S3). It is worth noting that C2/C7 arylation on the Trp leads not only to an extended flat surface for stacking interactions relative to a singly C2- or C7-decorated Trp but also to a greater profusion and higher local density of carboxylic acid moieties that can engage in ionic bridges and hydrogen bonds with numerous sugar hydroxyls and protein side chains.

After 400 ns of complex equilibration, each **33** (**AL-518**) molecule appeared preferentially bound to a location close to the V3 loop of gp120, where it establishes highly exchangeable interactions with the oligomannose glycans attached to Asn residues 134, 197, and 363, as well as with the side-chain guanidinium moieties of Arg151 and Arg469. Interestingly, this putative binding mode would preclude engagement of CD4 by gp120 and its embrace by the glycans attached to Asn residues 197 and 276 (Movie S4). Thus, this mechanism would operate similarly to, but distinctly from, that used by CD4 mimetics that directly bind to the so-called “Phe43 cavity” at the interface between the inner and outer gp120 domains (Figure S1). In fact, it can be thought that **33** (**AL-518**) actually mimics bnAbs because the tetrapodal scaffold (i) facilitates both broad glycan recognition and ready access to parts of the protein surface of gp120 and (ii) tolerates microheterogeneity at glycosylation sites.

## Conclusions

The present work built on results from a recently described novel class of tripodal and tetrapodal Trp-containing compounds that bear one or two carboxylic acid groups on a phenyl ring directly attached to the C2 position of every indole ring and potently block cell entry not only of HIV but also of the unrelated EV71 virus. The new strategy consisted of transferring the carboxylic acid containing aryl moiety from C2 to C7. To selectively synthesize the C7-arylated compounds, we took advantage of the Movassaghi and Maleczka methods that allow 2,7-diborylation of Trp followed by in situ bismuth acetate-catalyzed C2 protodeboronation. The next step relied on the Suzuki–Miyaura cross-coupling reaction to form the C–C bond between the indole core and the carboxylic acid aryl moiety, followed by coupling of the arylated Trp to the corresponding tripodal or tetrapodal central scaffold.

The most potent compound, **33** (**AL-518**) (EC_50_ HIV: 6 nM; EC_50_ EV71: 40 nM), is a tetrapodal Trp derivative that is doubly arylated with isophthalic acid moieties at positions C2 and C7 of each indole ring. SPR studies revealed that **33** (**AL-518**) binds to HIV-1 gp120 with greater affinity than prototype **AL-471**, in good accord with its superior anti-HIV-1 activity in the cellular assay. We show that **33** (**AL-518**) inhibits both X4- and R5-tropic viral strains with comparable efficacies. This implicates that the target of interaction on gp120 must be rather conserved (i.e., N-glycans, but not excluding other interaction points with gp120 as well). Competition experiments with antibodies directed to specific regions of the gp120 V3 loop suggest that **33** (**AL-518**) interacts with the same epitopes as mAbs 447-52D and 9284 (the tip and amino-terminal side of the gp120 V3 loop). Molecular modeling showed that **33** (**AL-518**) could interact with these regions through multiple hydrogen bonds and CH···π interactions and may be through electrostatic interactions with arginine residues. Moreover, **33** (**AL-518**), like the prototype, **AL-471**, could interact with the conserved cluster of oligomannose glycans or the “high-mannose patch” that shields the HIV envelope.

The surfaces of EV71 and HIV-1 are covered by receptor-binding proteins that make up either icosahedral capsids or envelope spikes, both of which mediate specific and multivalent interactions with the host cell membranes. The multivalency concept can be successfully applied in reverse to block viral entry, as shown here by the identification of **33** (**AL-518**) as the most potent member of an expanding family of antiviral tetrapodal Trp derivatives. This compound stands out as an important novel prototype for further development of similar tripodal and tetrapodal molecules with the ability to block the cellular entry of other viruses that share common structural features on their external surfaces.

## Experimental Section

### Synthesis

#### General Chemistry Procedures

Commercial reagents and solvents were used as received from the suppliers without further purification unless otherwise stated. The solvents used in some reactions were dried prior to use. Dry dimethylformamide (DMF) was commercially available (Aldrich).

Analytical thin-layer chromatography (TLC) was performed on aluminum plates precoated with silica gel 60 (F_254_, 0.20 mm). Products were visualized using an ultraviolet lamp (254 and 365 nm) or by heating after treatment with a 5% solution of phosphomolybdic acid (PMA) or vanillin in ethanol.

The compounds were purified by (a) high-performance flash chromatography (HPFC) with an “Isolera One” (Biotage) system in reverse phase using water/acetonitrile (100:0–0:100) as the eluent, (b) flash column chromatography on silica gel (60 Merck 230–400 mesh), (c) preparative centrifugal circular thin-layer chromatography (CCTLC) on a Chromatotron (Kiesegel 60 PF254 gipshaltig, Merck) with a layer thickness of 1 mm and a flow rate of 2–4 mL/min.

For HPLC analysis, an Agilent Technologies 1120 Compact LC with a reverse-phase column ACE 5 C18-300 (4.6 mm × 150 mm, 3.5 μm) equipped with a photodiode array (PDA) detector was used. Acetonitrile was used as mobile phase A, and water with 0.05% of trifluoroacetic acid (TFA) was used as mobile phase B with a flow rate of 1 mL·min^–1^. All retention times are quoted in minutes, and the gradients are specified for each compound in the experimental data.

For high-resolution mass spectrometry (HRMS), an Agilent 6520 Accurate Mass quadrupole time-of-flight (QTOF) platform coupled with LC/MS and equipped with an electrospray interface (ESI) working in the positive-ion (ESI^+^) and negative-ion (ESI^–^) modes was used.

NMR spectra (^1^H, ^13^C NMR) were recorded on Varian UNIT INOVA-300 (300 MHz), Bruker AVANCE 300 (300 and 75 MHz), Varian INOVA-400 (400 and 100 MHz), Varian MERCURY-400 (400 and 100 MHz), and Varian-500 (500 and 125 MHz) spectrometers using (CD_3_)_2_SO and CDCl_3_ as solvents. Chemical shift (δ) values are reported in parts per million (ppm) relative to tetramethylsilane (TMS) in ^1^H and CDCl_3_ (δ = 77.0) in ^13^C NMR. Coupling constants (*J* values) are reported in hertz (Hz) and multiplicities of signals are indicated by the following symbol: s (singlet), d (doublet), t (triplet), q (quadruplet), m (multiplet), and bs (broad singlet). Some two-dimensional spectra (correlation spectroscopy (COSY), heteronuclear single quantum coherence (HSQC), and heteronuclear multiple bond correlation (HMBC)) were obtained to identify the structure. The final compounds were lyophilized using a Telstar Lyoquest-85 system. They had purities >95% based on HPLC, LC/MS, and ^1^H NMR analyses.

### General Coupling Procedure for the Synthesis of OMe-Protected Tripodal and Tetrapodal Trp Derivatives

To a solution in DMF (20 mL) containing the corresponding tri- or tetrapodal polyacid **10**(45) or **17**(46,47) (1 mmol), hexafluorophosphate azabenzotriazole tetramethyl uronium (HATU) (1.2 equiv of each carboxylic acid group), and the corresponding Trp C7 or C2/C7 aryl intermediate (1.2 equiv of each carboxylic acid group), *N*,*N*-diisopropylethylamine (DIPEA) (2.4 equiv of each carboxylic acid group) was added. The reaction mixture was stirred at 30 °C for 24 h and then evaporated to dryness. The residue was dissolved in ethyl acetate (20 mL) and washed successively with aqueous solutions of citric acid (10%) (3 × 20 mL), saturated NaHCO_3_ (3 × 20 mL), and brine (3 × 20 mL). The organic phase was dried over anhydrous Na_2_SO_4_, filtered, and evaporated to dryness. The residue was purified on a centrifugal thin-layer chromatography (CCTLC) purification system on normal phase using methanol/dichloromethane (0:100–20:80) or hexane/ethyl acetate (100:0–0:100) as the eluent to give the corresponding tri- or tetrapodal Trp intermediate.

### General Procedure for Methyl Ester Deprotection

To a solution containing the corresponding methyl ester derivative (1.0 mmol) in tetrahydrofuran (THF) (10 mL) at 0 °C (ice bath), a solution of LiOH·H_2_O (2 equiv for each methyl ester group) in water (2 mL) was added, and the mixture was stirred at 30 °C overnight. Then, 1 N hydrochloric acid aqueous solution was added to reach pH ∼ 2, and volatiles were evaporated to dryness. The residue was dissolved in isobutanol (15 mL) and washed with brine (3 × 10 mL) and water (3 × 10 mL). The organic phase was dried over anhydrous Na_2_SO_4_, filtered, and evaporated to dryness. The residue was purified with a Biotage high-performance flash chromatography (HPFC) purification system on reverse phase using water/acetonitrile (100:0–0:100) as the eluent, frozen, and lyophilized, yielding the product as a fluffy powder.

#### (*S*)-Methyl 2-((*tert*-Butoxycarbonyl)amino)-3-(7-(4,4,5,5-tetramethyl-1,3,2-dioxaborolan-2-yl)-1*H*-indol-3-yl)propanoate (**3**)

Commercially available *N*-Boc-l-tryptophan methyl ester **1** (600 mg, 1.88 mmol, 1.00 equiv), (1,5-cyclooctadiene)(methoxy)iridium(I) dimer ([Ir(cod)OMe]_2_) (31.2 mg, 0.047 mmol, 2.5 mol %), bis(pinacolato)diboron (1.19 g, 4.70 mmol, 2.5 equiv), and 4,4′-di-*tert*-butyl-2,2′-bipyridine (d*^t^*bpy) (25.2 mg, 0.094 mmol, 5 mol %) were sealed in a dry reaction vial (microwave reactor vessel can also be used) equipped with a magnetic stirring bar under an argon atmosphere, and anhydrous tetrahydrofuran (5 mL) was added. The resulting red solution was stirred at 60 °C for 12 h. After cooling to room temperature and removal of volatiles under reduced pressure, the subsequent brown residue (intermediate **2**) was dissolved in MeOH/THF (5/4 mL) and bismuth(III) acetate (145.4 mg, 0.38 mmol, 20 mol %) was added. The mixture was stirred under argon at 80 °C for 12 h, then cooled to room temperature, and filtered through Whatman filter paper 42 that was washed three times with ethyl acetate. The filtrate was washed with brine (50 mL), and the organic layer was dried over anhydrous Na_2_SO_4_, filtered, and concentrated under reduced pressure. The resulting brown residue was purified by flash column chromatography on silica gel using hexane/ethyl acetate (4:1) as the eluent to afford 7-borotryptophan derivative **3** (451 mg, 47%) as a white powder. The melting point and spectroscopic data of **3** were consistent with those found in the literature.^41^

### General Procedure for C7 Arylation of Indole

A dry reaction vial (microwave reactor vessel can also be used) was charged sequentially with 7-borotryptophan **3**(41) (150 mg, 0.34 mmol, 1.00 equiv), tris(dibenzylideneacetone)dipalladium(0) (Pd_2_(dba)_3_) (15 mg, 0.02 mmol, 5 mol %), SPhos (28 mg, 0.07 mmol, 10 mol %), and tribasic potassium phosphate (215 mg, 1.01 mmol, 2.00 equiv); sealed; and placed under an argon atmosphere. Then, anhydrous toluene (3 mL) and the corresponding aryl bromide (1.20 equiv) were added via a syringe. The reaction mixture was stirred at 80 °C for 12 h. After being cooled to room temperature, the resulting suspension was filtered through Whatman filter paper 42 that was washed three times with ethyl acetate (20 mL). The filtrate was washed with brine (3 × 20 mL), and the organic layer was dried over anhydrous Na_2_SO_4_, filtered, and concentrated under reduced pressure. The resulting brown residue was purified by CCTLC using hexane/ethyl acetate (7:3) as the eluent to afford the corresponding C7 aryl tryptophan intermediate.

#### (*S*)-Methyl 2-((*tert*-Butoxycarbonyl)amino)-3-(7-(3-methoxycarbonyl-1-phenyl)-1*H*-indol-3-yl)propanoate (**4**)

Following the general C7 arylation procedure, compound **3**(41) (150 mg, 0.34 mmol, 1.00 equiv), tris(dibenzylideneacetone)dipalladium (Pd_2_(dba)_3_) (15 mg, 0.02 mmol, 5 mol %), SPhos (28 mg, 0.07 mmol, 10 mol %), tribasic potassium phosphate (215 mg, 1.01 mmol, 2.00 equiv), and methyl 3-bromobenzoate (87 mg, 0.40 mmol, 1.20 equiv) afforded **4** (215 mg, 70%) as a white amorphous solid. ^1^H NMR (300 MHz, CDCl_3_) δ: 8.36 (s, 1H, NH-1*^i^*Trp), 8.33 (m, 1H, Ar), 8.09 (dt, *J* = 7.8, 1.5 Hz, 1H, Ar), 7.83 (dt, *J* = 7.8, 1.5 Hz, 1H, Ar), 7.60 (m, 2H, Ar), 7.26 (m, 2H, Ar), 7.07 (m, 1H, Ar), 5.14 (d, *J* = 8.3 Hz, 1H, NHCO), 4.70 (m, 1H, α-CHTrp), 3.97 (s, 3H, OCH_3_), 3.73 (s, 3H, OCH_3_), 3.35 (t, *J* = 4.4 Hz, 2H, β-CH_2_Trp), 1.46 (s, 9H, CH_3_). HPLC [gradient: H_2_O/MeCN 10–100% of MeCN in 10 min]: 9.700 min.

#### (*S*)-Methyl 2-((*tert*-Butoxycarbonyl)amino)-3-(7-(4-methoxycarbonyl-1-phenyl)-1*H*-indol-3-yl)propanoate (**5**)

Following the general C7 arylation procedure, 7-borotryptophan **3**(41) (170 mg, 0.38 mmol, 1.00 equiv), tris(dibenzylideneacetone)dipalladium (Pd_2_(dba)_3_) (17.53 mg, 0.02 mmol, 5 mol %), SPhos (16 mg, 0.04 mmol, 10 mol %), tribasic potassium phosphate (163 mg, 0.77 mmol, 2.00 equiv), and methyl 4-bromobenzoate (99 mg, 0.46 mmol, 1.20 equiv) afforded **5** (215 mg, 70%) as a white amorphous solid. ^1^H NMR (400 MHz, CDCl_3_) δ: 8.33 (s, 1H, NH-1*^i^*Trp), 8.17 (m, 2H, Ar), 7.70 (d, *J* = 8.1 Hz, 2H, Ar), 7.59 (dd, *J* = 7.2, 1.8 Hz, 1H, Ar), 7.23 (m, 1H, Ar), 7.05 (m, 1H, Ar), 5.10 (d, *J* = 8.2 Hz, 1H, NHCO), 4.67 (m, 1H, α-CHTrp), 3.96 (s, 3H, OCH_3_), 3.71 (s, 3H, OCH_3_), 3.32 (m, 2H, β-CH_2_Trp), 1.43 (s, 9H, CH_3_). HPLC [gradient: H_2_O/MeCN, 10–100% of MeCN, in 10 min]: 9.800 min.

#### (*S*)-Methyl 2-((*tert*-Butoxycarbonyl)amino)-3-(7-(3,5-dimethoxycarbonyl-1-phenyl)-1*H*-indol-3-yl)propanoate (**6**)

Following the general C7 arylation procedure, 7-borotryptophan **3**(41) (150 mg, 0.34 mmol, 1 equiv), tris(dibenzylideneacetone)dipalladium (Pd_2_(dba)_3_) (15.46 mg, 16.9 μmol, 5 mol %), SPhos (28 mg, 67 μmol, 10 mol %), tribasic potassium phosphate (215 mg, 1.01 mmol, 3.00 equiv), and dimethyl 5-bromoisophthalate (110 mg, 0.40 mmol, 1.20 equiv) afforded **6** (96 mg, 56%) as a white amorphous solid. ^1^H NMR (400 MHz, CDCl_3_) δ: 8.68 (t, *J* = 1.6 Hz, 1H, Ar), 8.48 (d, *J* = 1.7 Hz, 2H, Ar), 8.38 (bs, 1H, NH-1*^i^*Trp), 7.60 (dd, *J* = 7.4, 1.6 Hz, 1H, Ar), 7.28–7.20 (m, 2H, Ar), 7.06 (d, *J* = 2.4 Hz, 1H, Ar), 5.11 (d, *J* = 8.2 Hz, 1H, NHCO), 4.72–4.64 (m, 1H, α-CHTrp), 3.97 (s, 6H, OCH_3_), 3.70 (s, 3H, OCH_3_), 3.34 (m, 2H, β-CH_2_Trp), 1.43 (s, 9H, CH_3_). HPLC [gradient: H_2_O/MeCN, 10–100% of MeCN in 10 min]: 9.613 min.

#### (*S*)-Methyl 2-Amino-3-(7-(3-methoxycarbonyl-1-phenyl)-1*H*-indol-3-yl)propanoate (**7**)

To a cold (0 °C) solution of compound **4** (207 mg, 0.46 mmol) in dichloromethane (10 mL), TFA (0.5 mL) was added. The mixture was stirred at room temperature for 6 h. Then, volatiles were evaporated to dryness and the residue was coevaporated successively with methanol and dichloromethane to afford compound **7** (245 mg, quant.) in the salt form. ^1^H NMR (300 MHz, dimethyl sulfoxide (DMSO)-*d*_6_) δ: 11.10 (s, 1H, NH-1*^i^*Trp), 8.39 (s, 3H, NH_3_^+^), 8.17 (t, *J* = 1.8 Hz, 1H, Ar), 8.01 (dt, *J* = 7.8, 1.5 Hz, 1H, Ar), 7.88 (d, *J* = 7.8 Hz, 1H, Ar), 7.69 (t, *J* = 7.8 Hz, 1H, Ar), 7.54 (m, 1H, Ar), 7.25 (d, *J* = 2.6 Hz, 1H, Ar), 7.17 (m, 2H, Ar), 4.36 (m, 1H, α-CHTrp), 3.89 (s, 3H, OCH_3_), 3.73 (s, 3H, OCH_3_), 3.31 (d, *J* = 6.0 Hz, 2H, β-CH_2_Trp). HPLC [gradient: H_2_O/MeCN, 10–100% of MeCN in 10 min]: 6.724 min.

#### (*S*)-Methyl 2-Amino-3-(7-(4-methoxycarbonyl-1-phenyl)-1*H*-indol-3-yl)propanoate (**8**)

A cold (0 °C) solution of compound **5** (120 mg, 0.27 mmol) in dichloromethane (10 mL) was treated with TFA (0.5 mL) as described for **7** to afford **8** (122 mg, 99%) in the salt form. ^1^H NMR (300 MHz, DMSO-*d*_6_) δ: 11.08 (d, *J* = 2.8 Hz, 1H, NH-1*^i^*Trp), 8.40 (s, 3H, NH_3_^+^), 8.10 (d, *J* = 8.3 Hz, 2H, Ar), 7.78 (d, *J* = 8.3 Hz, 2H, Ar), 7.57 (d, *J* = 7.6 Hz, 1H, Ar), 7.20 (m, 3H, Ar), 4.34 (m, 1H, α-CHTrp), 3.90 (s, 3H, OCH_3_), 3.72 (s, 3H, OCH_3_), 3.31 (d, *J* = 6.2 Hz, 2H, β-CH_2_Trp). HPLC [gradient: H_2_O/MeCN, 10–100% of MeCN in 10 min]: 6.601 min.

#### (*S*)-Methyl 2-Amino-3-(7-(3,5-dimethoxycarbonyl-1-phenyl)-1*H*-indol-3-yl)propanoate (**9**)

A cold solution (0 °C) of compound **6** (170 mg, 0.33 mmol) in dichloromethane (15 mL) was treated with TFA (1 mL) as described for **7** to afford **9** (172.3 mg, quant.) in the salt form. ^1^H NMR (300 MHz, DMSO-*d*_6_) δ: 11.19 (d, *J* = 2.9 Hz, 1H, NH-1*^i^*Trp), 8.51 (t, *J* = 1.6 Hz, 1H, Ar), 8.39 (s, 3H, NH_3_^+^), 8.37 (d, *J* = 1.6 Hz, 2H, Ar), 7.59 (dd, *J* = 6.9, 2.2 Hz, 1H, Ar), 7.29 (d, *J* = 2.5 Hz, 1H, Ar), 7.24–7.16 (m, 2H, Ar), 4.35 (m, α-CHTrp), 3.92 (s, 6H, OCH_3_), 3.73 (s, 3H, OCH_3_), 3.32 (m, 2H, β-CH_2_Trp). HPLC [gradient: H_2_O/MeCN, 10–100% of MeCN in 10 min]: 6.798 min.

#### Tripodal **11**

Following the general coupling procedure, a solution of the tripodal polyacid **10**(45) (16 mg, 0.59 μmol, 1.00 equiv), HATU (81 mg, 0.21 mmol, 3.60 equiv), and intermediate **7** (100 mg, 0.21 mmol, 3.60 equiv) in dry DMF (20 mL) was treated with DIPEA (74 μL, 0.49 mmol, 7.20 equiv) to afford compound **11** (37 mg, 48%) as an amorphous white solid. ^1^H NMR (400 MHz, CDCl_3_) δ: 8.69 (d, *J* = 2.6 Hz, 3H, NH-1*^i^*Trp), 8.25 (t, *J* = 1.8 Hz, 3H, Ar), 8.00 (dt, *J* = 7.8, 1.4 Hz, 3H, Ar), 7.75 (dt, *J* = 7.8, 1.4 Hz, 3H, Ar), 7.57–7.39 (m, 6H, Ar), 7.22–7.11 (m, 5H, Ar), 6.94 (d, *J* = 2.4 Hz, 3H, NHCO), 4.74 (m, 3H, α-CHTrp), 3.87 (s, 9H, OCH_3_), 3.62 (s, 9H, OCH_3_), 3.23 (m, 6H, β-CH_2_Trp), 2.06–1.91 (m, 12H, OCH_2_). HPLC [gradient: H_2_O/MeCN, 40–100% of MeCN in 10 min]: 9.110 min.

#### Tripodal **12**

Following the general coupling procedure, a solution of the tripodal polyacid **10**(45) (7 mg, 0.24 μmol, 1.00 equiv), HATU (33 mg, 0.09 mmol, 3.60 equiv), and intermediate **8** (40 mg, 0.09 mmol, 3.60 equiv) in dry DMF (20 mL) was treated with DIPEA (30 μL, 0.17 mmol, 7.20 equiv) to afford compound **12** (14 mg, 48%) as an amorphous white solid. ^1^H NMR (400 MHz, CDCl_3_) δ: 8.68 (s, 3H, NH-1*^i^*Trp), 8.11 (d, *J* = 8.0 Hz, 6H, Ar), 7.64 (d, *J* = 8.0 Hz, 6H, Ar), 7.48 (dd, *J* = 7.3, 1.7 Hz, 3H, Ar), 7.18 (m, 6H, Ar), 6.95 (d, *J* = 2.4 Hz, 3H, Ar), 6.29 (d, *J* = 7.9 Hz, 3H, NHCO), 4.83 (m, 3H, α-CHTrp), 3.93 (s, 9H, OCH_3_), 3.65 (s, 9H, OCH_3_), 3.24 (m, 6H, β-CH_2_Trp), 2.11–2.02 (m, 12H, OCH_2_). HPLC [gradient: H_2_O/MeCN, 40–100% of MeCN in 10 min]: 9.210 min.

#### Tripodal **13**

Following the general coupling procedure, a solution of the tripodal polyacid **10**(45) (15 mg, 0.05 mmol, 1.00 equiv), HATU (76 mg, 0.20 mmol, 3.60 equiv), and intermediate **9** (105 mg, 0.21 mmol, 3.60 equiv) in dry DMF (20 mL) was treated with DIPEA (69 μL, 0.40 mmol, 7.20 equiv) to afford compound **13** (57 mg, 69%) as an amorphous white solid. ^1^H NMR (400 MHz, CDCl_3_) δ: 8.86 (d, *J* = 2.5 Hz, 3H, NH-1*^i^*Trp), 8.61 (t, *J* = 1.6 Hz, 3H, Ar), 8.41 (d, *J* = 1.7 Hz, 6H, Ar), 7.49 (dd, *J* = 5.2, 3.8 Hz, 3H, Ar), 7.15 (q, *J* = 2.6, 1.7 Hz, 6H, Ar), 6.97 (d, *J* = 2.4 Hz, 3H, Ar), 6.41 (d, *J* = 7.9 Hz, 3H, NHCO), 4.83 (m, 3H, α-CHTrp), 3.90 (s, 18H, OCH_3_), 3.63 (s, 9H, OCH_3_), 3.23 (m, 6H, β-CH_2_Trp), 2.10 (m, 12H, CH_2_). HPLC [gradient: H_2_O/MeCN, 40–100% of MeCN in 10 min]: 9.300 min.

#### Tripodal **14**

Following the general procedure for methyl ester deprotection, intermediate **11** (37 mg, 0.03 mmol, 1.00 equiv) and LiOH·H_2_O (16 mg, 0.37 mmol, 12.00 equiv) gave **14** (38 mg, quant.) as an amorphous white solid. ^1^H NMR (500 MHz, DMSO-*d*_6_) δ: 10.82 (s, 3H, NH-1*^i^*Trp), 8.28 (d, *J* = 7.7 Hz, 3H, NHCO), 8.13 (s, 3H, Ar), 7.95 (d, *J* = 7.6 Hz, 3H, Ar), 7.82 (d, *J* = 7.6 Hz, 3H, Ar), 7.61 (t, *J* = 7.7 Hz, 3H, Ar), 7.55 (m, 3H, Ar), 7.14 (d, *J* = 2.5 Hz, 3H, Ar), 7.10 (d, *J* = 4.6 Hz, 4H, Ar), 4.46 (m, 3H α-CHTrp), 3.18 (m, 3H, OCH_3_), 3.03 (m, 6H, β-CH_2_Trp), 2.04 (m, 12H, OCH_2_). ^13^C NMR (126 MHz, DMSO-*d*_6_) δ: 173.4, 170.7, 167.4, 139.1, 133.2, 132.6, 131.5, 129.3, 129.0, 128.3, 128.0, 124.6, 124.3, 121.3, 119.2, 118.1, 110.5, 93.3, 65.0, 53.2, 45.9, 30.7, 29.3, 27.1. HPLC [gradient: H_2_O/MeCN, 10–100% of MeCN in 10 min]: 7.510 min. HRMS (ESI^–^) *m*/*z*: calcd for C_64_H_57_N_7_O_17_ 1195.38109; found 1195.38089.

#### Tripodal **15**

Following the general procedure for methyl ester deprotection, intermediate **12** (15 mg, 0.01 mmol, 1.00 equiv) and LiOH·H_2_O (8 mg, 0.18 mmol, 18.00 equiv) gave **15** (7 mg, 54%) as an amorphous white solid. ^1^H NMR (500 MHz, DMSO-*d*_6_) δ: 10.83 (d, *J* = 2.5 Hz, 3H, NH-1*^i^*Trp), 8.26 (d, *J* = 7.7 Hz, 3H, NHCO), 8.05 (d, *J* = 7.8 Hz, 6H, Ar), 7.72 (d, *J* = 7.8 Hz, 4H, Ar), 7.57 (d, *J* = 7.8 Hz, 4H, Ar), 7.15 (m, 5H, Ar), 7.10 (t, *J* = 7.5 Hz, 4H), 4.48 (m, 3H, α-CHTrp), 3.18 (dd, *J* = 14.3, 7.3 Hz, 3H, β-CH_2_Trp), 3.03 (dd, *J* = 14.7, 8.4 Hz, 3H, β-CH_2_Trp), 2.12–1.92 (m, 12H, OCH_2_), 1.24 (m, 4H, OCH_2_). ^13^C NMR (126 MHz, DMSO-*d*_6_) δ: 173.3, 170.6, 167.2, 143.2, 133.1, 129.9, 129.3, 128.4, 128.3, 124.6, 124.1, 121.4, 119.1, 118.5, 110.6, 53.2, 30.4, 29.3, 29.0, 27.1. HPLC [gradient: H_2_O/MeCN, 10–100% of MeCN in 10 min]: 6.924 min. HRMS (ESI^–^) *m*/*z*: calcd for C_64_H_57_N_7_O_17_ 1195.38109; found 1195.38042.

#### Tripodal **16**

Following the general procedure for methyl ester deprotection, intermediate **13** (12 mg, 8.25 μmol, 1.00 equiv) and LiOH·H_2_O (6 mg, 0.14 mmol, 18.00 equiv) gave **16** (6.6 mg, 60%) as an amorphous white solid. ^1^H NMR (300 MHz, DMSO-*d*_6_) δ: 10.92 (s, 3H, NH-1*^i^*Trp), 8.48 (s, 3H, Ar), 8.31 (m, 6, Ar), 8.27 (m, 3H, NHCO), 7.59 (m, 3H, Ar), 7.17 (m, 3H, Ar), 7.13 (s, 6H, Ar), 4.49 (m, 3H, α-CHTrp), 3.16 (m, 3H, β-CH_2_Trp), 3.05 (dd, *J* = 14.3, 7.6 Hz, 3H, β-CH_2_Trp), 2.07 (m, 12H, OCH_2_). ^13^C NMR (126 MHz, DMSO-*d*_6_) δ: 173.4, 170.7, 167.0, 139.4, 133.2, 132.7, 128.6, 128.4, 124.7, 123.7, 121.4, 119.2, 118.4, 110.7, 93.3, 64.9, 53.2, 30.6, 29.3, 27.8, 27.1. HPLC [gradient: H_2_O/MeCN, 10–100% of MeCN in 10 min]: 6.020 min. HRMS (ESI^–^) *m*/*z*: calcd for C_67_H_57_N_7_O_23_ 1327.35058; found 1327.35014.

#### Tetrapodal **18**

Following the general coupling procedure, compound **17**(46,47) (25 mg, 0.05 mmol, 1.00 equiv), HATU (105 mg, 0.27 mmol, 4.80 equiv), intermediate **7** (130 mg, 0.31 mmol, 4.80 equiv) and DIPEA (87 μL, 0.58 mmol, 10.00 equiv) afforded **18** (33.2 mg, 33%) as an amorphous white solid. ^1^H NMR (400 MHz, CDCl_3_) δ: 9.10 (d, *J* = 2.4 Hz, 4H, NH-1*^i^*Trp), 8.25 (t, *J* = 1.7 Hz, 4H, Ar), 7.98 (dt, *J* = 7.8, 1.4 Hz, 4H, Ar), 7.77 (dt, *J* = 7.8, 1.4 Hz, 4H, Ar), 7.48 (m, 8H, Ar), 7.14 (m, 8H, Ar), 6.93 (d, *J* = 2.3 Hz, 4H, Ar), 6.67 (d, *J* = 7.8 Hz, 4H, NHCO), 4.84 (m, 4H, α-CHTrp), 3.86 (s, 12H, OCH_3_), 3.60 (s, 12H, OCH_3_), 3.24 (m, 16H, OCH_2_), 3.03 (d, *J* = 9.3 Hz, 4H, β-CH_2_Trp), 2.86 (d, *J* = 9.3 Hz, 4H, β-CH_2_Trp), 2.20 (ddd, *J* = 15.5, 7.0, 4.4 Hz, 4H, OCH_2_), 2.12 (ddd, *J* = 15.5, 7.0, 4.4 Hz, 4H, OCH_2_). HPLC [gradient: H_2_O/MeCN, 40–100% of MeCN in 10 min]: 7.800 min.

#### Tetrapodal **19**

Following the general coupling procedure, compound **17**(46,47) (23 mg, 0.05 mmol, 1.00 equiv), HATU (99 mg, 0.26 mmol, 4.80 equiv), intermediate **8** (122 mg, 0.26 mmol, 4.80 equiv), and DIPEA (89 μL, 0.54 mmol, 10.00 equiv) afforded **19** (56 mg, 59%) as an amorphous white solid. ^1^H NMR (300 MHz, CDCl_3_) δ: 9.39 (m, NH^1^-Trp), 8.19 (m, 8H, Ar), 7.76 (d, *J* = 7.2 Hz, 8H, Ar), 7.57 (dt, *J* = 7.3, 1.7 Hz, 4H, Ar), 7.22 (m, 4H, Ar), 7.06 (d, *J* = 2.2 Hz, 4H, Ar), 6.84 (m, 4H, Ar), 4.93 (m, 4H, α-CHTrp), 4.00 (s, 12H, OCH_3_), 3.71 (s, 12H, OCH_3_), 3.34 (m, 16H, OCH_2_ and β-CH_2_Trp), 3.14 (d, *J* = 9.2 Hz, 4H, OCH_2_), 2.94 (d, *J* = 9.3 Hz, 4H, OCH_2_), 2.24 (m, 8H, CH_2_). HPLC [gradient: H_2_O/MeCN, 70–100% of MeCN in 10 min]: 7.208 min.

#### Tetrapodal **20**

Following the general coupling procedure, compound **17**(46,47) (26 mg, 0.06 mmol, 1.00 equiv), HATU (116 mg, 0.31 mmol, 4.80 equiv), intermediate **9** (160 mg, 0.31 mmol, 4.80 equiv), and DIPEA (130 μL, 0.76 mmol, 12.00 equiv) afforded **20** (68 mg, 54%) as an amorphous white solid. ^1^H NMR (300 MHz, CDCl_3_) δ: 9.26 (d, *J* = 2.5 Hz, 4H, NH^1^-Trp), 8.60 (t, *J* = 1.6 Hz, 4H, Ar), 8.42 (d, *J* = 1.6 Hz, 8H, Ar), 7.49 (dd, *J* = 6.3, 2.8 Hz, 4H, Ar), 7.17–7.09 (m, 8H, Ar), 6.95 (d, *J* = 2.4 Hz, 4H, NHCO), 6.79 (d, *J* = 7.9 Hz, 4H, Ar), 4.81 (m, 4H, α-CHTrp), 3.90 (s, 24H, OCH_3_), 3.60 (s, 12H, OCH_3_), 3.28 (m, 16H, OCH_2_ and β-CH_2_Trp), 3.08 (d, *J* = 9.3 Hz, 4H, OCH_2_), 2.93 (d, *J* = 9.3 Hz, 4H, OCH_2_), 2.33–2.13 (m, 8H, CH_2_). HPLC [gradient: H_2_O/MeCN, 10–100% of MeCN in 10 min]: 8.658 min.

#### Tetrapodal **21**

Following the general procedure for methyl ester deprotection, intermediate **18** (33 mg, 0.02 mmol) and LiOH·H_2_O (13 mg, 0.3 mmol, 16.00 equiv) afforded **21** (21 mg, quant) as an amorphous white solid. ^1^H NMR (500 MHz, DMSO-*d*_6_) δ: 10.83 (d, *J* = 2.6 Hz, 4H, NH-1*^i^*Trp), 8.18 (d, *J* = 7.7 Hz, 4H, NHCO) 8.14 (m, 4H, Ar), 7.96 (dt, *J* = 7.7, 1.5 Hz, 4H, Ar), 7.83 (dt, *J* = 7.7, 1.5 Hz, 4H, NHCO), 7.61 (t, *J* = 7.7 Hz, 4H, Ar), 7.56 (dd, *J* = 6.3, 2.7 Hz, 4H, Ar), 7.15 (d, *J* = 2.6 Hz, 4H, Ar), 7.10 (m, 7H, Ar), 4.54 (m, 4H, α-CHTrp), 3.38 (m, 12H, CH_2_), 3.16 (m, 4H, β-CH_2_Trp), 3.05 (m, 4H, β-CH_2_Trp), 2.29 (m, 12H, CH_2_). ^13^C NMR (126 MHz, DMSO-*d*_6_) δ: 173.4, 170.3, 167.4, 139.1, 133.2, 132.6, 131.4, 129.2, 129.1, 128.3, 128.0, 124.6, 124.3, 121.3, 119.2, 118.1, 110.5, 68.8, 67.1, 53.0, 44.9, 35.8, 27.3. HPLC [gradient: H_2_O/MeCN, 10–100% of MeCN in 10 min]: 7.435 min. HRMS (ESI^–^) *m*/*z*: calcd for C_89_H_84_N_8_O_24_ 1648.55985; found 1648.55573.

#### Tetrapodal **22**

Following the general procedure for methyl ester deprotection, intermediate **19** (56 mg, 0.03 mmol, 1.00 equiv) and LiOH·H_2_O (21 mg, 0.050 mmol, 16.00 equiv) afforded **22** (36 mg, 70%) as an amorphous white solid. ^1^H NMR (400 MHz, DMSO-*d*_6_) δ: 10.82 (bs, 4H, NH-1*^i^*Trp), 8.16 (d, *J* = 7.7 Hz, 4H), 8.06 (d, *J* = 8.0 Hz, 8H, Ar), 7.73 (d, *J* = 8.0 Hz, 8H, Ar), 7.58 (d, *J* = 7.7 Hz, 4H, NHCO), 7.18–7.13 (m, 8H, Ar), 7.10 (t, *J* = 7.5 Hz, 3H, Ar), 4.54 (m, 4H, α-CHTrp), 3.47–3.35 (m, 12H, OCH_2_ and β-CH_2_Trp), 3.12 (m, 6H, OCH_2_), 3.05 (m, 4H, β-CH_2_Trp), 2.30 (tq, *J* = 14.5, 7.5, 7.0 Hz, 8H, CH_2_). ^13^C NMR (101 MHz, DMSO-*d*_6_) δ: 173.3, 170.2, 167.2, 143.2, 133.1, 129.9, 129.3, 128.3, 124.6, 124.1, 121.4, 119.1, 118.5, 110.5, 68.8, 67.7, 67.1, 64.9, 52.9, 44.9, 35.7, 30.5, 27.2, 19.1. HPLC [gradient: H_2_O/MeCN, 10–100% of MeCN in 10 min]: 7.110 min. HRMS (ESI^–^) *m*/*z*: calcd for C_89_H_84_N_8_O_24_ 1648.55985; found 1648.56095.

#### Tetrapodal **23**

Following the general procedure for methyl ester deprotection, intermediate **20** (46 mg, 0.03 mmol, 1.00 equiv) and LiOH·H_2_O (30 mg, 0.72 mmol, 24.00 equiv) afforded **23** (42 mg, quant.) as an amorphous white solid. ^1^H NMR (400 MHz, DMSO-*d*_6_) δ: 10.92 (bs, 4H, NH-1*^i^*Trp), 8.48 (t, *J* = 1.6 Hz, 4H, Ar), 8.32 (d, *J* = 1.6 Hz, 8H, Ar), 8.19 (d, *J* = 7.7 Hz, 4H, NHCO), 7.60 (dd, *J* = 7.0, 2.0 Hz, 4H, Ar), 7.17 (d, *J* = 2.4 Hz, 4H, Ar), 7.16–7.09 (m, 8H, Ar), 4.54 (m, 4H, α-CHTrp), 3.45 (m, 8H, OCH_2_), 3.23–3.15 (m, 12H, OCH_2_ and β-CH_2_Trp), 3.11–3.03 (m, 4H, β-CH_2_Trp), 2.40–2.24 (m, 8H, CH_2_). ^13^C NMR (100 MHz, DMSO-*d*_6_) δ: 173.3, 170.3, 166.6, 139.6, 133.2, 132.9, 132.0, 128.5, 128.4, 124.7, 123.5, 121.4, 119.2, 118.5, 110.6, 68.8, 67.1, 52.9, 44.9, 35.7, 27.3. HPLC [gradient: H_2_O/MeCN, 10–100% of MeCN in 10 min]: 6.198 min. HRMS (ESI^–^) *m*/*z*: calcd for C_93_H_84_N_8_O_32_ 1824.51916; found 1824.52142.

#### Dimethyl 4,4′-(3-(2-((*tert*-Butoxycarbonyl)amino)-3-methoxy-3-oxopropyl)-1*H*-indole-2,7-diyl)dibenzoate (**24**)

Commercially available *N*-Boc-l-tryptophan methyl ester **1** (100 mg, 0.31 mmol, 1.00 equiv) was treated with bis(pinacolato)diboron (199 mg, 0.8 mmol, 1.25 equiv), (1,5-cyclooctadiene) (methoxy)iridium(I) dimer ([Ir(cod)OMe]_2_) (5 mg, 0.008 mmol, 2.5 mol %), and 4,4′-di-*tert*-butyl-2,2′-bipyridine (d*^t^*bpy) (4 mg, 0.016 mmol, 5 mol %) as mentioned above for **3**. The resulting brown residue (intermediate **2**) was treated with tris(dibenzylideneacetone)dipalladium (Pd_2_(dba)_3_) (14 mg, 0.014 mmol, 5 mol %), SPhos (12 mg, 0.030 mmol, 10 mol %), tribasic potassium phosphate (253 mg, 1.20 mmol, 4.00 equiv), and methyl 4-bromobenzoate (141 mg, 0.66 mmol, 2.20 equiv). The tube was sealed and placed under an argon atmosphere. Then, anhydrous toluene (3 mL) was added via a syringe. The reaction mixture was stirred at 80 °C for 12 h and worked up as described in the general C7 arylation procedure. The resulting brown residue was purified by CCTLC using dichloromethane/methanol (20:1) as the eluent to afford compound **24** (50 mg, 30%) as a brown amorphous solid. ^1^H NMR (400 MHz, methanol-*d*_4_) δ: 7.94 (m, 4H, Ar), 7.54 (m, 5H, Ar), 7.04 (d, *J* = 3.9 Hz, 2H, Ar), 4.69 (s, 3H, OCH_3_), 4.30 (m, 1H, α-CHTrp), 3.77 (s, 6H, OCH_3_), 3.22 (m, 1H, β-CH_2_Trp), 3.18 (m, 1H, β-CH_2_Trp), 1.17 (s, 9H, CH_3_). HPLC [gradient: H_2_O/MeCN, 10–100% of MeCN in 10 min]: 10.514 min.

#### Tetramethyl 5,5′-(3-(2-((*tert*-Butoxycarbonyl)amino)-3-methoxy-3-oxopropyl)-1*H*-indole-2,7-diyl)diisophthalate (**25**)

Commercially available *N*-Boc-l-tryptophan methyl ester **1** (200 mg, 0.63 mmol, 1.00 equiv) was treated with bis(pinacolato)diboron (399 mg, 1.60 mmol, 2.50 equiv), (1,5-cyclooctadiene) (methoxy)iridium(I) dimer ([Ir(cod)OMe]_2_) (10 mg, 0.016 mmol, 2.5 mol %), and 4,4′-di-*tert*-butyl-2,2′-bipyridine (d*^t^*bpy) (5 mg, 0.031 mmol, 5 mol %) as mentioned above for **3**. The brown residue (intermediate **2**) was treated with tris(dibenzylideneacetone)dipalladium (Pd_2_(dba)_3_) (48 mg, 52.60 μmol, 5 mol %), SPhos (43 mg, 105.20 μmol, 10 mol %), tribasic potassium phosphate (893 mg, 4.20 mmol, 4.00 equiv), and dimethyl 5-bromoisophthalate (632 mg, 2.31 mmol, 2.20 equiv) as described above for **24** to give **25** (190 mg, 43%) as a brown amorphous solid. ^1^H NMR (400 MHz, DMSO-*d*_6_) δ: 11.32 (bs, 1H, NH-1*^i^*Trp), 8.50 (dt, *J* = 11.5, 1.7 Hz, 2H, Ar), 8.40 (d, *J* = 1.7 Hz, 2H, Ar), 8.33 (d, *J* = 1.7 Hz, 2H, Ar), 7.72 (m, 1H, Ar), 7.22 (m, 2H, Ar), 7.14 (d, *J* = 8.1 Hz, 1H, NHCO), 4.22 (m, 1H, α-CHTrp), 3.93 (m, 12H, OCH_3_), 3.43 (s, 3H OCH_3_), 3.35 (m, 1H, β-CH_2_Trp), 3.21 (m, 1H, β-CH_2_Trp), 1.24 (s, 9H, CH_3_). HPLC [gradient: H_2_O/MeCN, 20–100% of MeCN in 10 min]: 11.380 min.

#### 3-(2,7-Bis(4-(methoxycarbonyl)phenyl)-1*H*-indol-3-yl)-1-methoxy-1-oxopropan-2-aminium 2,2,2-Trifluoro-acetate (**26**)

A cold (0 °C) solution of compound **24** (50 mg, 0.09 mmol) in dichloromethane (5 mL) was treated with TFA (0.25 mL) as described for **7** to afford **26** (47 mg, 91%) in the salt form. ^1^H NMR (300 MHz, CDCl_3_) δ: 8.61 (m, 1H, NH-1*^i^*Trp), 8.13 (d, *J* = 7.9 Hz, 2H, Ar), 8.02 (d, *J* = 7.7 Hz, 2H, Ar), 7.68 (d, *J* = 7.9 Hz, 3H, Ar), 7.54 (d, *J* = 7.7 Hz, 2H, Ar), 7.19 (m, 2H, Ar), 4.19 (m, 1H, α-CHTrp), 3.92 (s, 3H, OCH_3_), 3.85 (s, 3H, OCH_3_), 3.64 (s, 2H, β-CH_2_Trp), 3.35 (s, 3H, OCH_3_). HPLC [gradient: H_2_O/MeCN, 10–100% of MeCN in 10 min]: 7.452 min.

#### 3-(2,7-Bis(3,5-bis(methoxycarbonyl)phenyl)-1*H*-indol-3-yl)-1-methoxy-1-oxopropan-2-aminium 2,2,2-Trifluoroacetate (**27**)

A cold (0 °C) solution of compound **25** (170 mg, 0.24 mmol) in dichloromethane (15 mL) was treated with TFA (0.7 mL) as described for **7** to afford **27** (173 mg, quant.) in the salt form. ^1^H NMR (400 MHz, DMSO-*d*_6_) δ: 11.32 (s, 1H, NH-1*^i^*Trp), 8.50 (dt, *J* = 11.5, 1.7 Hz, 2H, Ar), 8.40 (d, *J* = 1.7 Hz, 2H, Ar), 8.33 (d, *J* = 1.7 Hz, 2H, Ar), 7.72 (m, 1H, Ar), 7.22 (d, *J* = 4.6 Hz, 2H, Ar), 7.14 (d, *J* = 8.1 Hz, 1H, NHCO), 4.22 (m, 1H, α-CHTrp), 3.93 (s, 12H, OCH_3_), 3.44 (s, 3H, OCH_3_), 3.31 (m, 1H, β-CH_2_Trp), 3.22 (m, 1H, β-CH_2_Trp). HPLC [gradient: H_2_O/MeCN, 20–100% of MeCN in 10 min]: 7.698 min.

#### Tripodal **28**

Following the general coupling procedure, a solution of the tripodal polyacid **10**(45) (5 mg, 0.02 mmol, 1.00 equiv), HATU (27 mg, 0.07 mmol, 3.60 equiv), and intermediate **27** (50 mg, 0.07 mmol, 3.60 equiv) in dry DMF (20 mL) was treated with DIPEA (0.025 mL, 0.15 mmol, 8.00 equiv) to afford compound **28** (36 mg, 88%) as an amorphous white solid. ^1^H NMR (400 MHz, CDCl_3_) δ: 8.57 (s, 6H, Ar), 8.52–8.49 (m, 8H, Ar), 8.47 (s, 3H, Ar), 7.62 (d, *J* = 7.8 Hz, 3H, Ar), 7.26 (m, 7H, Ar), 6.25 (m, 3H, NHCO), 4.86 (m, 3H, α-CHTrp), 3.67–3.56 (m, 45H, OCH_3_), 3.52 (m, 6, β-CH_2_Trp), 1.44 (m, 12H, OCH_2_). HPLC [gradient: H_2_O/MeCN, 20–100% of MeCN in 10 min]: 6.393 min.

#### Tetrapodal **29**

Following the general coupling procedure, compound **17**(46,47) (7 mg, 0.02 mmol, 1.00 equiv), HATU (30 mg, 0.08 mmol, 4.80 equiv), intermediate **26** (47 mg, 0.08 mmol, 4.80 equiv), and DIPEA (0.026 mL, 0.16 mmol, 10.00 equiv) afforded compound **29** (15 mg, 40%) as an amorphous white solid. ^1^H NMR (400 MHz, CDCl_3_) δ: 8.72 (s, 4H, NH^1^-Trp), 8.11 (d, *J* = 8.0 Hz, 8H, Ar), 8.03 (d, *J* = 8.0 Hz, 8H, Ar), 7.68 (d, *J* = 8.0 Hz, 8H, Ar), 7.60 (d, *J* = 8.0 Hz, 12H, Ar), 7.22–7.11 (m, 8H, Ar), 6.61 (d, *J* = 7.9 Hz, 4H, NHCO), 4.82 (m, 4H, α-CHTrp), 3.91 (s, 12H, OCH_3_), 3.87 (s, 12H, OCH_3_), 3.46 (m, 8H, β-CH_2_Trp), 3.28 (s, 12H, OCH_3_), 3.21 (dd, *J* = 10.1, 5.4 Hz, 6H, OCH_2_), 3.11–2.97 (m, 8H, OCH_2_), 2.06 (m, 10H, OCH_2_). HPLC [gradient: H_2_O/MeCN, 50–100% of MeCN in 10 min]: 7.208 min.

#### Tetrapodal **30**

Following the general coupling procedure, compound **17**(46,47) (6 mg, 0.01 mmol, 1.00 equiv), HATU (27 mg, 0.07 mmol, 4.80 equiv), intermediate **27** (50 mg, 0.07 mmol, 4.80 equiv), and DIPEA (0.024 mL, 0.14 mmol, 10.00 equiv) afforded compound **30** (22 mg, 54%) as an amorphous solid. ^1^H NMR (400 MHz, CDCl_3_) δ: 9.35 (s, 4H, NH^1^-Trp), 8.53 (s, 4H, Ar), 8.48 (s, 4H, Ar), 8.40 (s, 8H, Ar), 8.30 (s, 8H, Ar), 7.64 (m, 4H, Ar), 7.14 (m, 8H, Ar), 6.74 (d, *J* = 8.0 Hz, 4H, NHCO), 4.82 (m, 4H, α-CHTrp), 3.83 (s, 48H, OCH_3_), 3.38 (m, 16H, β-CH_2_Trp and OCH_2_), 3.26 (s, 12H, OCH_3_), 3.09 (m, 8H, OCH_2_), 2.16 (m, 8H, CH_2_). HPLC [gradient: H_2_O/MeCN, 50–100% of MeCN in 10 min]: 11.996 min.

#### Tripodal **31**

Following the general procedure for methyl ester deprotection, intermediate **28** (30 mg, 0.01 mmol, 1.00 equiv) and LiOH·H_2_O (19 mg, 0.44 mmol, 30 equiv) afforded **31** (24 mg, 89%) as an amorphous white solid. ^1^H NMR (400 MHz, DMSO-*d*_6_) δ: 11.24 (s, 3H, NH-1*^i^*Trp), 8.47 (dt, *J* = 6.9, 1.6 Hz, 4H, Ar), 8.36 (d, *J* = 7.9 Hz, 3H, NHCO), 8.31 (dd, *J* = 8.3, 1.6 Hz, 12H, Ar), 7.74 (dd, *J* = 7.2, 1.9 Hz, 3H, Ar), 7.17 (d, *J* = 7.5 Hz, 6H, Ar), 4.50 (m, 3H, α-CHTrp), 3.29 (m, 3H, β-CH_2_Trp), 3.18 (m, 3H, β-CH_2_Trp), 1.97 (s, 12H, CH_2_). ^13^C NMR (126 MHz, DMSO-*d*_6_) δ: 172.8, 170.4, 166.7, 166.5, 139.6, 135.4, 133.9, 133.5, 133.4, 133.4, 131.7, 131.4, 129.4, 128.9, 128.6, 123.9, 122.7, 119.8, 119.2, 109.3, 93.0, 52.9, 30.5, 29.3, 29.2, 26.9. HPLC [gradient: H_2_O/MeCN, 10–100% of MeCN in 10 min]: 5.972 min. HRMS (ESI^–^) *m*/*z*: calcd for C_91_H_69_N_7_O_35_ 1817.36781; found 1817.36886.

#### Tetrapodal **32**

Following the general procedure for methyl ester deprotection, intermediate **29** (15 mg, 0.01 mmol, 1.00 equiv) and LiOH·H_2_O (7 mg, 0.16 mmol, 24.00 equiv) afforded compound **32** (11 mg, 76%) as an amorphous white solid. ^1^H NMR (500 MHz, DMSO-*d*_6_) δ: 11.02 (bs, 4H, NH-1*^i^*Trp), 8.27 (d, *J* = 8.2 Hz, 4H, NHCO), 8.04 (d, *J* = 8.1 Hz, 8H, Ar), 7.98 (d, *J* = 8.1 Hz, 8H, Ar), 7.74 (m, 20H, Ar), 7.13 (m, 8H, Ar), 4.61 (m, 4H, α-CHTrp), 3.33 (m, 20H, OCH_2_ and β-CH_2_Trp), 3.14 (m, 4H, β-CH_2_Trp), 2.21 (m, 8H, CH_2_). ^13^C NMR (400 MHz, DMSO-*d*_6_) δ: 173.2, 170.1, 167.2, 143.1, 136.6, 135.9, 133.5, 129.7, 129.4, 129.2, 129.1, 129.0, 128.7, 124.4, 122.7, 119.7, 119.3, 109.7, 68.8, 67.4, 66.9, 53.0, 44.9, 35.7, 27.4. HPLC [gradient: H_2_O/MeCN, 10–100% of MeCN in 10 min]: 7.295 min. HRMS (ESI^–^) *m*/*z*: calcd for C_117_H_98_N_8_O_32_ 2126.62871; found 2126.62712.

#### Tetrapodal **33**

Following the general procedure for methyl ester deprotection, intermediate **30** (20.0 mg, 0.01 mmol, 1.00 equiv) and LiOH·H_2_O (12 mg, 0.29 mmol, 40.00 equiv) afforded **33** (17.0 mg, 95%) as an amorphous white solid. ^1^H NMR (400 MHz, DMSO-*d*_6_) δ: 11.23 (s, 4H, NH-1*^i^*Trp), 8.47 (dt, *J* = 6.3, 1.6 Hz, 8H, Ar), 8.30 (dd, *J* = 13.5, 1.6 Hz, 16H, Ar), 8.18 (d, *J* = 7.9 Hz, 4H, NHCO), 7.74 (m, 4H, Ar), 7.15 (d, *J* = 6.2 Hz, 8H, Ar), 4.51 (m, 4H, α-CHTrp), 3.35 (m, 20H, OCH_2_ and β-CH_2_Trp), 3.15 (m, 4H, β-CH_2_Trp), 2.19 (m, 8H, CH_2_). ^13^C NMR (126 MHz, DMSO-*d*_6_) δ: 172.9, 170.0, 166.7, 166.7, 139.5, 135.6, 133.8, 133.4, 131.8, 129.4, 128.9, 128.6, 123.9, 122.6, 119.7, 119.2, 109.2, 68.9, 67.1, 52.8, 44.8, 35.6, 27.0. HPLC [gradient: H_2_O/MeCN, 10–100% of MeCN in 10 min]: 5.827 min. HRMS (ESI^–^) *m*/*z*: calcd for C_125_H_96_N_8_O_48_ 2476.5317; found 2476.5306.

#### Hexamethyl 5,5′,5″-(3-(2-((*tert*-Butoxycarbonyl)amino)-3-methoxy-3-oxopropyl)-1*H*-indole-2,5,7-triyl)triisophthalate (**35**)

Commercially available *N*-Boc-l-tryptophan methyl ester **1** (200 mg, 0.63 mmol, 1.00 equiv), (1,5-cyclooctadiene) (methoxy)iridium(I) dimer ([Ir(cod)OMe]_2_) (37.5 mg, 0.056 mmol, 9 mol %), bis(pinacolato)diboron (1.12 g, 4.40 mmol, 7.00 equiv), and 3,4,7,8-tetramethyl-1,10-phenantroline (Me_4_Phen) (26.7 mg, 0.11 mmol, 18 mol %) were sealed in a dry reaction vial (microwave reactor vessel can also be used) equipped with a magnetic stirring bar under an argon atmosphere, and anhydrous tetrahydrofuran (5 mL) was added. The resulting red solution was stirred at 85 °C for 12 h. After cooling to room temperature and removal of volatiles under reduced pressure, the subsequent brown residue (compound **34**) was treated with tris(dibenzylideneacetone)dipalladium (Pd_2_(dba)_3_) (30 mg, 3.20 mmol, 5 mol %), SPhos (27 mg, 0.07 mmol, 10 mol %), tribasic potassium phosphate (686 mg, 3.20 mmol, 5.00 equiv), and methyl dimethyl 5-bromoisophthalate (635 mg, 2.36 mmol, 3.60 equiv). The tube was sealed and placed under an argon atmosphere. Then, anhydrous toluene (4 mL) was added via a syringe. The reaction mixture was stirred at 80 °C for 12 h and worked up as described in the general C7 arylation procedure. The resulting brown residue was purified by CCTLC using dichloromethane/methanol (20:1) as the eluent to afford compound **35** (91.2, 20%) as a brown amorphous solid. ^1^H NMR (400 MHz, CDCl_3_) δ: 8.75 (bs, 1H, NH-1*^i^*Trp), 8.67 (m, 2H, Ar), 8.60 (bs, 1H), 8.56 (d, *J* = 1.6 Hz, 2H, Ar), 8.51 (d, *J* = 1.4 Hz, 2H, Ar), 8.39 (d, *J* = 1.5 Hz, 2H, Ar), 7.93 (s, 1H, Ar), 7.52 (m, 1H, Ar), 4.96 (m, 1H, NHCO), 4.64 (m, 1H, α-CHTrp), 4.00 (s, 6H, OCH_3_), 3.94 (m, 12H, OCH_3_), 3.53 (m, 2H, β-CH_2_Trp), 3.43 (s, 3H, OCH_3_), 1.19 (s, 9H, CH_3_). ^13^C NMR (101 MHz, CDCl_3_) δ: 172.5, 166.5, 166.1, 165.8, 154.9, 142.9, 139.4, 135.8, 133.8, 133.7, 133.6, 132.6, 132.5, 131.8, 131.5, 131.3, 130.2, 130.1, 129.0, 124.3, 122.9, 118.5, 110.1, 79.8, 54.2, 52.7, 52.3, 28.0, 24.7. HPLC [gradient: MeCN/H_2_O, 50–100% of MeCN in 10 min]: 8.250 min. HRMS (ESI^+^) *m*/*z*: calcd for C_47_H_46_N_2_O_16_ 894.2847; found 894.2837.

#### 5,5′,5″-(3-(2-Amino-2-carboxyethyl)-1*H*-indole-2,5,7-triyl)triisophthalic Acid (**37**)

A cold (0 °C) solution of compound **35** (30 mg, 0.03 mmol) in dichloromethane (10 mL) was treated with TFA (0.5 mL) as described for **7** to afford intermediate **36** in the salt form that was used in the next step without purification. Compound **36** was treated with LiOH·H_2_O (19.7 mg, 0.47 mmol, 14 equiv) following the general procedure for methyl ester deprotection to afford compound **37** (18.7 mg, 76%) as an amorphous white solid. ^1^H NMR (500 MHz, DMSO-*d*_6_) δ: 11.51 (s, 1H, NH-1*^i^*Trp), 8.52 (t, *J* = 1.7 Hz, 1H, Ar), 8.49 (d, *J* = 1.6 Hz, 2H, Ar), 8.48 (q, *J* = 1.9 Hz, 1H, Ar), 8.42 (d, *J* = 1.6 Hz, 1H, Ar), 8.40 (d, *J* = 1.7 Hz, 2H, Ar), 8.31 (d, *J* = 1.7 Hz, 2H, Ar), 8.14–8.09 (m, 1H, Ar), 7.49 (d, *J* = 1.5 Hz, 1H, Ar), 3.81–3.71 (m, 1H, α-CHTrp), 3.57–3.47 (m, 1H, β-CH_2_Trp), 3.30–3.20 (m, 1H, β-CH_2_Trp). ^13^C NMR (126 MHz, DMSO-*d*_6_) δ: 170.4, 166.8, 166.7, 142.2, 139.0, 137.4, 134.2, 133.7, 133.5, 132.7, 132.1, 132.0, 131.8, 131.5, 130.8, 129.8, 129.3, 128.9, 128.0, 124.8, 121.8, 117.3, 108.6, 54.2, 26.2. HRMS (ESI^+^) *m*/*z*: calcd for C_35_H_25_N_2_O_14_Cl 732.09943; found 732.09918.

### Biological Methods

#### Antiviral Activity against HIV

The MT-4 cells used for the anti-HIV assays were a kind gift from Dr. L. Montagnier (formerly at the Pasteur Institute, Paris, France) and cultured in Roswell Park Memorial Institute (RPMI)-1640 medium (Invitrogen, Merelbeke, Belgium) supplemented with 10% fetal calf serum (FCS) (Hyclone, Perbio Science, Aalst, Belgium) and 1% l-glutamine (Invitrogen). The HIV-1 strain NL4-3 was obtained from the AIDS Research and Reference Reagent Program (Division of AIDS, NIAID, NIH) and cultured in MT-4 cells. The virus stock was stored at −80 °C.

The compounds were evaluated for their inhibitory activity against HIV-1 (NL4.3) and HIV-2 (ROD) infection in MT-4 cell cultures, as have been described in detail earlier.^64^ Briefly, MT-4 cells (50 μL, 1 × 10^6^ cells/mL) were preincubated for 30 min at 37 °C with the test compounds (100 μL) in a 96-well plate. Next, the cell-line adapted HIV strains (NL4.3 and ROD) were added according to the 50% tissue culture infectious dose (TCID50) of the viral stock. After 5 days, the cytopathic effect (CPE) was scored microscopically and the anti-HIV-1 activity (50% effective concentration, EC_50_) of each compound was calculated using a colorimetric method based on the in situ reduction of 3-(4,5-dimethylthiazol-2-yl)-5-(3-carboxymethoxyphenyl)-2-(4-sulfophenyl)-2*H*-tetrazolium (MTS)^65^ according to the manufacturer’s instructions (Promega, Leiden, The Netherlands). Assays are performed by adding a small amount of a solution that contains tetrazolium compound MTS and an electron-coupling reagent (phenazine ethosulfate; PES), directly to culture wells, incubating for 1–4 h and then recording the absorbance at 490 nm (A490) with a 96-well plate reader. The quantity of the formazan product, as measured by the amount of 490 nm absorbance, is directly proportional to the number of living cells in culture. Cytotoxicity in MT-4 cells was measured after 5 days using the MTS/PES method.^64,65^ Data are the mean ± SD of at least three independent experiments.

PBMCs from healthy donors (obtained from the Red Cross Blood Transfusion Center, Leuven, Belgium) were resuspended in Roswell Park Memorial Institute (RPMI) 1640 medium supplemented with 10% FBS and 2 mM l-glutamine. The next day, cells were stimulated with phytohemagglutinin (PHA) at 2 μg/mL (Sigma, Bornem, Belgium) for 3 days at 37 °C. The PHA-stimulated blasts were then seeded at 0.5 × 10^6^ cells per well into a 48-well plate containing various concentrations of the compound in cell culture medium containing 10% FCS and IL-2 (25 U/mL; R&D Systems Europe, Abingdon, U.K.). The virus stocks were added at a final dose of 250 pg p24 HIV-1 Ag. The cell supernatant was collected on day 10 and HIV-1 core Ag in the culture supernatant was analyzed by a specific p24 Ag enzyme-linked immunosorbent assay (ELISA) kit (PerkinElmer, Zaventem, Belgium). Mock-infected cell cultures exposed to the different drug concentrations were examined for cell viability by the MTS/PES method.

#### Surface Plasmon Resonance (SPR) Analysis (HIV)

The SPR technique was used to determine the binding of **33** (**AL-518**) and **AL-471** (as positive control) to gp120, which was immobilized on a CM5 chip in a Biacore T200 instrument (GE Healthcare, Uppsala, Sweden). gp120 immobilization was performed using a standard amine coupling procedure. Briefly, the surface was activated using a 7 min injection of 1-ethyl-3-(3-dimethylaminopropyl)carbodiimide (EDC)/*N*-hydroxysuccinimide (NHS) 1:1. Following this, recombinant HIV-1 IIIB glycoprotein gp120 (ImmunoDx, Woburn, Massachusetts), diluted in pH 4 acetate immobilization buffer to 10 μg/mL, was injected on the surface for 420 s, resulting in a response of ±1400 RU. Afterward, the surface was deactivated by injecting 1.0 M ethanolamine–HCl pH 8.5 for 7 min. Finally, the surface was regenerated with 12 s injections of 50 mM NaOH and HCl pH 1.5 at 30 μL/min to remove any unbound ligand. All injections were performed with an injection speed of 5 μL/min excluding the surface regeneration step (30 μL/min). Interaction studies were performed at 25 °C in HBS-P+ (10 mM *N*-(2-hydroxyethyl)piperazine-*N*′-ethanesulfonic acid (HEPES), 150 mM NaCl, 0.05% surfactant P20; pH 7.4) supplemented with 5% DMSO. **AL-471** and **33** (**AL-518**) were diluted in twofold dilution steps with concentrations ranging from 0.1 to 12.5 μM. The analytes were injected using multiple cycle kinetics for 2 min at a flow rate of 30 μL/min, and the dissociation was measured for 4 min. A 12 s injection of 50 mM NaOH was used to regenerate the surface. Several buffer blanks were included for double referencing. Apparent binding kinetics (*K*_D_, *k*_a_, *k*_d_) were derived after fitting the experimental data to the 1:1 Langmuir binding model in Biacore T200 Evaluation Software 3.1. Solvent correction was applied to compensate for the DMSO bulk effects. The experiments were performed in quadruplicates.

Additional SPR experiments were now performed to analyze inhibition of viral gp120 binding to the host cell’s CD4 receptor by **33** (**AL-518**) and the prototype **AL-471**. For this experiment, human histidine-tagged CD4 (Life Technologies Europe, Merelbeke, Belgium) was immobilized on a nitrilotriacetic acid (NTA) chip. Immobilization was performed using a standard nickel chelation procedure. First, two channels of the chip (reference and analysis channel) were activated with 1 min injection of 0.5 mM NiCl_2_ at a flow rate of 10 μL/min. Afterward, 2 μg/mL His-CD4 was immobilized onto the second channel (analysis channel) by a 60 s injection at a flow rate of 10 μL/min. Next, a mixture of 25 nM gp120 with either **33** (**AL-518**) or **AL-471** at varying concentrations was injected for 2 min over both channels. The compounds mixed with gp120 were used in a concentration range between 10 000 and 10 nM using fourfold dilution steps. Finally, the chip surface was regenerated using three 1 min injections of 350 mM ethylenediaminetetraacetic acid (EDTA) at a flow rate of 30 μL/min. Binding percentages were calculated using the maximum response at the end of each injection and comparing it to the pure 25 nM gp120 injection. The experiments were performed in triplicate.

#### Antiviral Activity against EV-A71

The EV-A71 BrCr laboratory-adapted strain and clinical isolates representative of B genogroup (B2 sub-genogroup, 11316; B5 sub-genogroup, TW/70902/08) and C genogroup (C2 sub-genogroup, H08300 461#812; C4 sub-genogroup, TW/1956/05) were used at a low multiplicity of infection (MOI) in a standardized cell-based antiviral assay. Briefly, rhabdomyosarcoma (RD) cells were seeded in a 96-well plate. The day after, a serial dilution of the compounds and the virus inoculum was added to the cells. The assay plates were incubated at 37 °C, 5% CO_2_ with virus inoculum and compounds until full virus-induced cell death was observed in the untreated, infected controls (3 days postinfection). Subsequently, the antiviral effect was quantified using a colorimetric readout with 3-(4,5-dimethylthiazol-2-yl)-5-(3-carboxymethoxyphenyl)-2-(4-sulfophenyl)-2*H*-tetrazolium/phenazine methosulfate (MTS/PMS method), and the concentration of the compound at which 50% inhibition of virus-induced cell death was observed (EC_50_) was calculated from the antiviral dose–response curves. A similar assay setup was used to determine the adverse effect of the compound on uninfected, treated cells for calculation of CC_50_ (concentration of the compound that reduces the overall cell health by 50% as determined by the MTS/PMS method). The selectivity index (SI) was calculated as the ratio of CC_50_ to EC_50_.

### Computational Methods

#### In Silico Model Building of Ligands and HIV-1 Env Glycoproteins

The molecular graphics program PyMOL^66^ was employed for molecular editing, visualization, and figure preparation. The structure of the HIV-1 envelope protein (Env, UniProt code Q2N0S6), consisting of a trimer of fully cleaved gp120 and gp41 subunits, was taken from the PG16-Env complex deposited in the Protein Data Bank with accession code 6ULC.^25^ Each protein fragment was conveniently “capped” by acetyl (ACE) and *N*-methyl amide (NME) groups at N- and C-termini, respectively. Disulfide bonds were explicitly defined for the following cysteine pairs: 54–74, 119–205, 126–196, 131–157, 218–247, 228–239, 296–331, 378–445, and 385–418 in gp120 and 598–604 in gp41. Replacing the standard ASN name for asparagine by the N-linked NLN residue in AMBER allowed attachment of a conserved glycan profile compatible with a “consensus glycosylation target”,^67^ namely, (i) Man_9_GlcNAc_2_ at glycosylation positions 156 and 332; Man_3_GlcNAc_2_ at glycosylation positions 88, 134, 138, 160, 197, and 276; Man_1_GlcNAc_2_ at 295, 301, 339, 363, 386, and 411 in gp120; and (ii) GlcNAc_2_ at 262 and 448 in gp120 and 611, 625, and 637 in gp41 (residue numbers are standardized using the reference HIV-1 strain, HXB2). The Carbohydrate and Glycoprotein Builders publicly available at the GLYCAM-Web server (https://dev.glycam.org/) were used for the generation of free Man_9_GlcNAc_2_-OMe and for interactive protein glycosylation, respectively. The AMBER-compatible GLYCAM06 force field^68^ was used for the carbohydrates.

For completion, the membrane-proximal external region and the transmembrane (TM) segments of gp41 (residues 664–683) were taken from PDB entry 6E8W(69) and embedded in a cholesterol-rich lipid bilayer using the CHARMM-GUI Membrane Builder pipeline.^70^ The outer layer consisted of 54 sphingomyelin (PSM, 18:1/16:0), 26 dipalmitoylphosphatidylcholine (DPPC, 16:0/16:0), and 90 cholesterol molecules; the inner layer was composed of 25 dipalmitoylphosphoserine (DPPS, 16:0/16:0) and 112 cholesterol molecules, interspersed with 44 units of the plasmalogen or ether lipid DPPE-E (16:0/16:0), in accordance with data from the HIV lipidome.^71^ The AMBER lipid17 and lipid17_ext force fields^72,73^ were used for lipids already in the database; consistent parameters and point charges were derived for PSM and DPPE-E fragments using antechamber in AMBER18. The central positioning of the TM segment with respect to the membrane normal was unbiasedly performed by the PPM utility implemented in the Orientations of Proteins in Membranes (OPM) database (http://opm.phar.umich.edu).^74^ About 100 000 TIP3P water molecules were then placed along the *Z*-axis on both sides of the lipid bilayer to solvate the protein as well as the lipid head groups. Finally, electroneutrality was achieved and the bulk ion concentration was set at 0.15 M by adding 462 potassium and 272 chloride ions.

#### Molecular Dynamics Simulations

For more efficient conformational sampling of **33** (**AL-518**) and identification of possible modes of interaction with the glycan shield of the HIV Env protein, the molecule was immersed in a truncated octahedral box of TIP3P water molecules, which also contained counterions and two molecules of Man_9_GlcNAc_2_-OMe. System coordinates were first relaxed by performing 25 000 steps of steepest descent followed by 100 000 steps of conjugate gradient energy minimization. The resulting configurations were then used as input for molecular dynamics (MD) simulations at 300 K and 1 atm using the AMBER18 implementation of the pmemd.cuda engine,^75^ essentially as described earlier for **AL-385**.^38^ A weak harmonic restraint of 1 kcal·mol^–1^·Å^–2^ was initially imposed on the Trp Cα atoms to promote water and counterion equilibration. Thereafter, trajectory snapshots over 300 ns of unrestrained MD simulations were saved every 0.5 ns for further analysis and movie preparation.

The MD simulations of the membrane-embedded glycosylated gp120:gp41 trimer and the complex of the external region with **33** (**AL-518**) were run for 400 ns essentially as described before for other membrane receptors^76,77^ and EV71 VP1,^38^ respectively.

## Supporting Information Available

^1^H NMR, ^13^C NMR, and MS spectra of the synthesized compounds together with HPLC of representative compounds and two-dimensional spectra of tripodal **14** and tetrapodal **21** and **32** (PDF)
Interactions between the isophthaloyl moieties attached to C2 and C7 positions of Trp in **33** (**AL-518**) and four molecules of a model Manα_1-2_Man disaccharide used as a simplified version of the oligomannose-type *N*-glycans present in gp120 and gp41 (Movie S1) (MPG)
Protein surface coverage provided by the highly mobile glycan “cloud” around gp120 and gp41 in a membrane-embedded HIV-1 Env protein (Movie S2) (MPG)
Clustering of solutions and alternative accommodations of **33** (**AL-518**) interacting with the glycans surrounding the V3 loop of gp120 throughout the unrestrained molecular dynamics simulations (200–400 ns postequilibration period) in the presence of explicit water molecules and counterions (Movie S3) (MPG)
Cartoon representation of an HIV-1 glycosylated gp120:gp41 dimer in complex with a two-domain fragment of the human CD4 receptor (Movie S4) (MPG)
Molecular formula strings of the novel synthesized compounds (CSV)

## Supplementary Material

jm1c00315_si_001.mpg

jm1c00315_si_002.mpg

jm1c00315_si_003.mpg

jm1c00315_si_005.csv

jm1c00315_si_006.mpg

## Dedication

^†^ This work is dedicated to Prof. Jan Balzarini (KU Leuven, Belgium), on the occasion of his retirement, in recognition of his constant encouragement and exemplary dedication to virology.

## Abbreviations

AIDS: acquired immune deficiency syndrome
bnAbs: broadly neutralizing antibodies
CCR5: chemokine receptor type 5
CXCR4: C-X-C α-chemokine receptor type 4
DIPEA: *N*,*N*-diisopropylethylamine
DMF: dimethylformamide
DS-5000: dextran sulfate-5000
EV71: enterovirus 71
EV-A71: enterovirus 71 genogroup A
FCS: fetal calf serum
HHA: *Hippeastrum* hybrid agglutinin
HATU: hexafluorophosphate azabenzotriazole tetramethyl uronium
HIV: human immunodeficiency virus
mAb: monoclonal antibody
MTS: 3-(4,5-dimethylthiazol-2-yl)-5-(3-carboxymethoxy-phenyl)-2-(4-sulfophenyl)-2*H*-tetrazolium
NTA: nitrilotriacetic acid
PBMCs: peripheral blood mononuclear cells
PES: phenazine ethosulfate
PHA: phytohemagglutinin
PRM-A: pradimicin A
SMR: Suzuki–Miyaura reaction
SPR: surface plasmon resonance
TFA: trifluoroacetic acid
Trp: tryptophan
