# Supplementary material for: Double Arylation of the Indole Side Chain of Tri- and Tetrapodal Tryptophan Derivatives Renders Highly Potent HIV-1 and EV-A71 Entry Inhibitors†
Source: J Med Chem. 2021 Jul 7;64(14):10027–46. doi: 10.1021/acs.jmedchem.1c00315 (PMC8389807; doi:10.1021/acs.jmedchem.1c00315)
Supplement: Supplementary file 4 — jm1c00315_si_004.pdf [file jm1c00315_si_004.pdf]

Electronic Supporting Information

Double Arylation of the Indole Side Chain of Tri- and Tetrapodal Tryptophan Derivatives Renders Highly Potent HIV-1 and EV-A71 Entry Inhibitors

Olaia Martí-Marí,[†] Belén Martínez-Gualda,[†#] Sofía de la Puente-Secades,[†&] Alberto Mills,[‡] Ernesto Quesada,[†] Rana Abdelnabi,[§] Liang Sun,[§] Arnaud Boonen,[§] Sam Noppen,[§] Johan Neyts,[§] Dominique Schols,[§] María-José Camarasa,[†] Federico Gago,[‡] and Ana San-Félix[†*]*

[†]Instituto de Química Médica (IQM-CSIC), E-28006 Madrid, Spain

[‡] Departamento de Ciencias Biomédicas y Unidad Asociada IQM-UAH, Universidad de Alcalá, E-28805 Alcalá de § Henares, Madrid, Spain

[§] University of Leuven, Department of Microbiology and Immunology, Rega Institute for Medical Research, Laboratory of Virology and Chemotherapy, B-3000 Leuven, Belgium

Present Addresses:

[#]Medicinal Chemistry, Rega Institute for Medical Research, KU Leuven, B-3000, Leuven, Belgium.

[&]Institute for Molecular Cardiovascular Research (IMCAR), RWTH Aachen University, University Hospital Aachen, 52074 Aachen, Germany.

Corresponding Authors

*Dr. Ana Rosa San Félix, Instituto de Química Médica (IQM-CSIC), c/ Juan de la Cierva 3 E-28006 Madrid (Spain)

Phone number: (+34) 912 587 689. e-mail: anarosa@iqm.csic.es

*Prof. Federico Gago, Área de Farmacología, Departamento de Ciencias Biomédicas, Universidad de Alcalá, E-28805 Alcalá de Henares, Madrid (Spain) Phone number: (+34) 918 854 514. e-mail: federico.gago@uah.es

Table of Contents

1. CHEMISTRY

1.1. ^{1}HNMR, ^{13}CNMR and MS spectra of synthesized compounds

1.2. HPLC chromatograms of the final compounds

*1.3. Two-dimensional spectra of trimer **14** and tetramers **21** and **32***

2. ANTIBODY STUDIES

2.1. *Inhibition of gp120 binding of mAb clone 447-52D by **AL-518** and **AL-471**.*

2.2. *Inhibition of gp120 binding of mAb clone 447-52D by **AL-518** and **AL-471***

3. COMPUTER-ASSISTED MOLECULAR MODELING

3.1. *Supplementary Movie 1*

3.2. *Supplementary Movie 2*

3.3. *Supplementary Movie 3*

3.4. *Figure S1 and Supplementary Movie 4*

1.1. ^{1}HNMR and ^{13}CNMR of the synthesized compounds

Compound **4**

^{1}H NMR

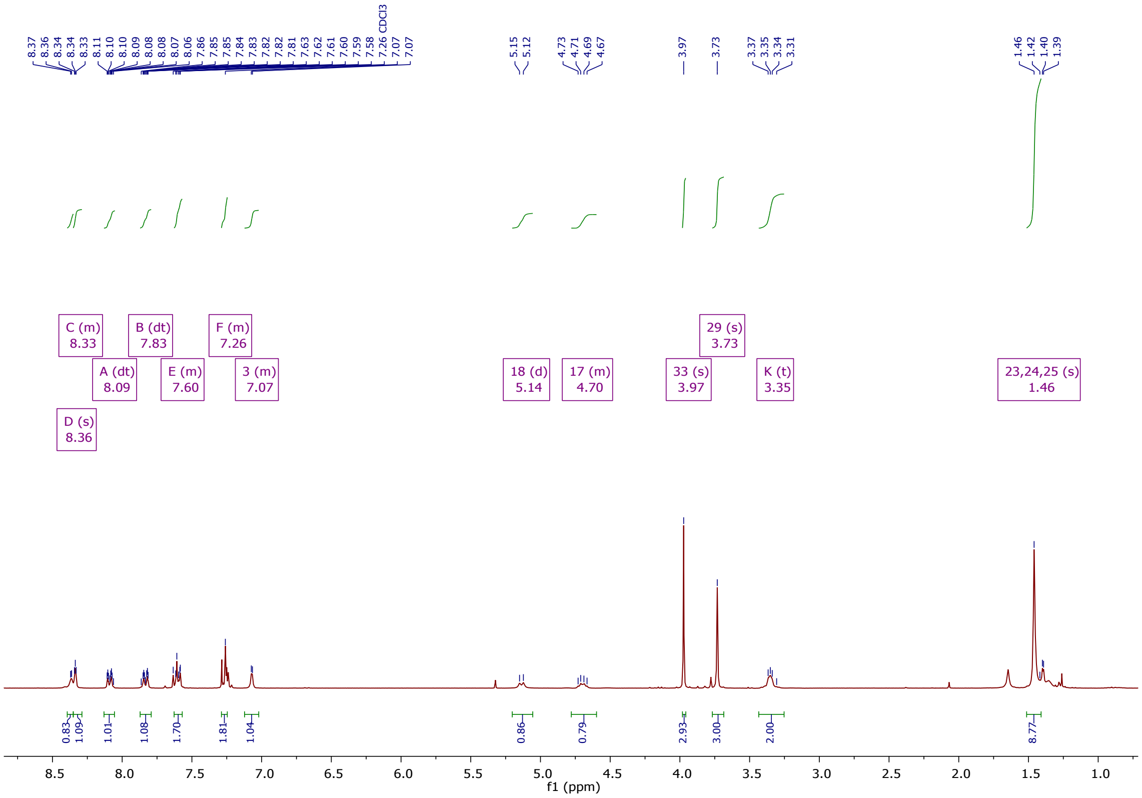

Compound **5**

^{1}H NMR

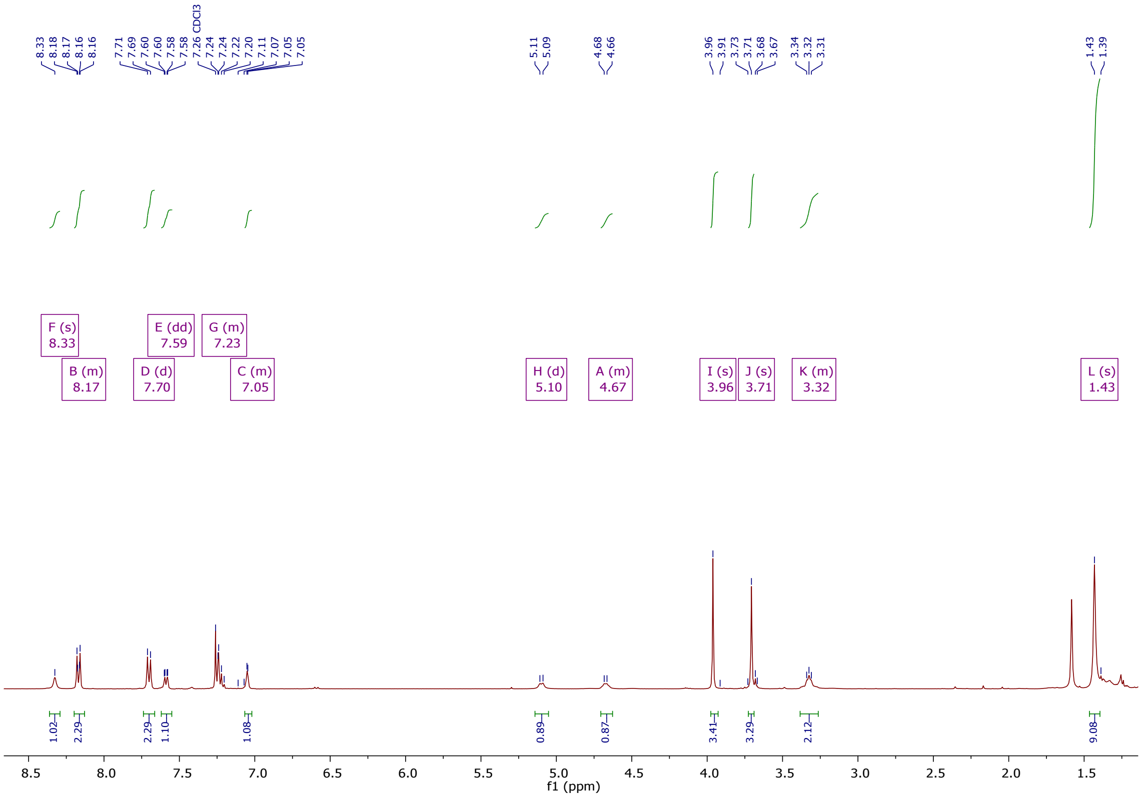

Compound **6**
^{1}H NMR

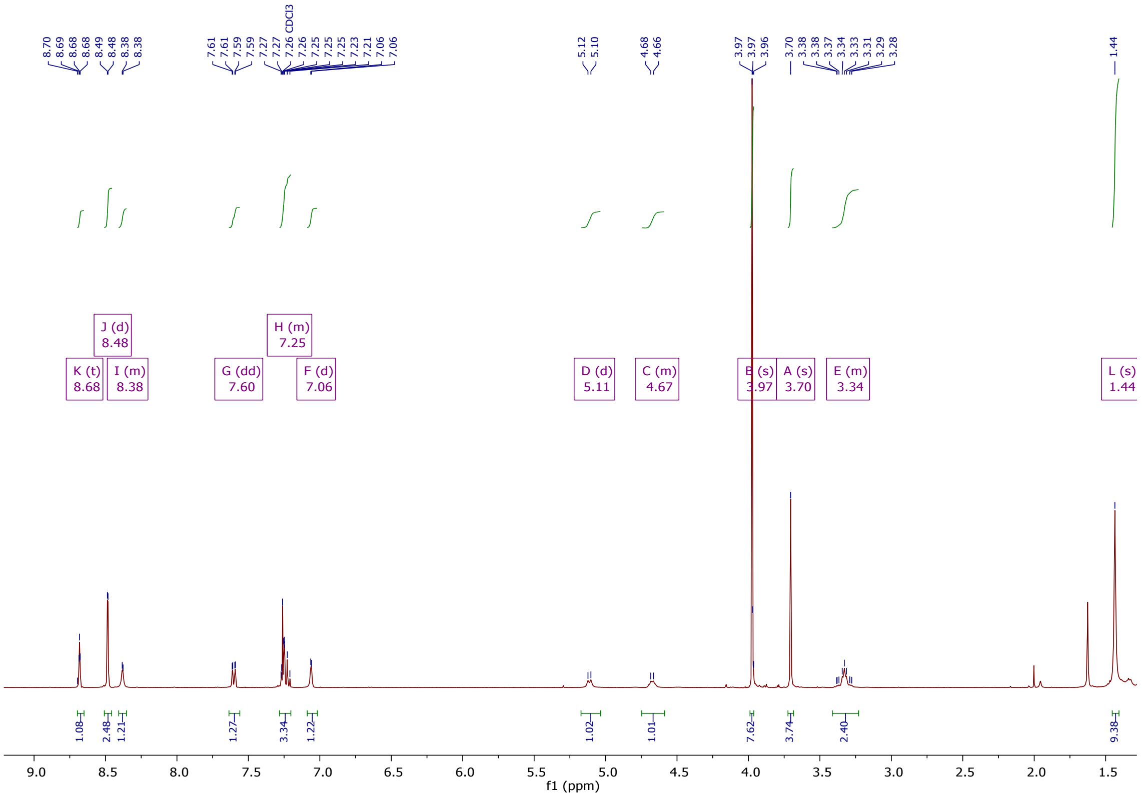

Compound **7**
^{1}H NMR

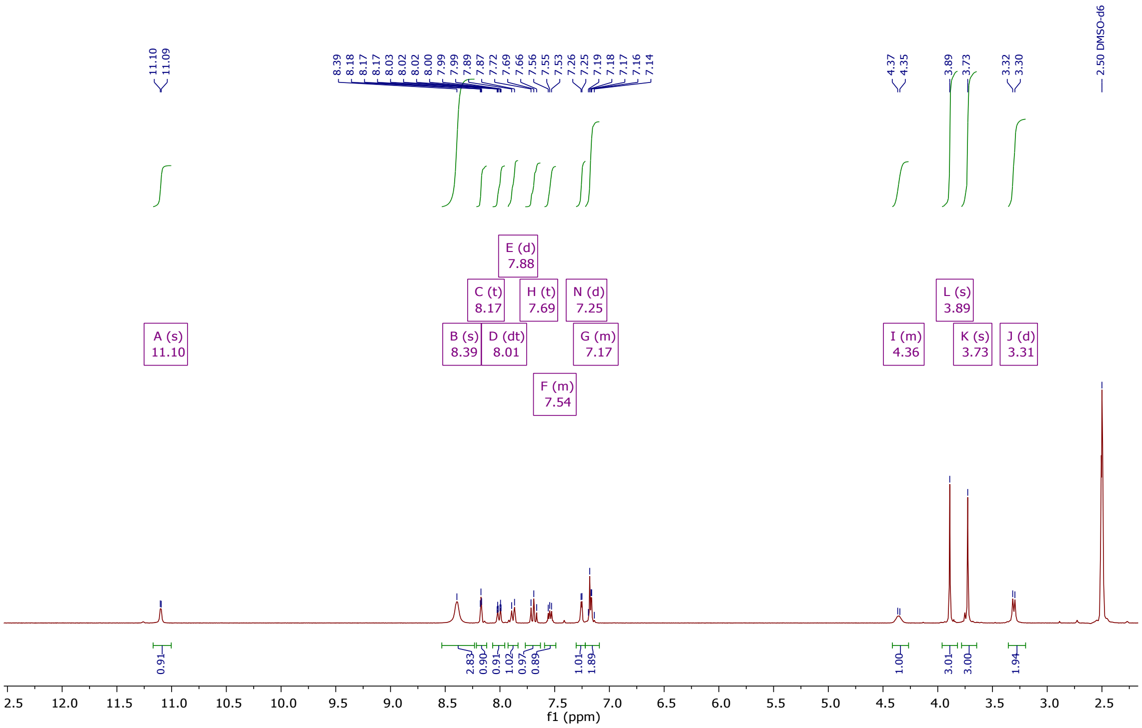

11.10
11.09
8.39
8.18
8.17
8.17
8.03
8.02
8.02
8.00
7.99
7.99
7.89
7.87
7.72
7.69
7.66
7.56
7.55
7.53
7.26
7.25
7.19
7.18
7.17
7.16
7.14
4.37
4.35
3.89
3.73
3.32
3.30
2.50 DMSO-d6
E (d) 7.88
C (t) 8.17
H (t) 7.69
N (d) 7.25
L (s) 3.89
A (s) 11.10
B (s) 8.39
D (dt) 8.01
G (m) 7.17
I (m) 4.36
K (s) 3.73
J (d) 3.31
F (m) 7.54
0.91
2.83
0.90
0.91
1.02
0.97
0.89
1.01
1.89
1.00
3.01
3.00
1.94
f1 (ppm)

Compound **8**

^{1}H NMR

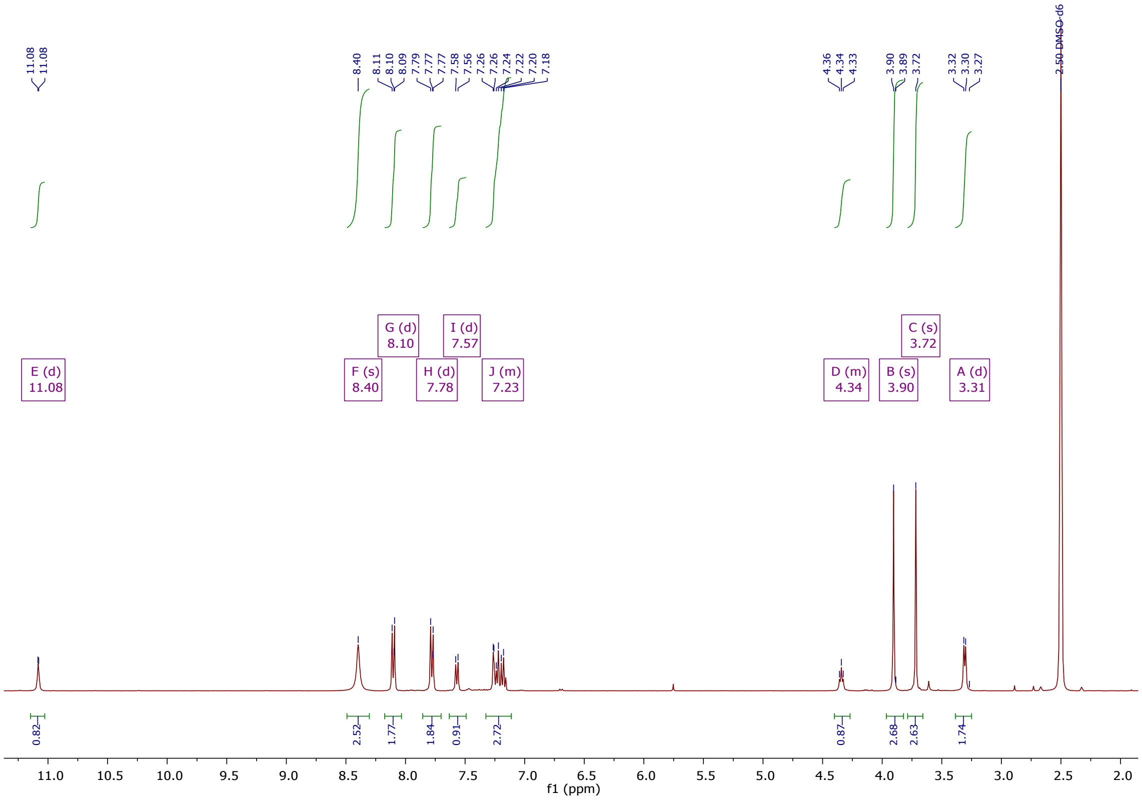

Compound **9**

^{1}H NMR

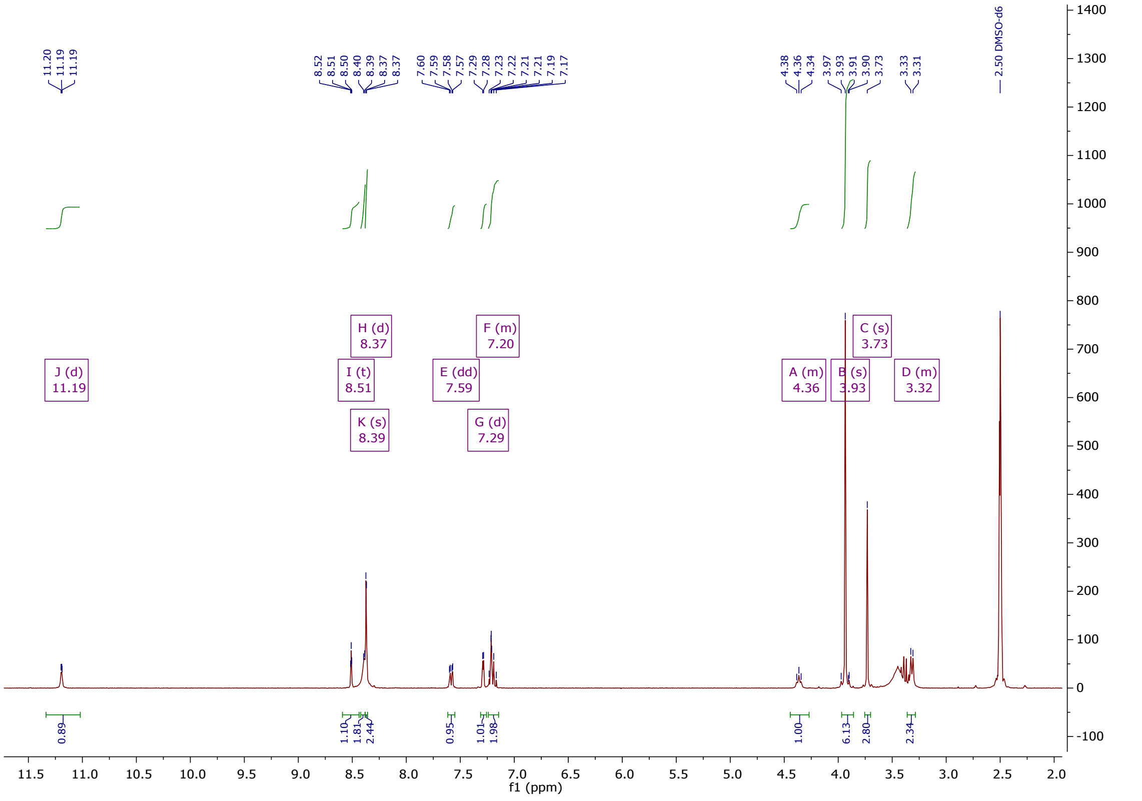

Trimer **11**

^{1}H NMR

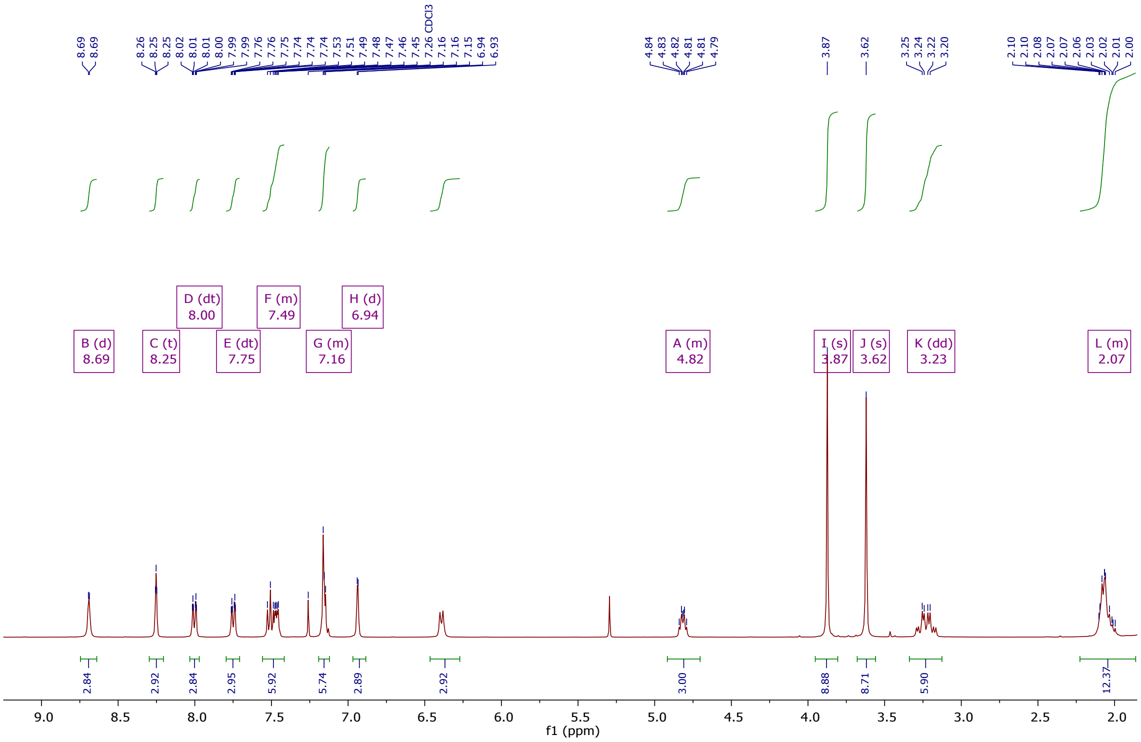

Trimer **12**

^{1}H NMR

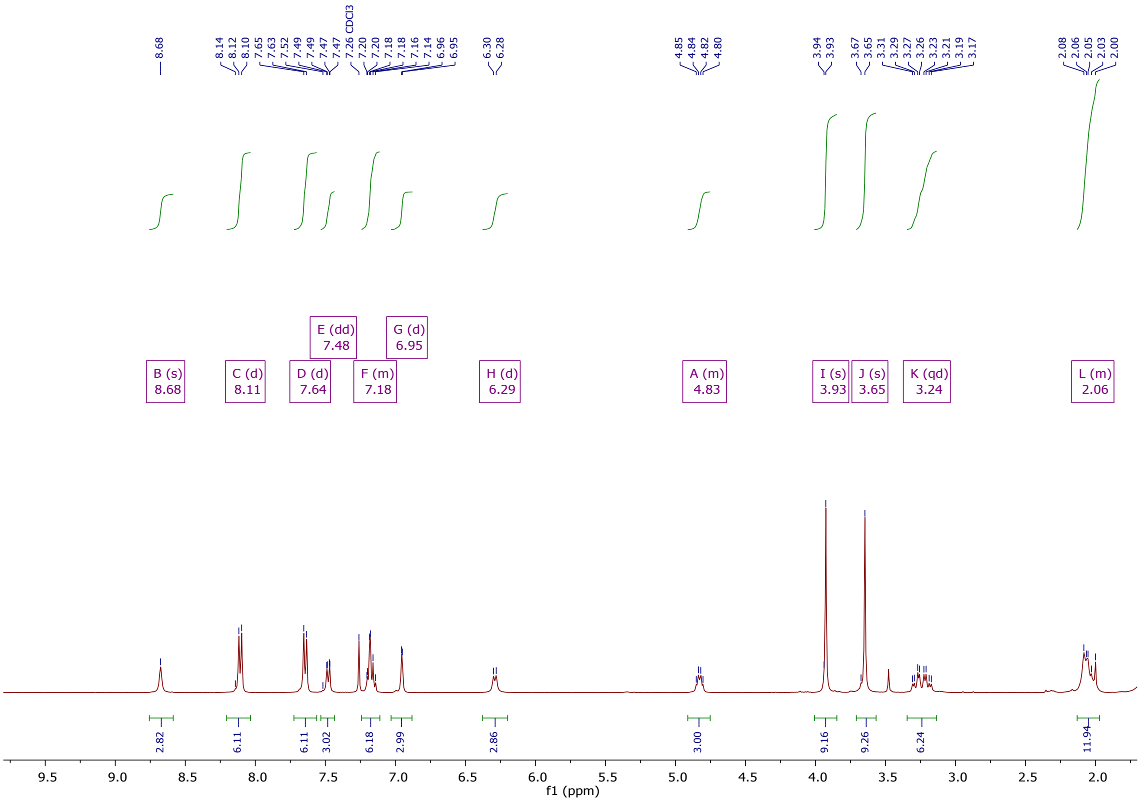

Trimer **13**

^{1}H NMR

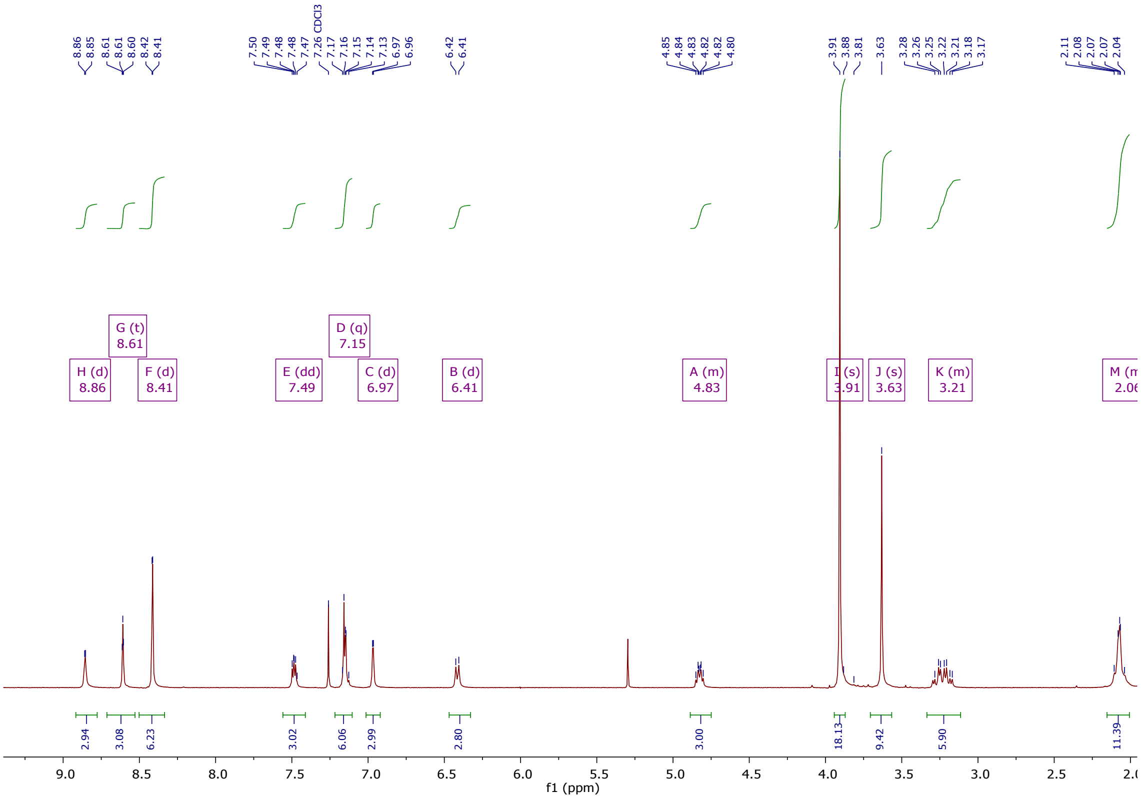

Trimer **14**

^{1}H NMR

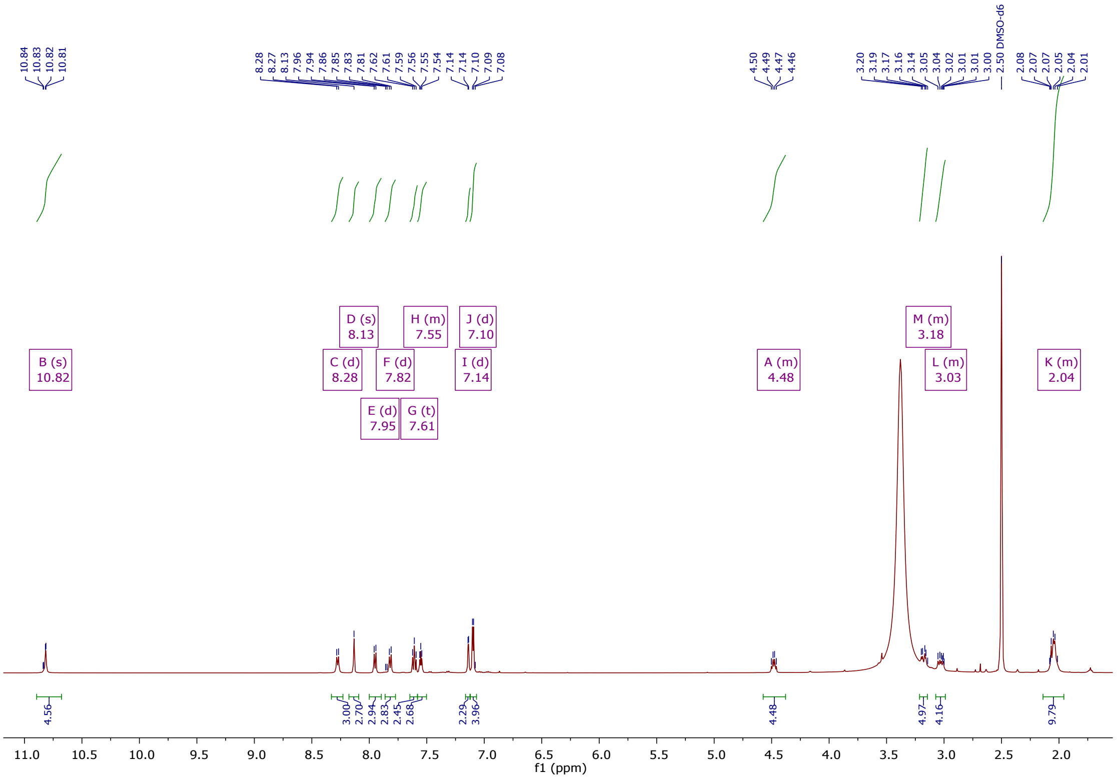

^{13}C NMR

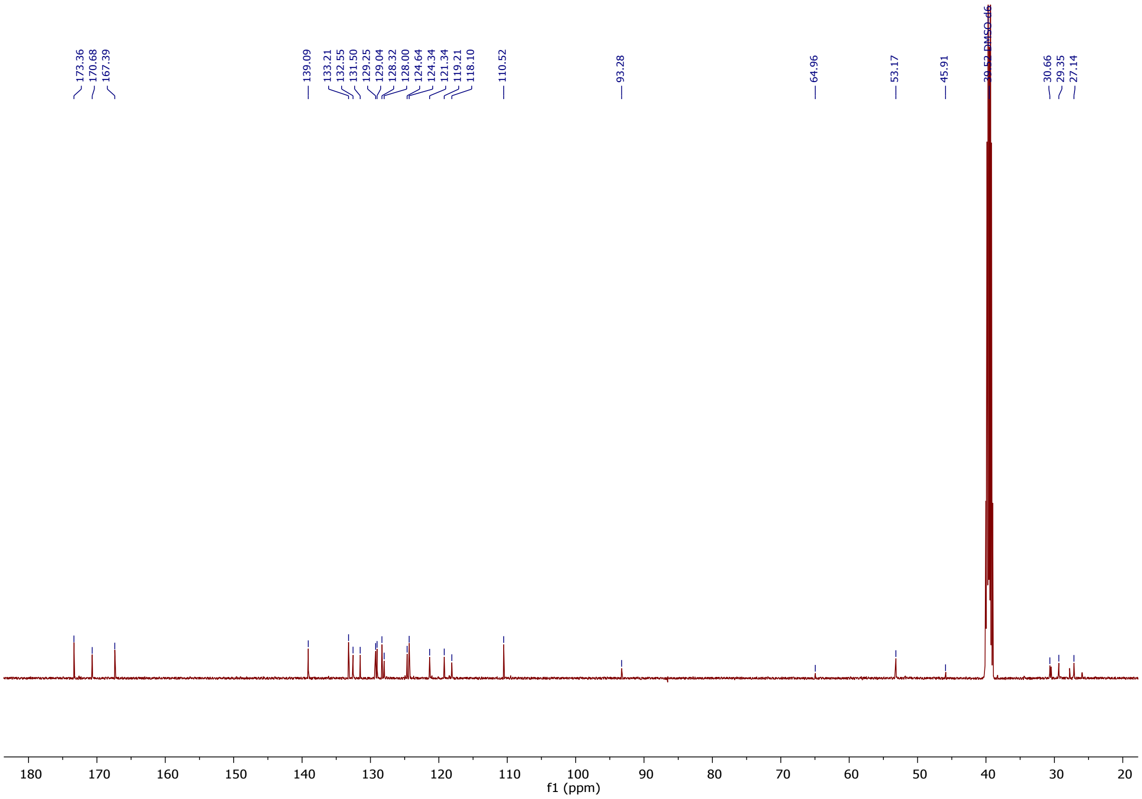

MS Spectrum

Compound Table

Compound Label	RT	Mass	Abund	Formula	Tgt Mass	Diff (ppm)
Cpd 1: C64 H57 N7 O17	0.464	1195.38089	5065	C64 H57 N7 O17	1195.38109	-0.17

Compound Label	RT	Algorithm	Mass
Cpd 1: C64 H57 N7 O17	0.464	Find By Formula	1195.38089

MS Zoomed Spectrum

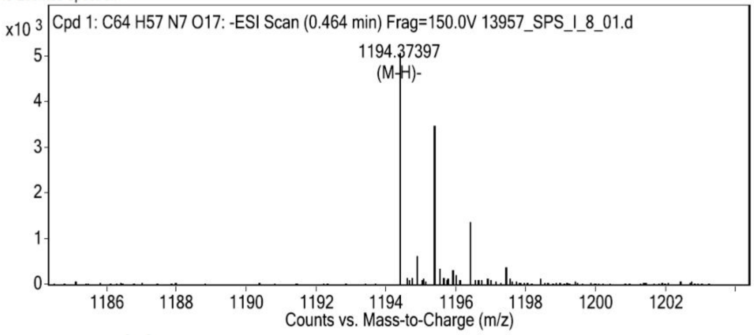

Trimer **15**

^{1}H NMR

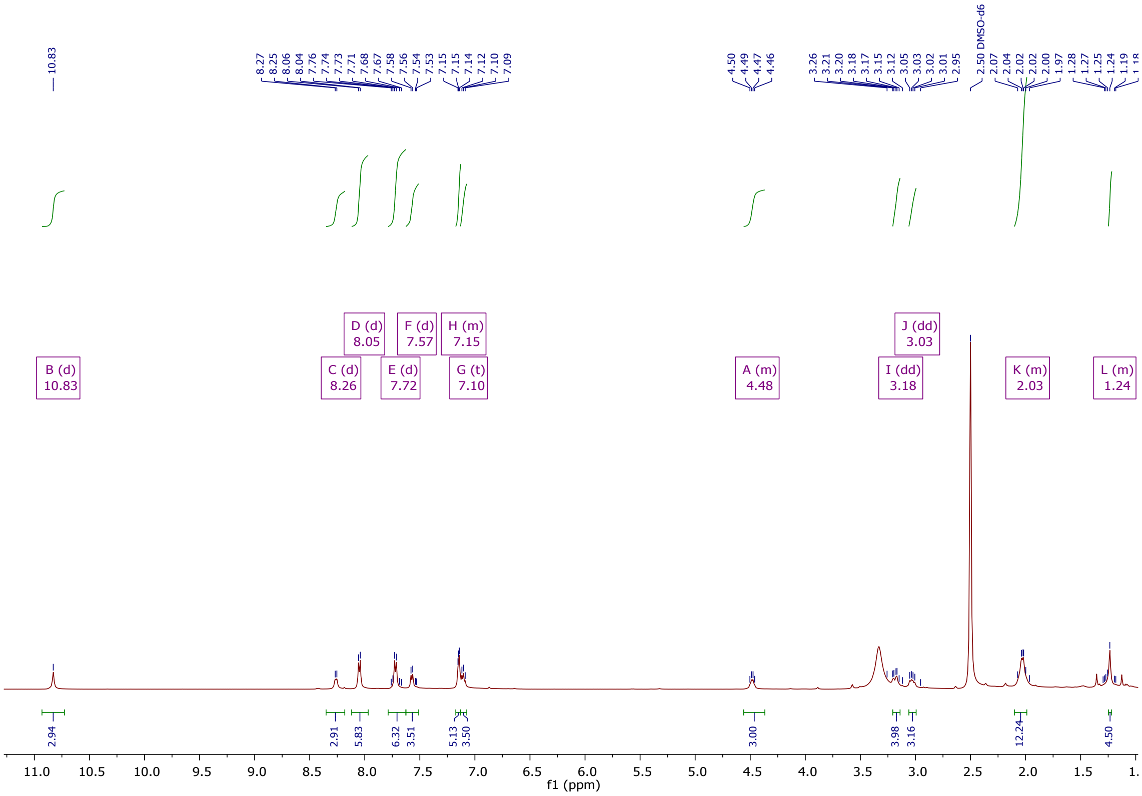

^{13}C NMR

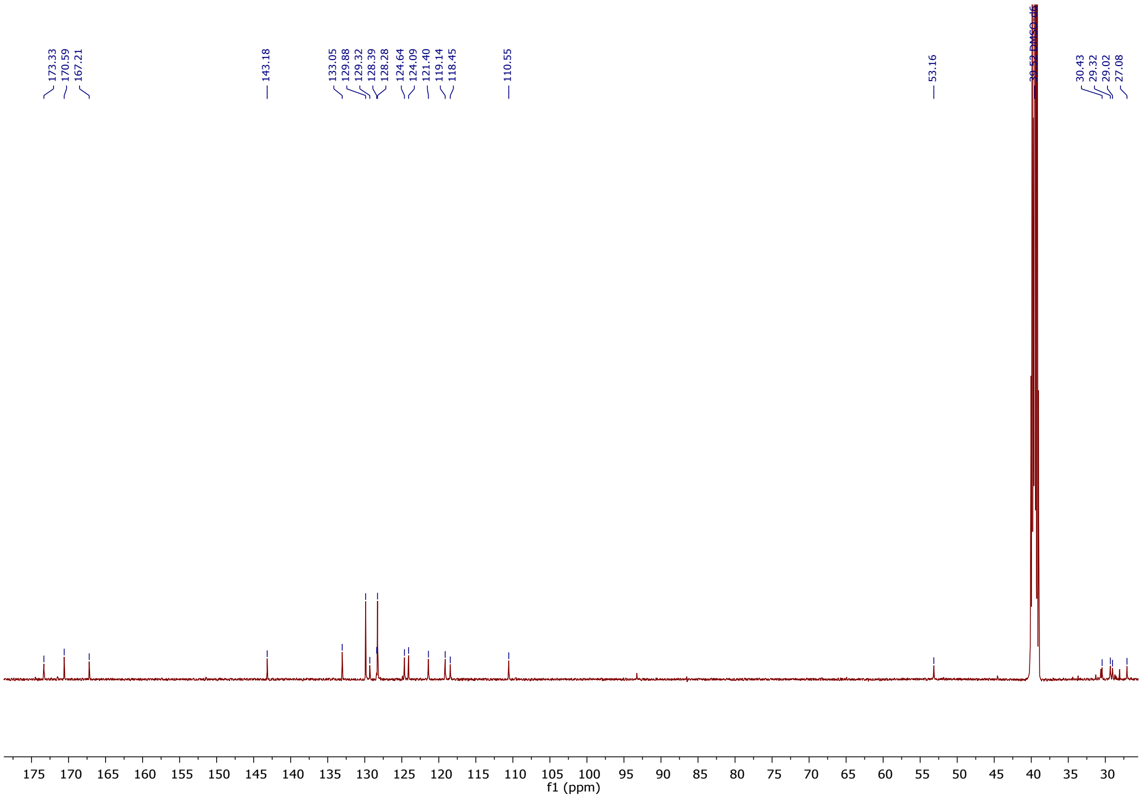

MS Spectrum

Compound Table

Compound Label	RT	Mass	Abund	Formula	Tgt Mass	Diff (ppm)
Cpd 1: C64 H57 N7 O17	0.535	1195.38042	10456	C64 H57 N7 O17	1195.38109	-0.56

Compound Label	RT	Algorithm	Mass
Cpd 1: C64 H57 N7 O17	0.535	Find By Formula	1195.38042

MS Zoomed Spectrum

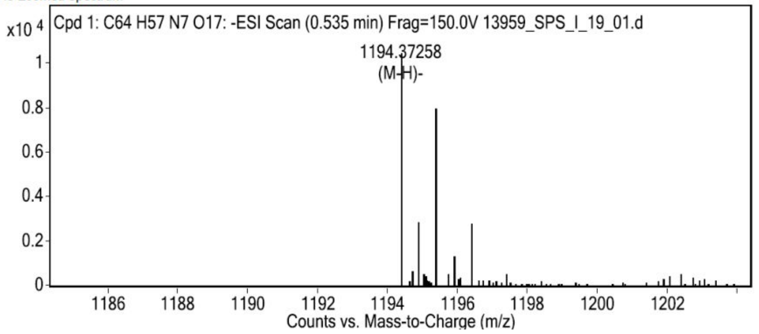

Trimer **16**

^{1}H NMR

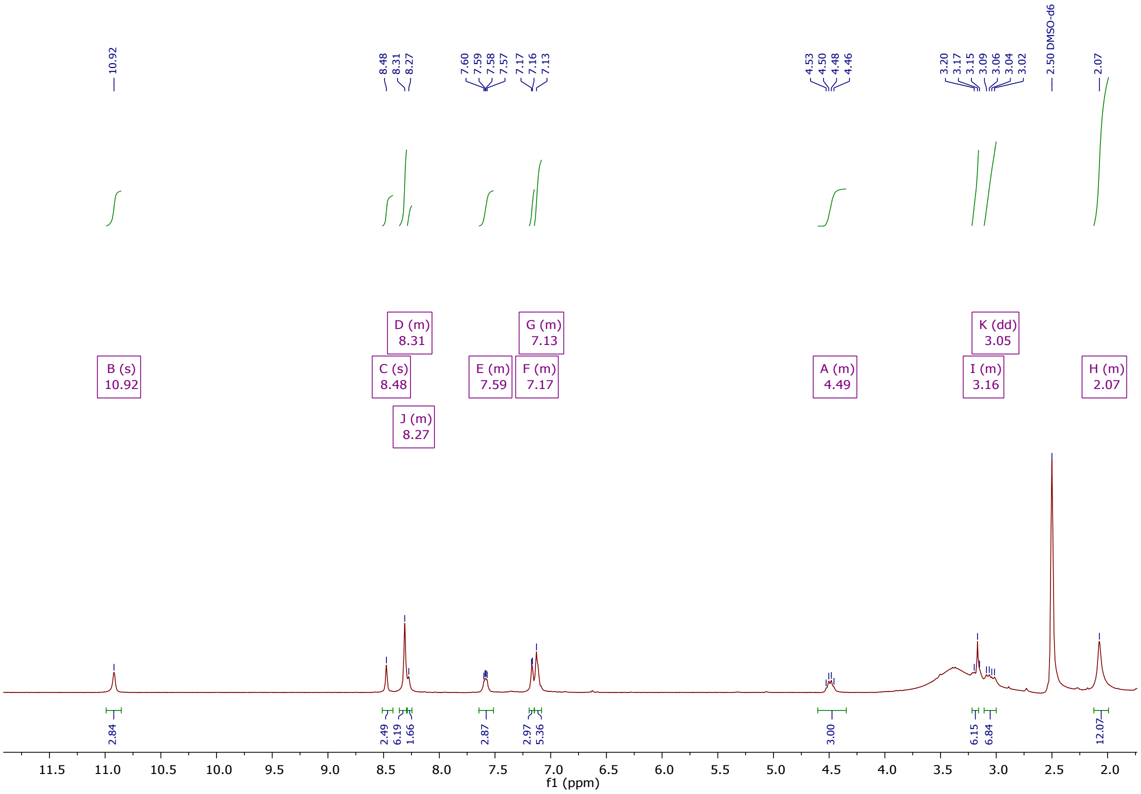

^{13}C NMR

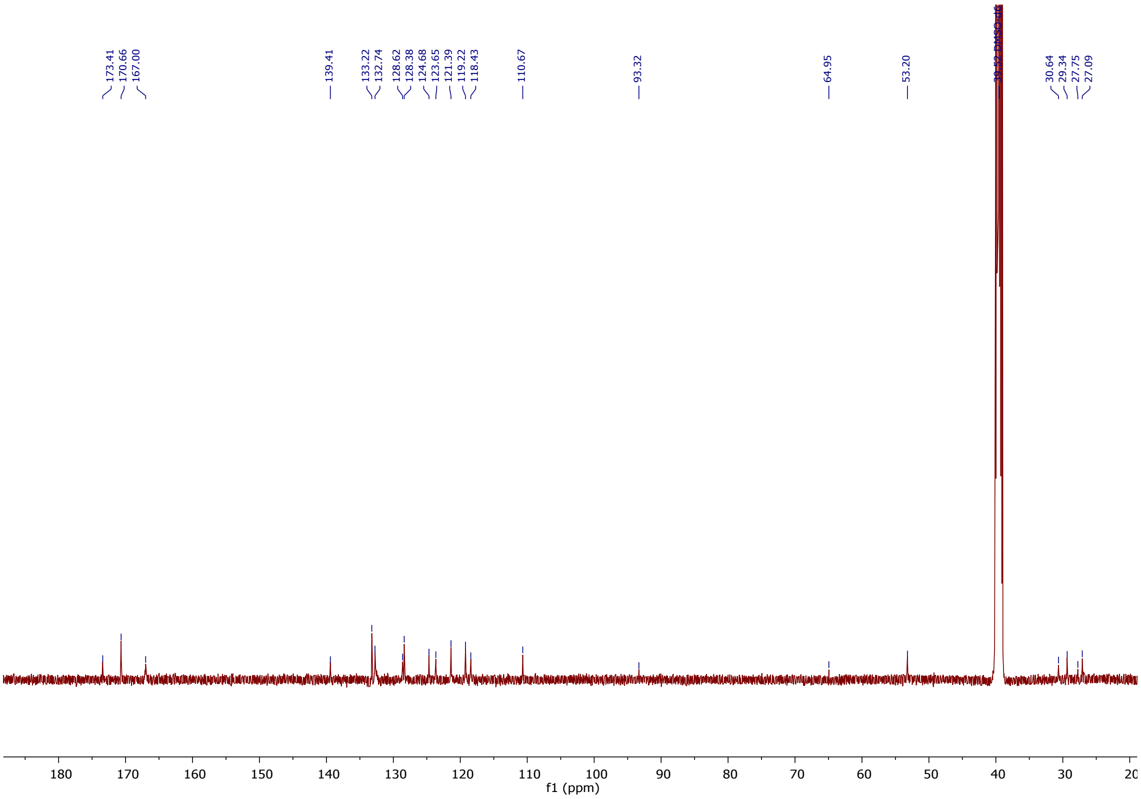

MS spectrum

Compound Table

Compound Label	RT	Mass	Abund	Formula	Tgt Mass	Diff (ppm)
Cpd 1: C67 H57 N7 O23	0.452	1327.35014	4542	C67 H57 N7 O23	1327.35058	-0.33

Compound Label	RT	Algorithm	Mass
Cpd 1: C67 H57 N7 O23	0.452	Find By Formula	1327.35014

MS Zoomed Spectrum

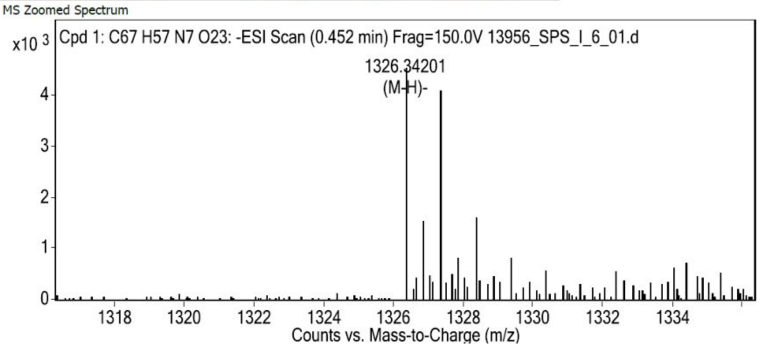

Tetramer **18**

^{1}H NMR

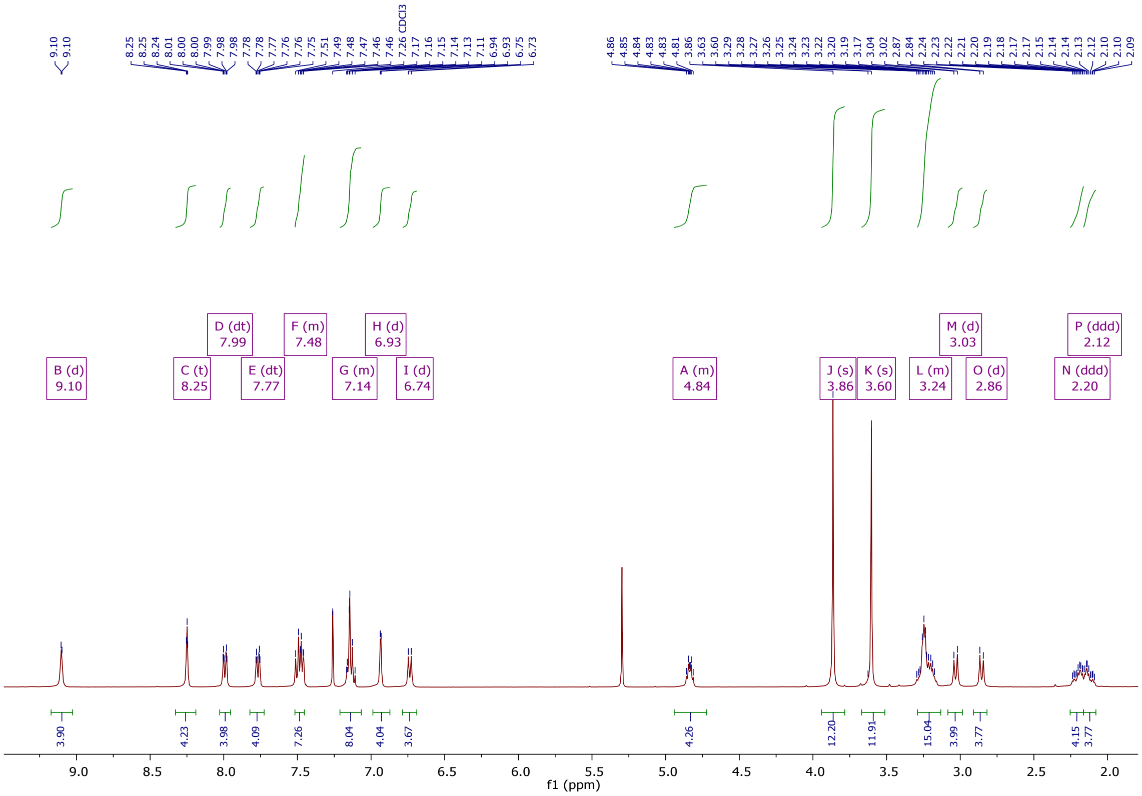

Tetramer **19**

^{1}H NMR

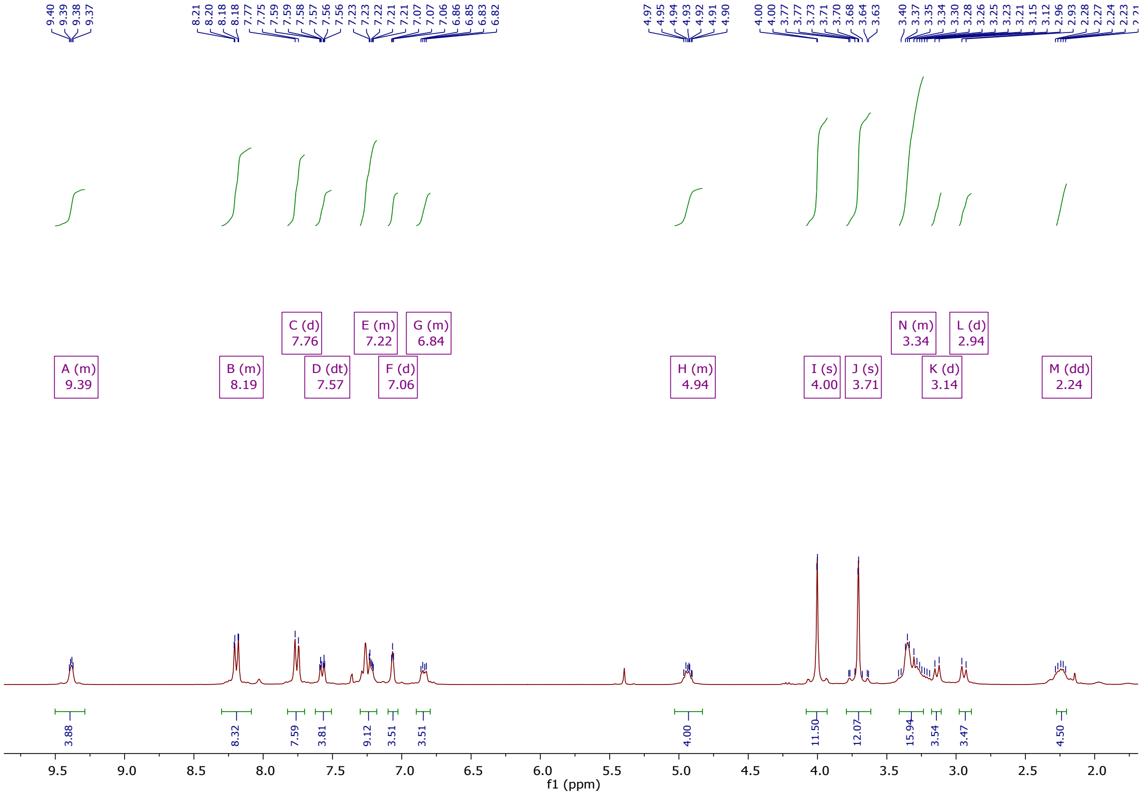

Tetramer **20**

^{1}H NMR

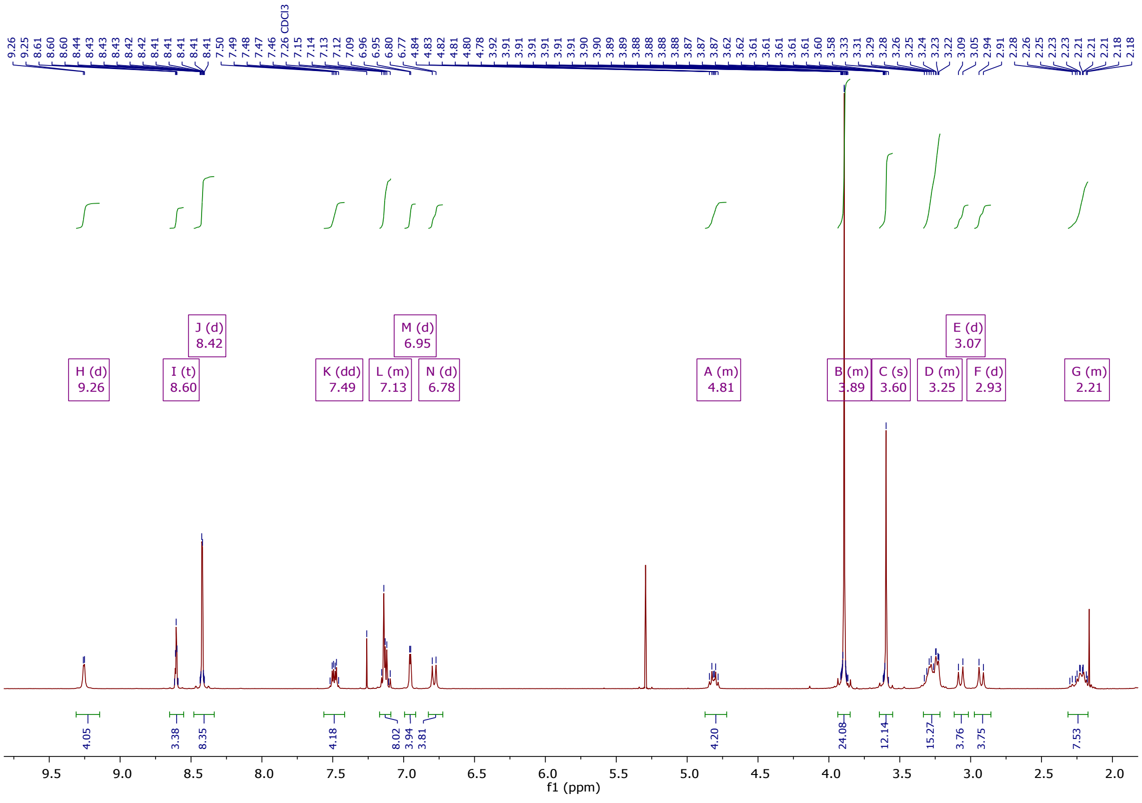

Tetramer **21**

^{1}H NMR

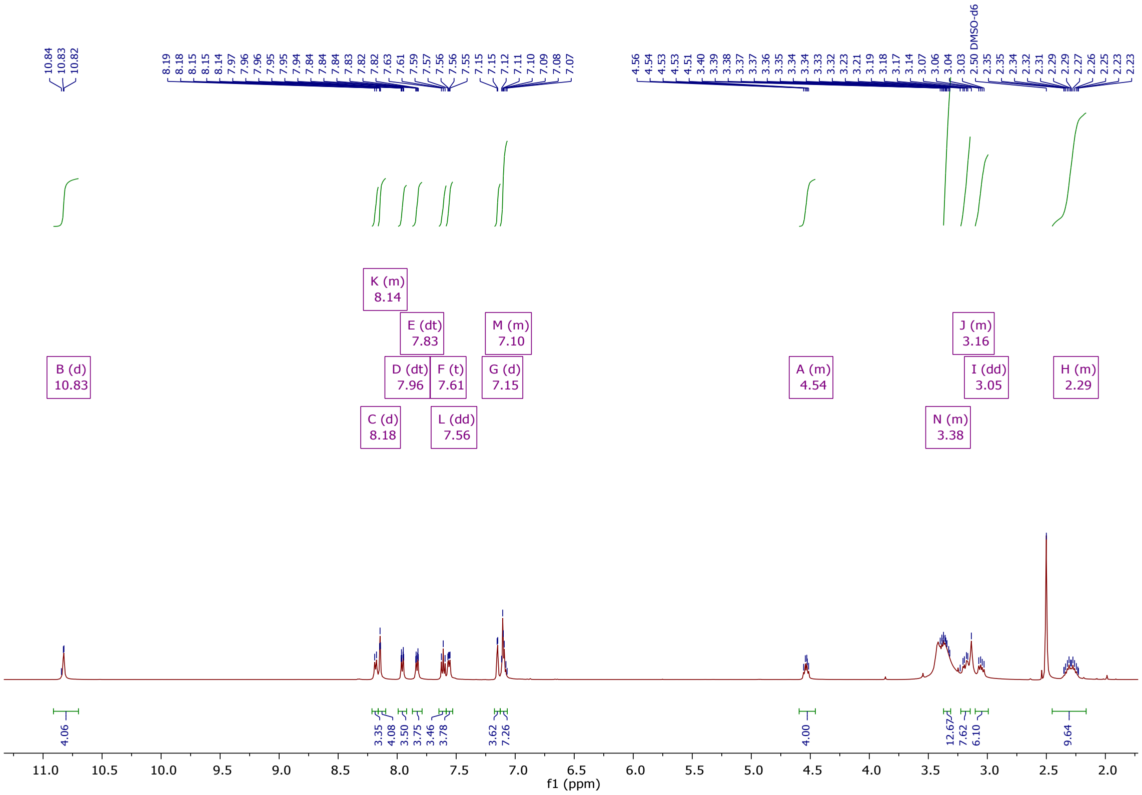

^{13}C NMR

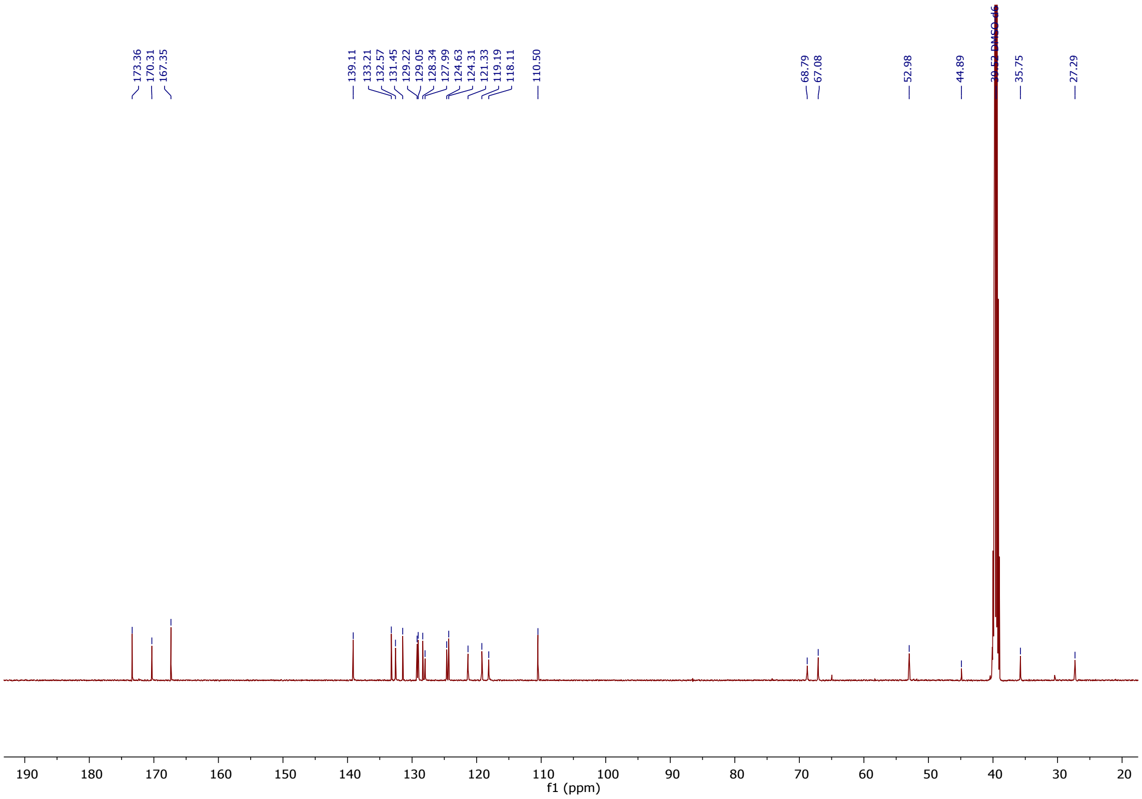

MS spectrum

Compound Table

Compound Label	RT	Mass	Abund	Formula	Tgt Mass	Diff (ppm)
Cpd 1: C89 H84 N8 O24	0.37	1648.55573	3038	C89 H84 N8 O24	1648.55985	-2.49

Compound Label	RT	Algorithm	Mass
Cpd 1: C89 H84 N8 O24	0.37	Find By Formula	1648.55573

MS Zoomed Spectrum

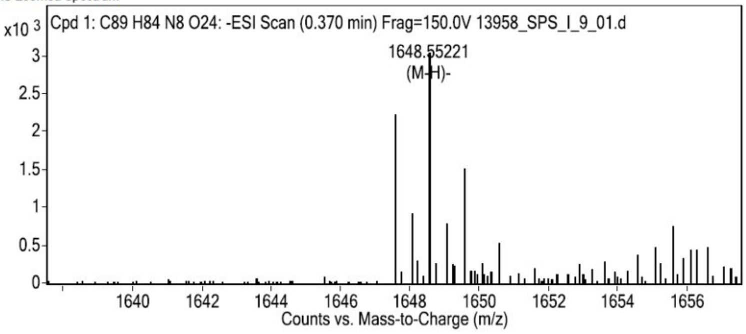

Tetramer 22

^{1}H NMR

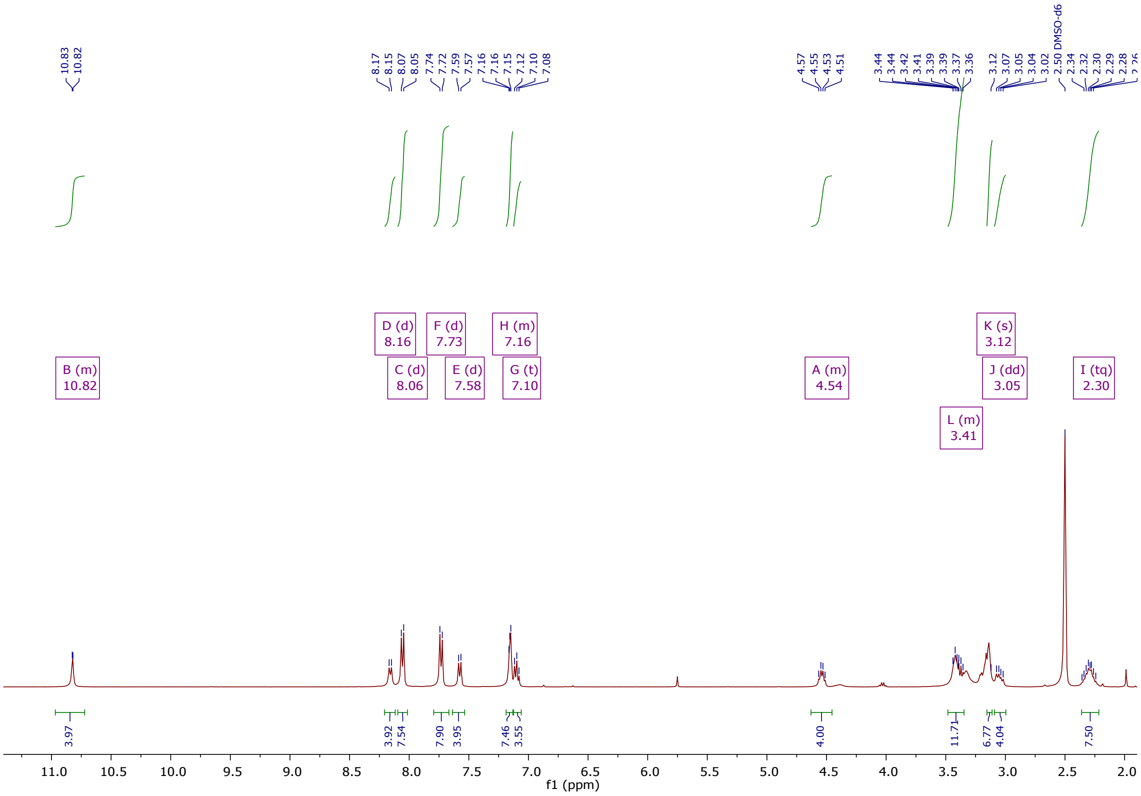

^{13}C NMR

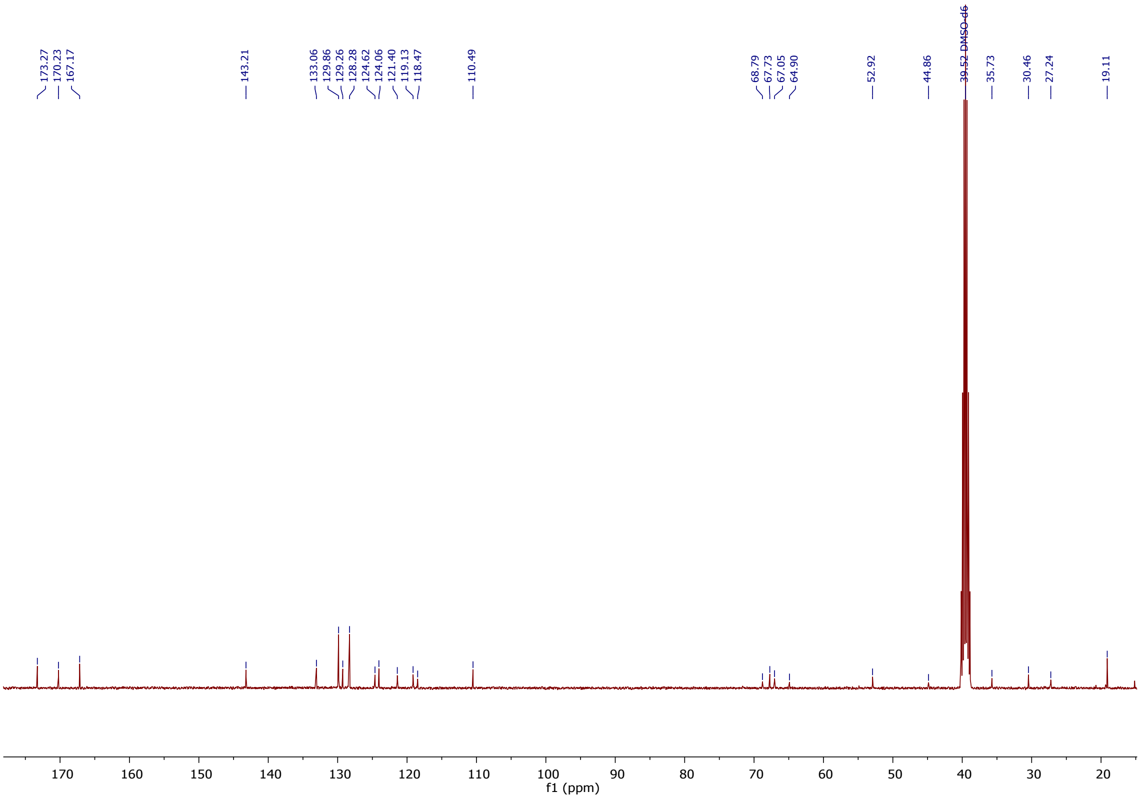

MS spectrum

Compound Table

Compound Label	RT	Mass	Abund	Formula	Tgt Mass	Diff (ppm)
Cpd 1: C89 H84 N8 O24	0.261	1648.56095	1864	C89 H84 N8 O24	1648.55985	0.67

Compound Label	RT	Algorithm	Mass
Cpd 1: C89 H84 N8 O24	0.261	Find By Formula	1648.56095

MS Zoomed Spectrum

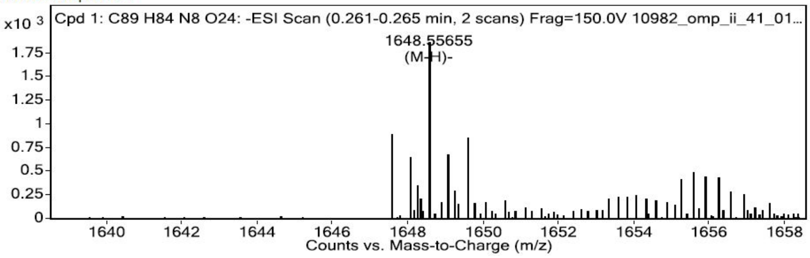

Tetramer 23

^{1}H NMR

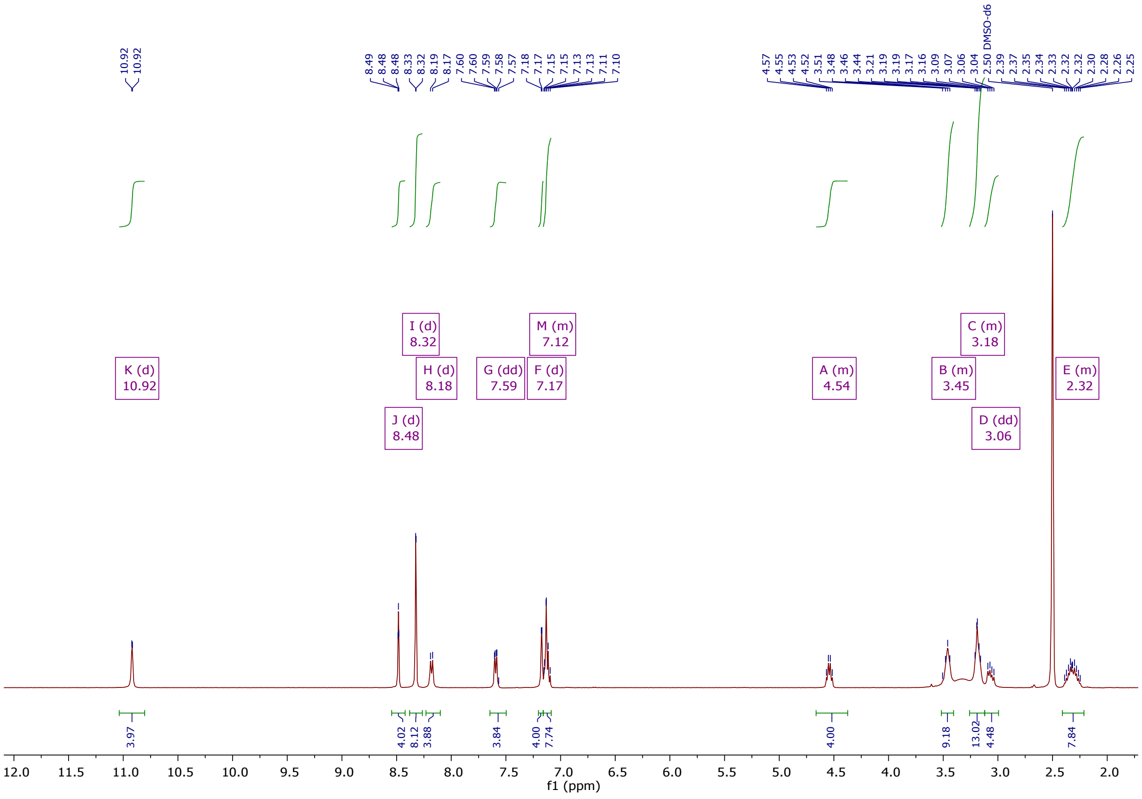

^{13}C NMR

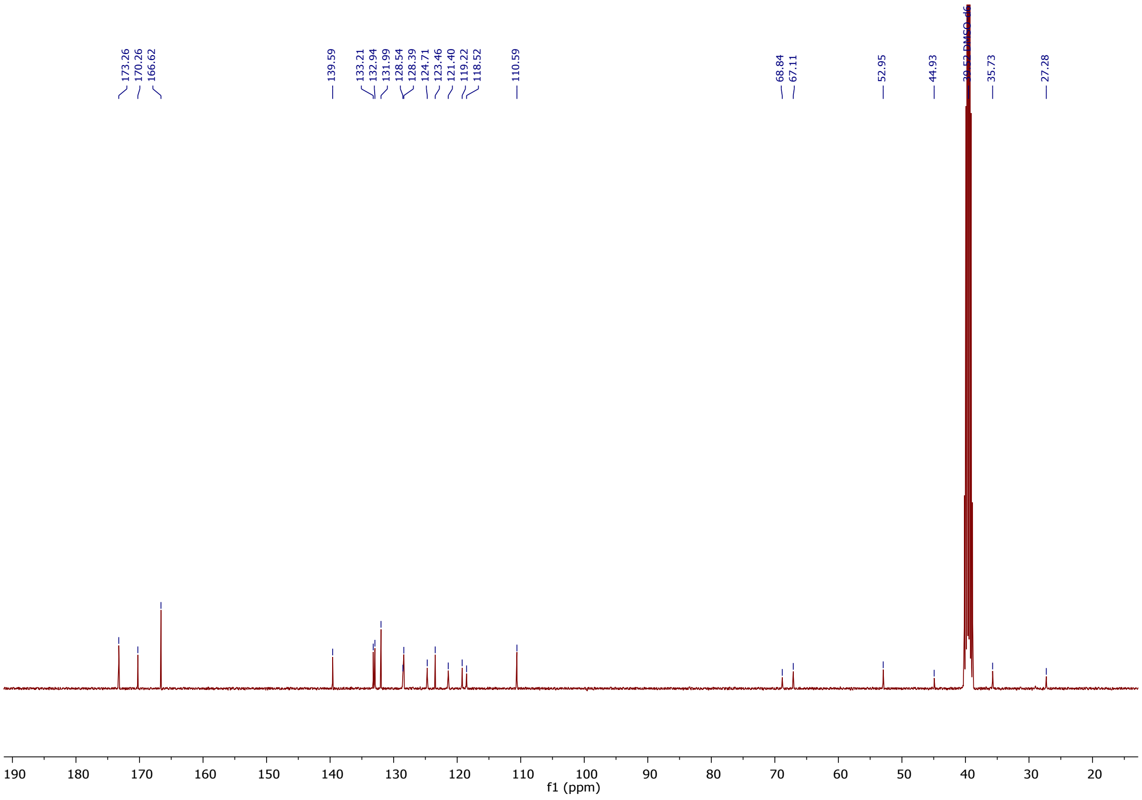

MS spectrum

Compound Table

Compound Label	RT	Mass	Abund	Formula	Tgt Mass	Diff (ppm)
Cpd 1: C93 H84 N8 O32	0.376	1824.52142	11377	C93 H84 N8 O32	1824.51916	1.24

Compound Label	RT	Algorithm	Mass
Cpd 1: C93 H84 N8 O32	0.376	Find By Formula	1824.52142

MS Zoomed Spectrum

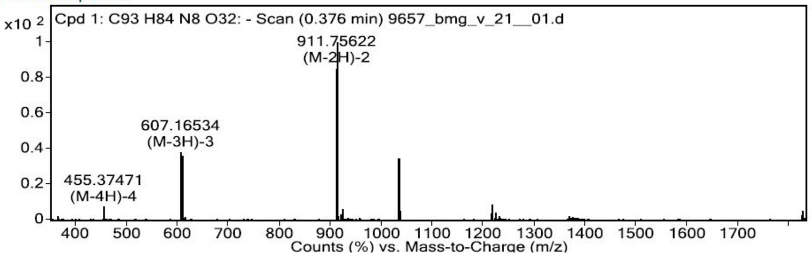

Compound **24**

^{1}H NMR

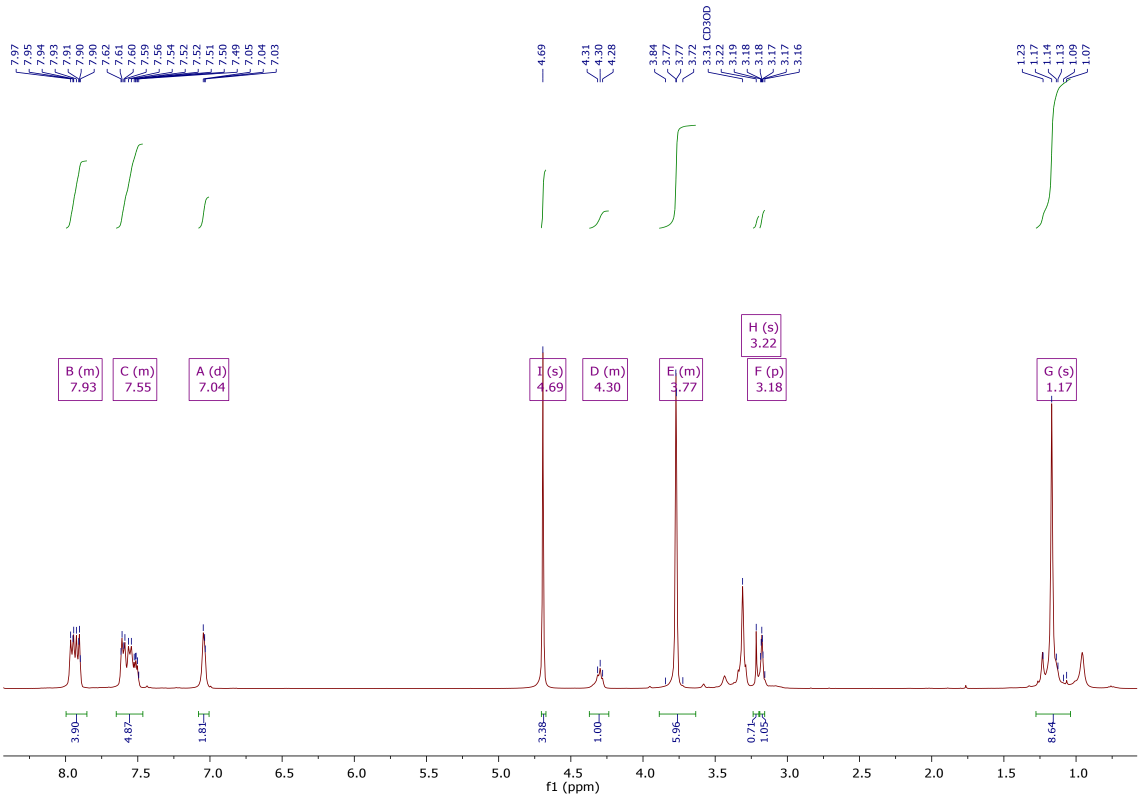

Compound **25**

^{1}H NMR

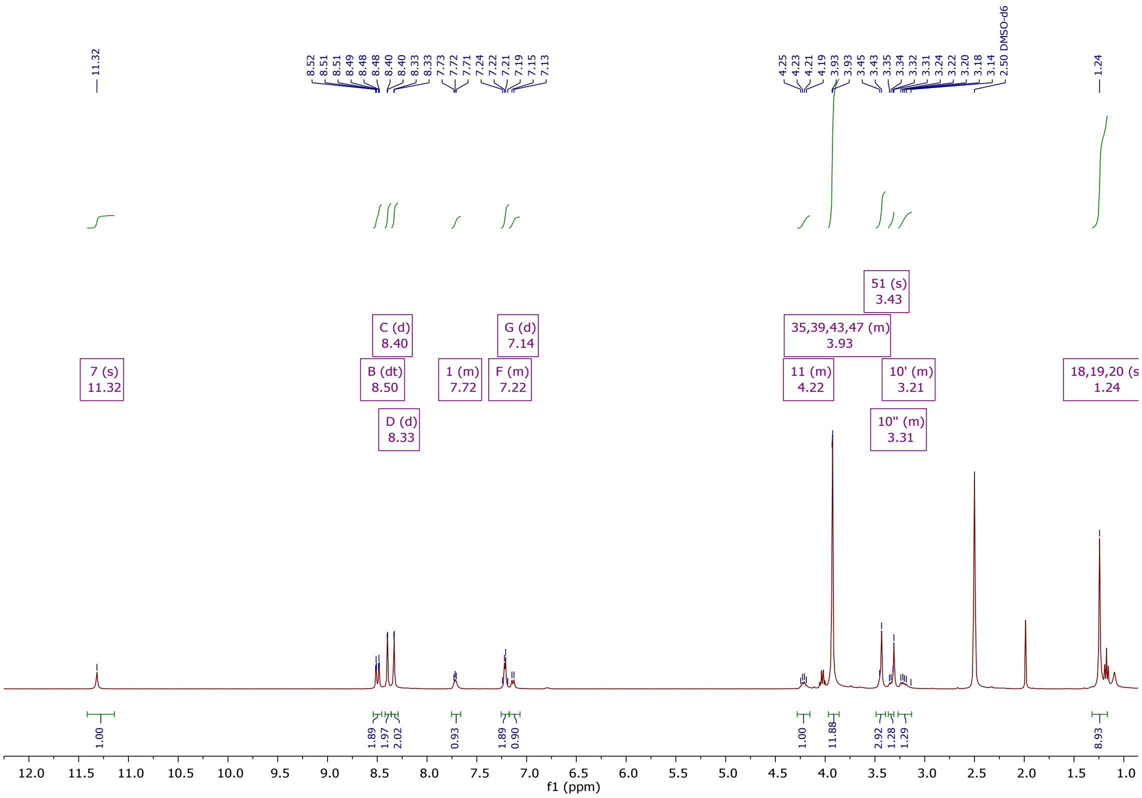

Compound **26**

^{1}H NMR

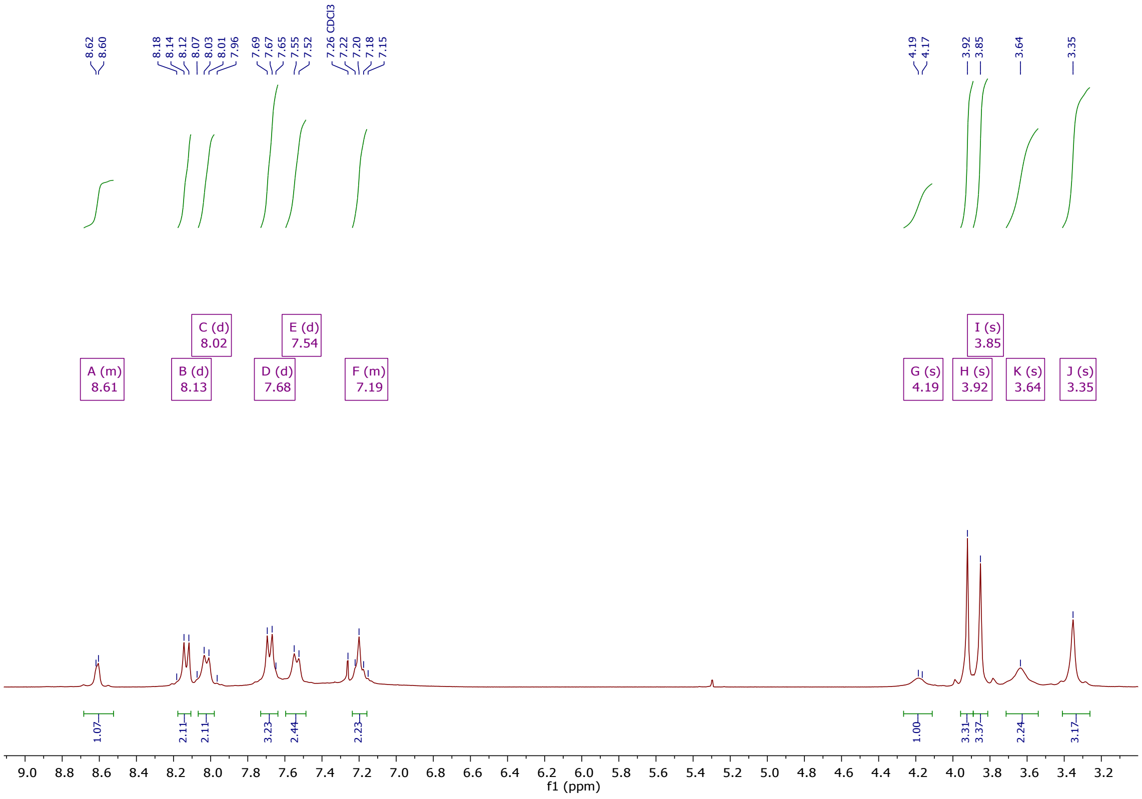

Compound **27**

^{1}H NMR

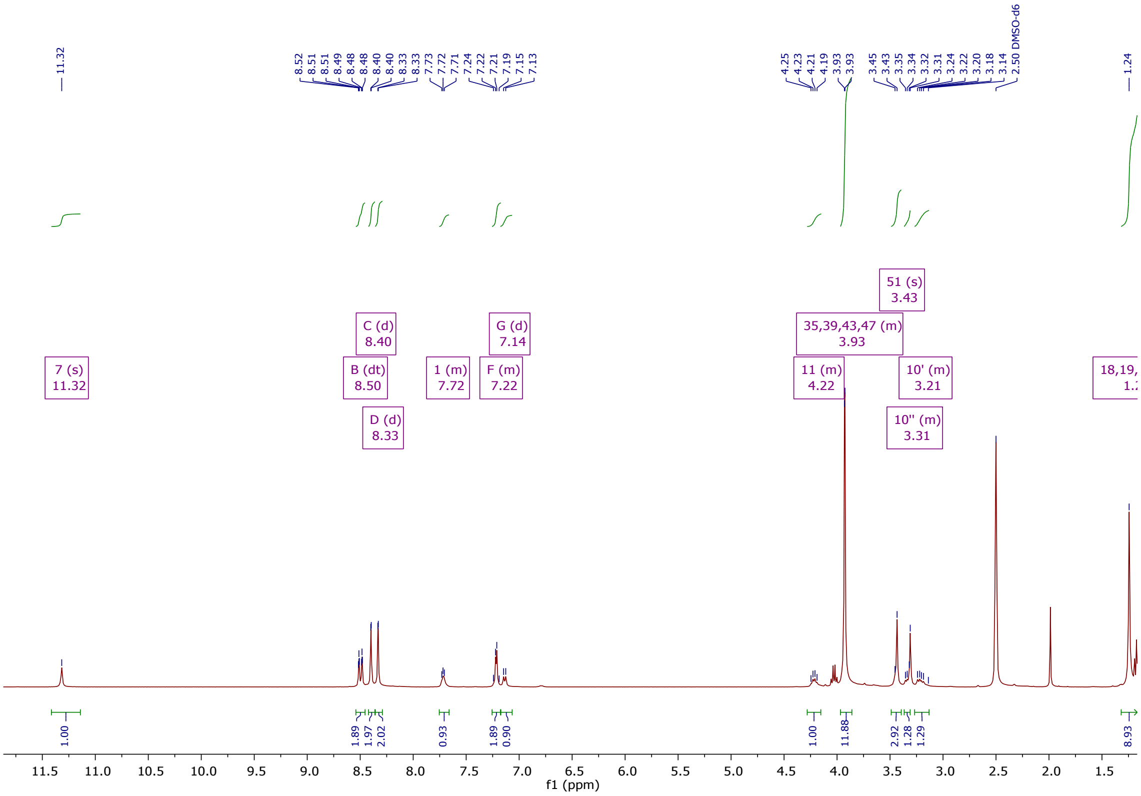

Trimer **28**

^{1}H NMR

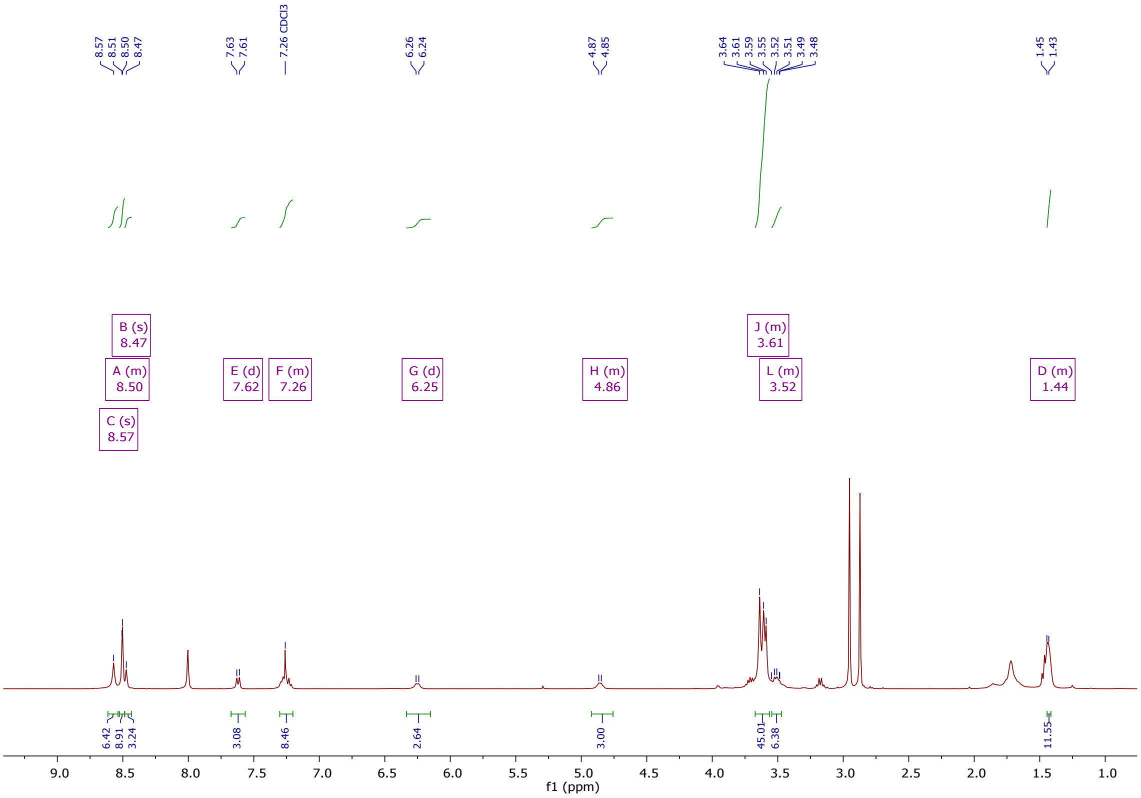

Tetramer **29**

^{1}H NMR

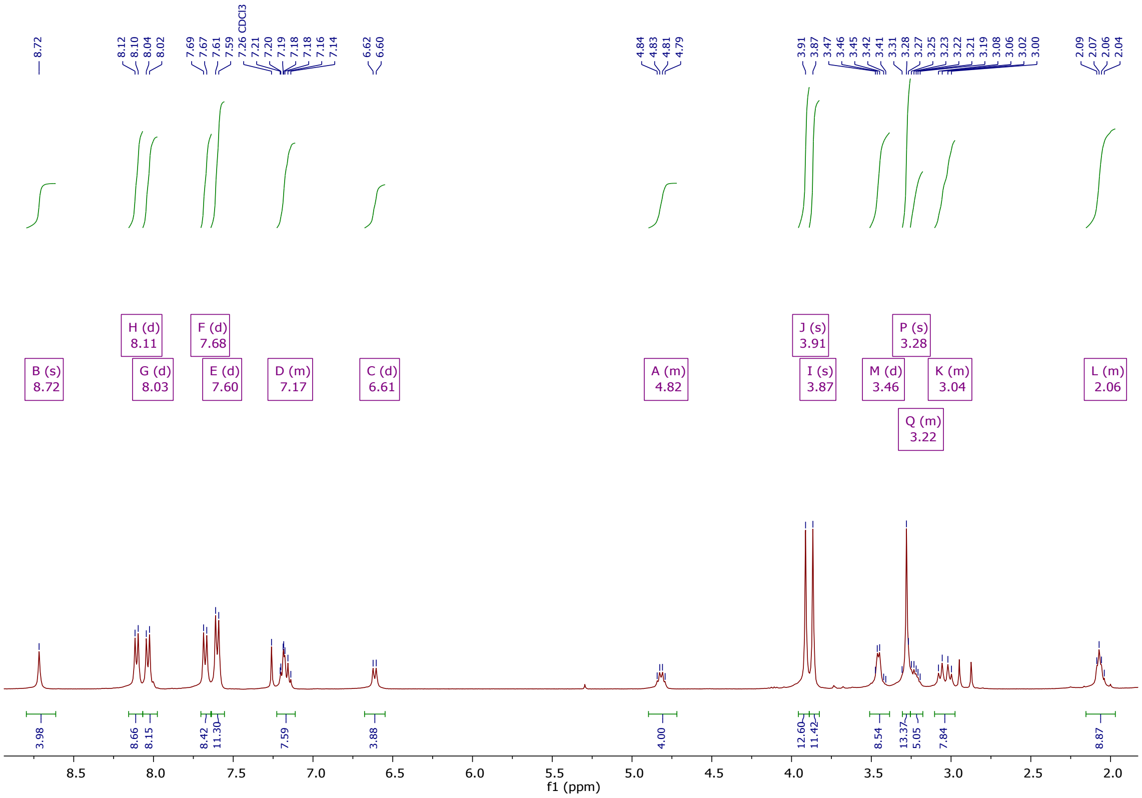

Tetramer **30**

^{1}H NMR

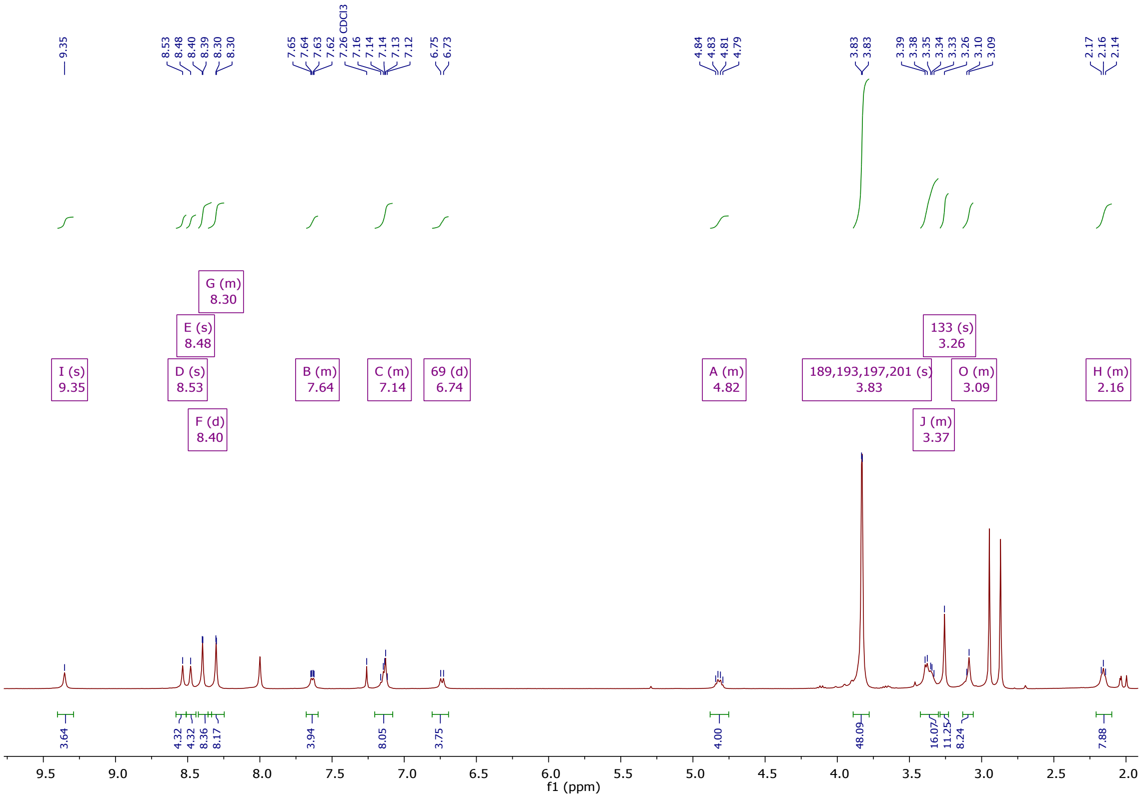

Trimer **31**

^{1}H NMR

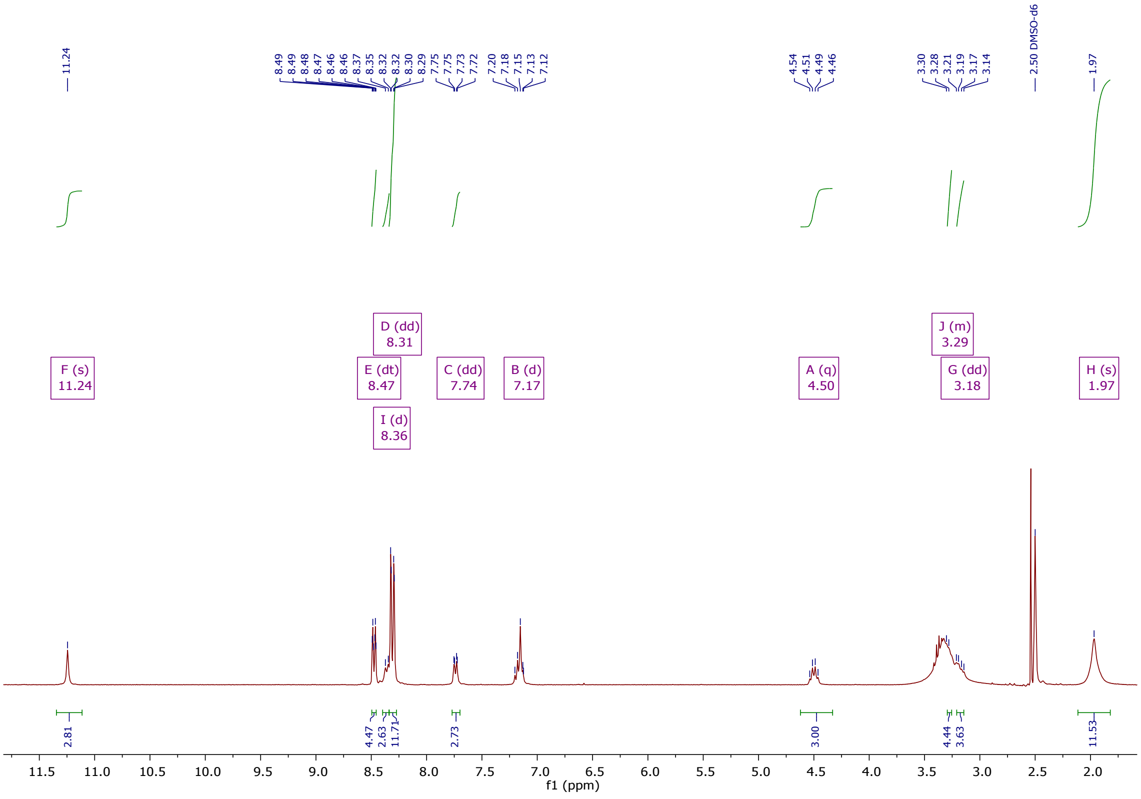

^{13}C NMR

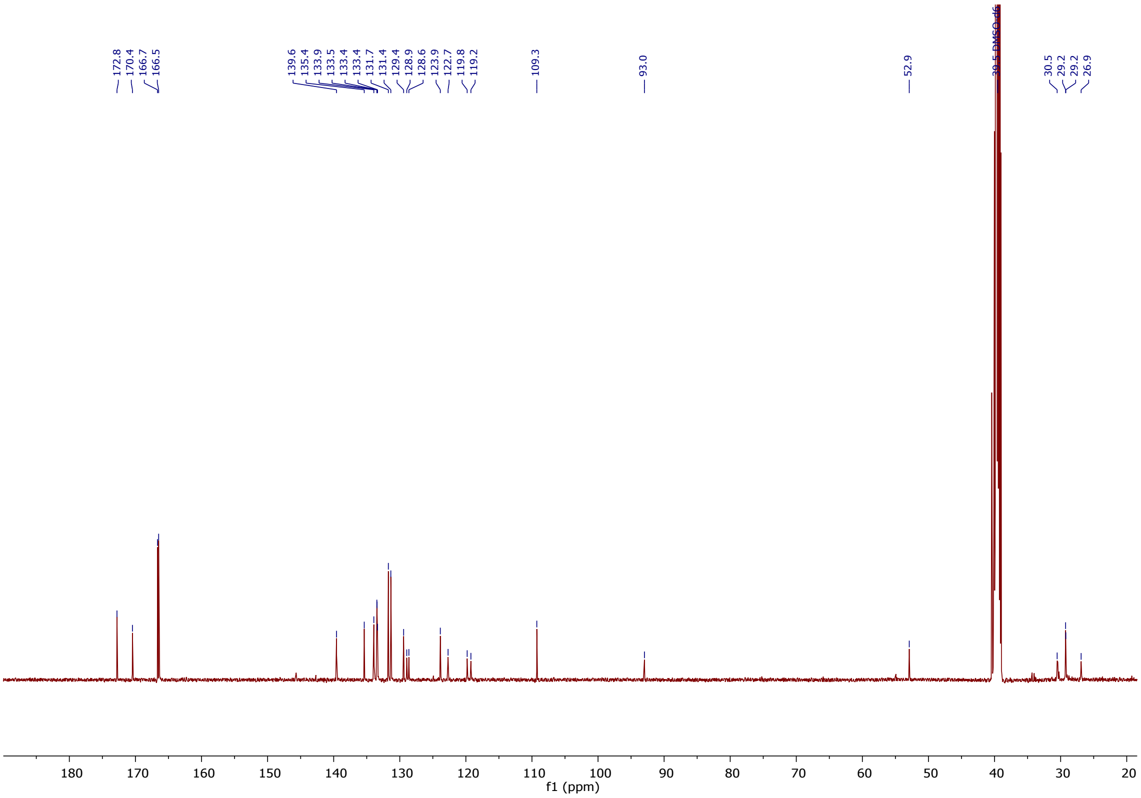

MS Spectrum

Compound Table

Compound Label	RT	Mass	Abund	Formula	Tgt Mass	Diff (ppm)
Cpd 1: C91 H67 N7 O35	0.2	1817.36886	8425	C91 H67 N7 O35	1817.36781	0.58

Compound Label	RT	Algorithm	Mass
Cpd 1: C91 H67 N7 O35	0.2	Find By Formula	1817.36886

MS Zoomed Spectrum

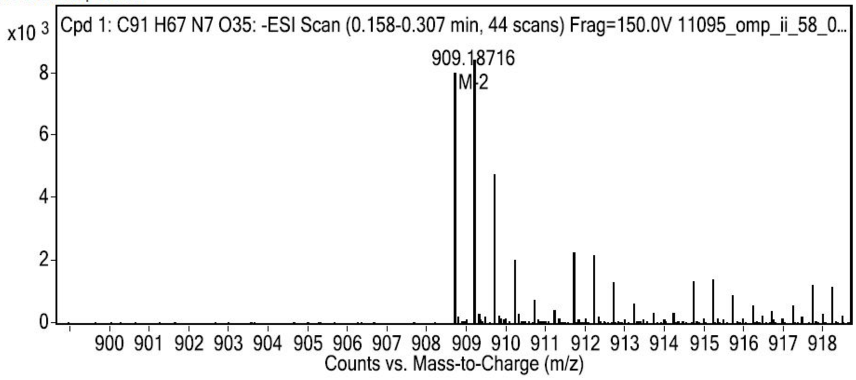

Tetramer **32**

^{1}H NMR

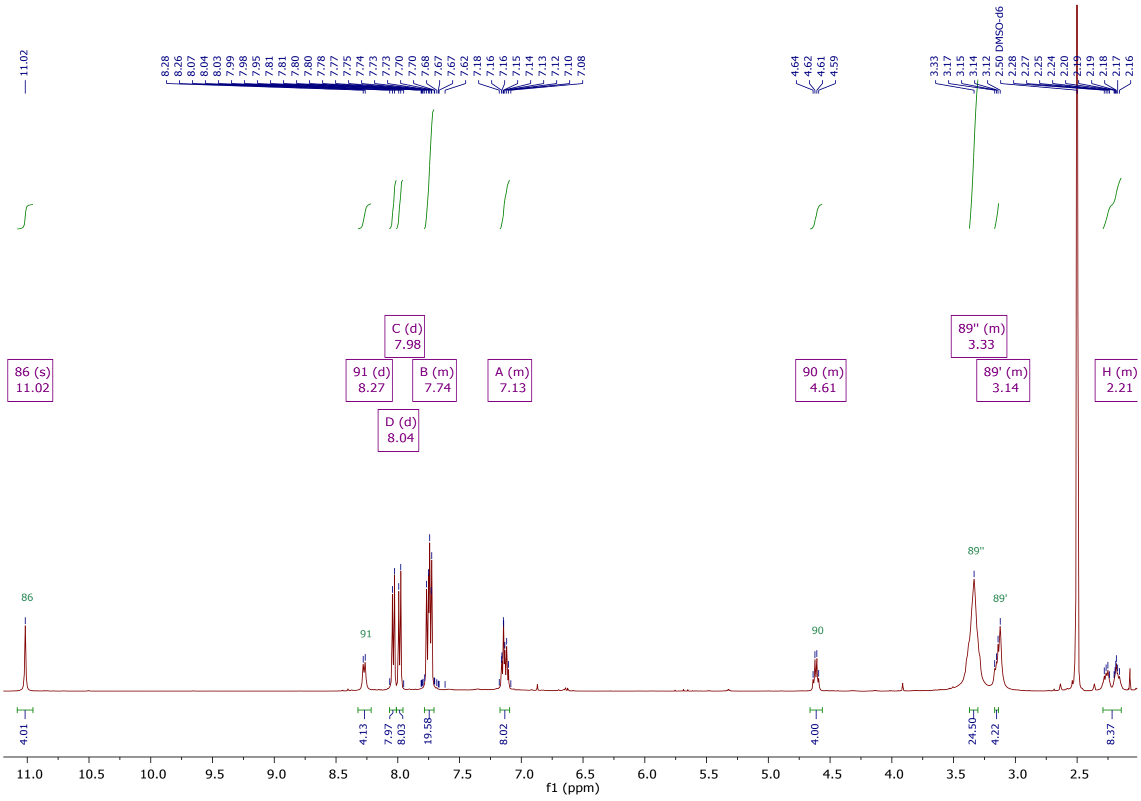

^{13}C NMR

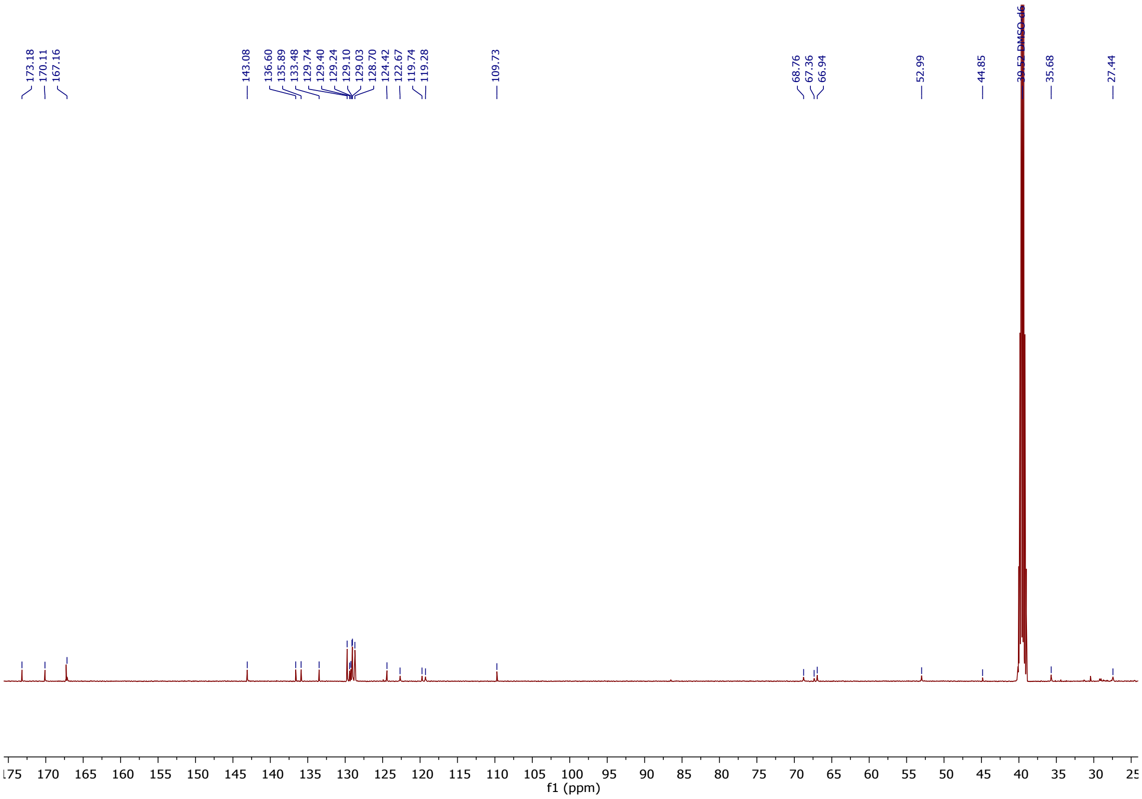

MS Spectrum

Compound Table

Compound Label	RT	Mass	Abund	Formula	Tgt Mass	Diff (ppm)
Cpd 1: C117 H98 N8 O32	0.263	2126.62712	30515	C117 H98 N8 O32	2126.62871	-0.75

Compound Label	RT	Algorithm	Mass
Cpd 1: C117 H98 N8 O32	0.263	Find By Formula	2126.62712

MS Zoomed Spectrum

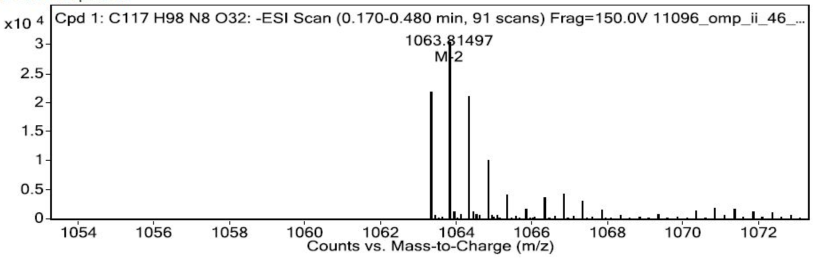

Tetramer **33**

^{1}H NMR

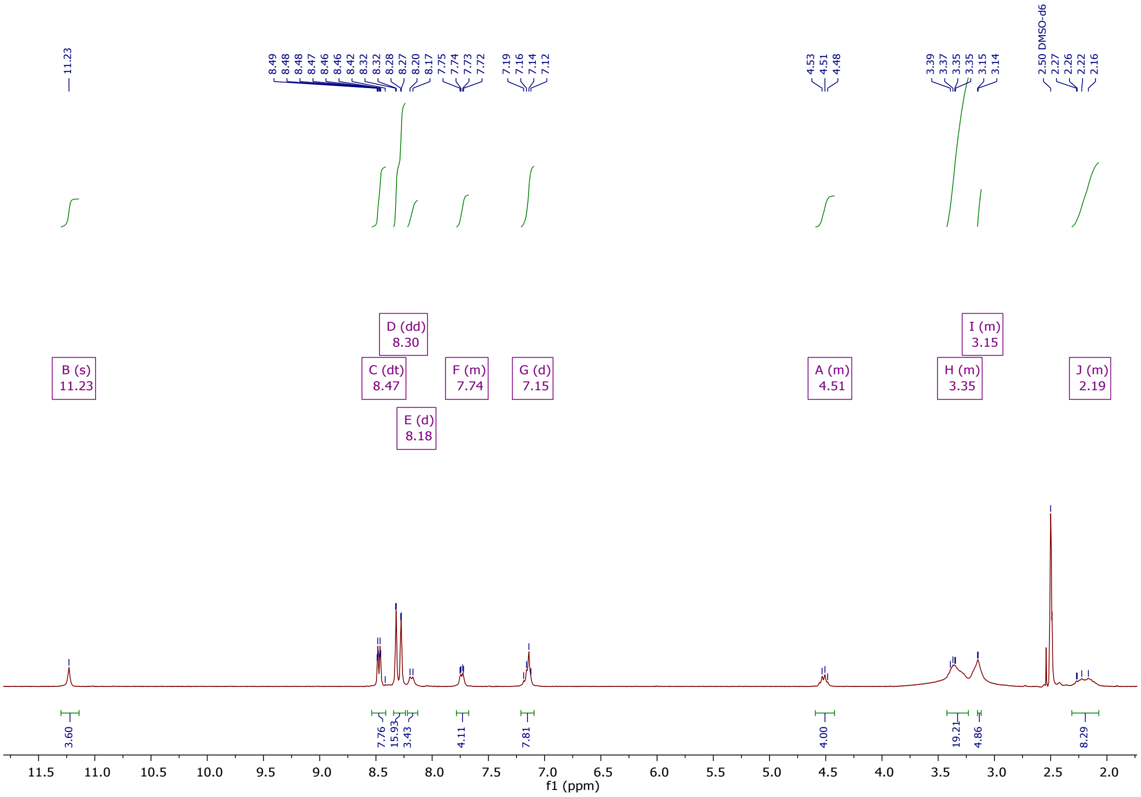

^{13}C NMR

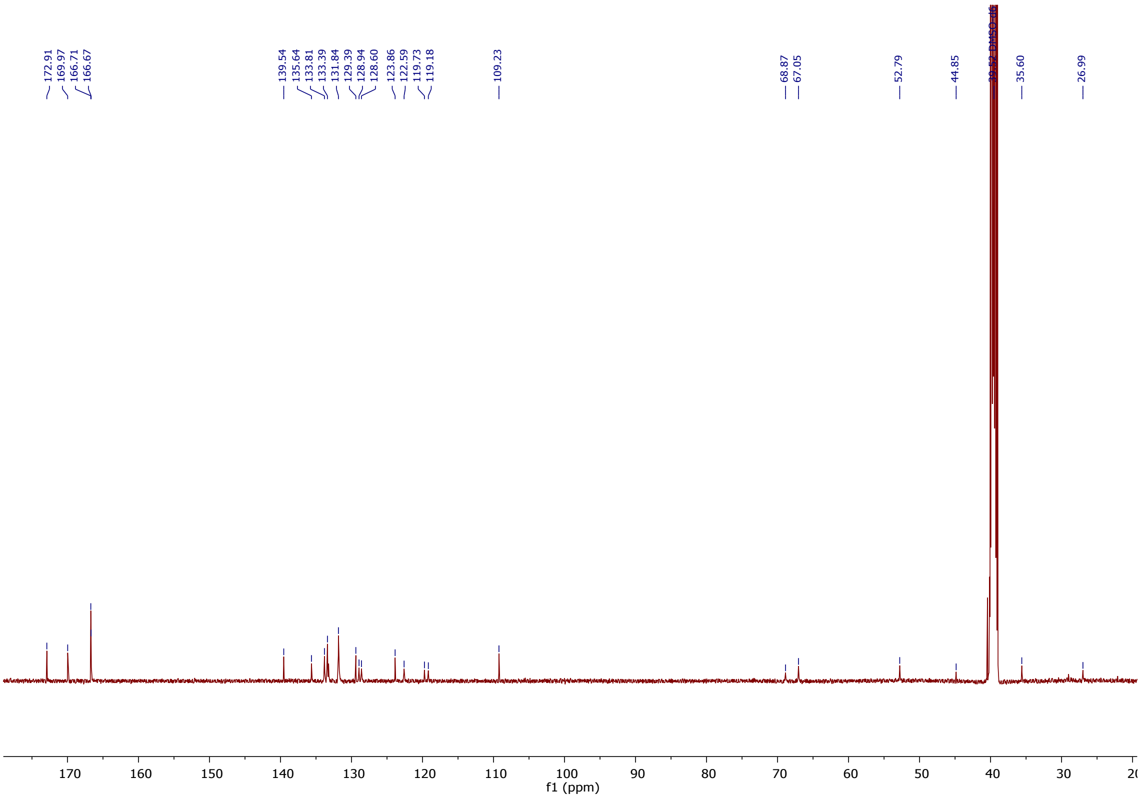

MS Spectrum

Compound Table

Compound Label	RT	Mass	Abund	Formula	Tgt Mass	Diff (ppm)
Cpd 1: C125 H96 N8 O48	0.404	2476.5306	4364	C125 H96 N8 O48	2476.5317	-0.44

Compound Label	RT	Algorithm	Mass
Cpd 1: C125 H96 N8 O48	0.404	Find By Formula	2476.5306

MS Zoomed Spectrum

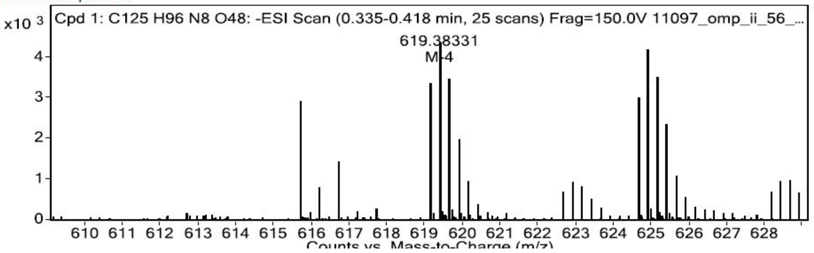

Compound **35**

^{1}H NMR

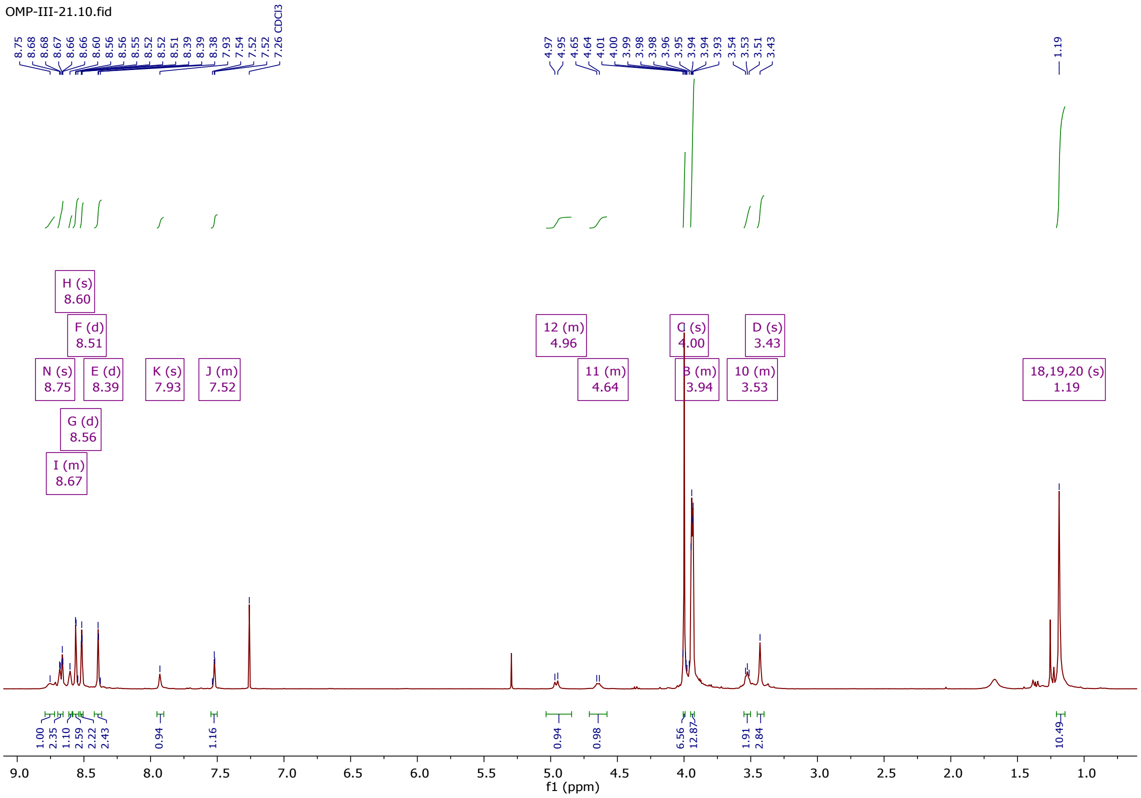

^{13}C NMR

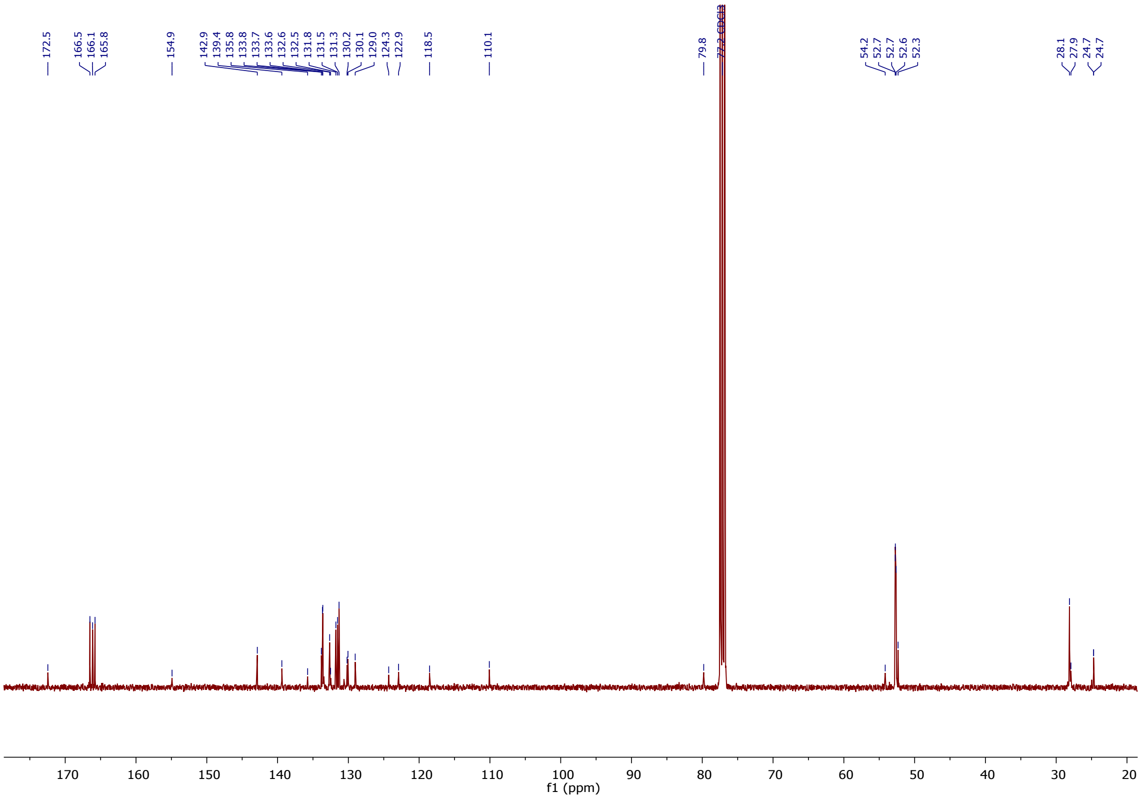

MS Spectrum

Compound Table

Compound Label	RT	Mass	Abund	Formula	Tgt Mass	Diff (ppm)
Cpd 1: C47 H46 N2 O16	0.232	894.2837	5184	C47 H46 N2 O16	894.28473	-1.15

Compound Label	RT	Algorithm	Mass
Cpd 1: C47 H46 N2 O16	0.232	Find By Formula	894.2837

MS Zoomed Spectrum

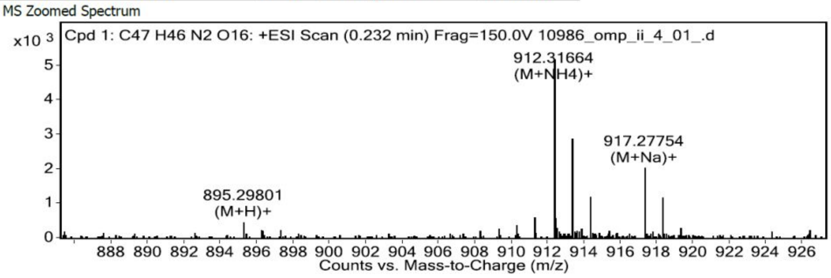

Compound **37**

^{1}H NMR

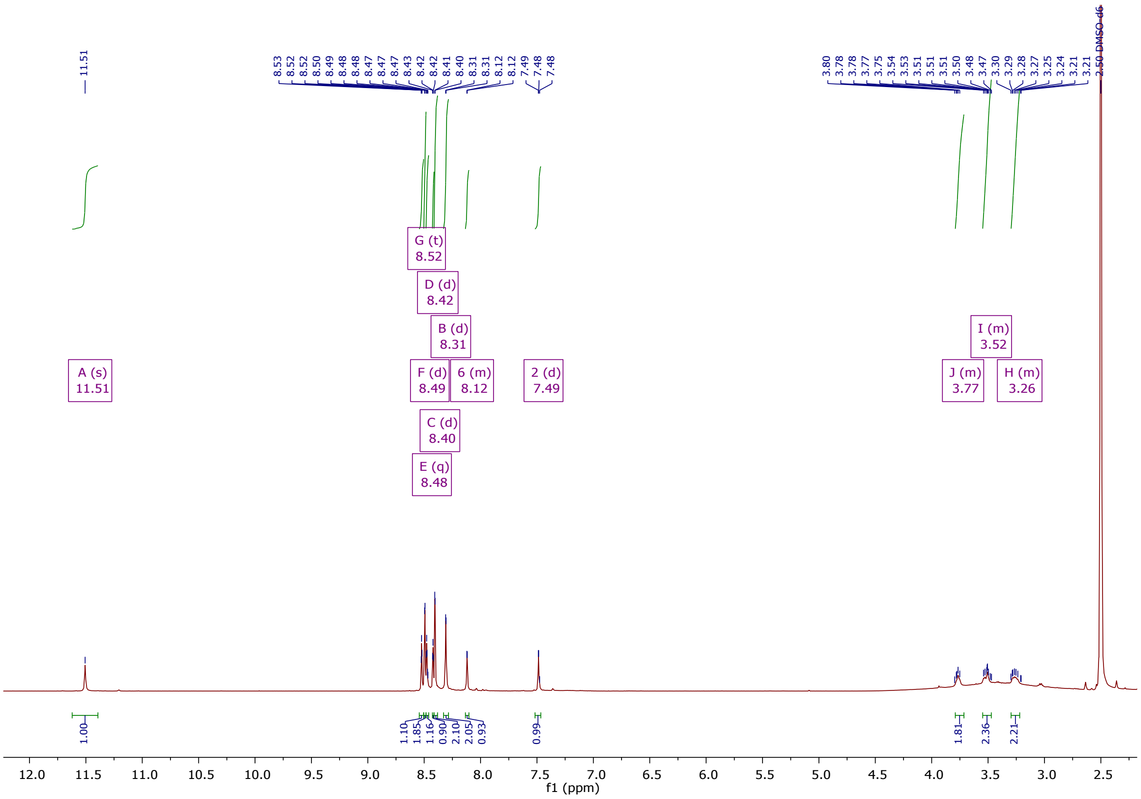

^{13}C NMR

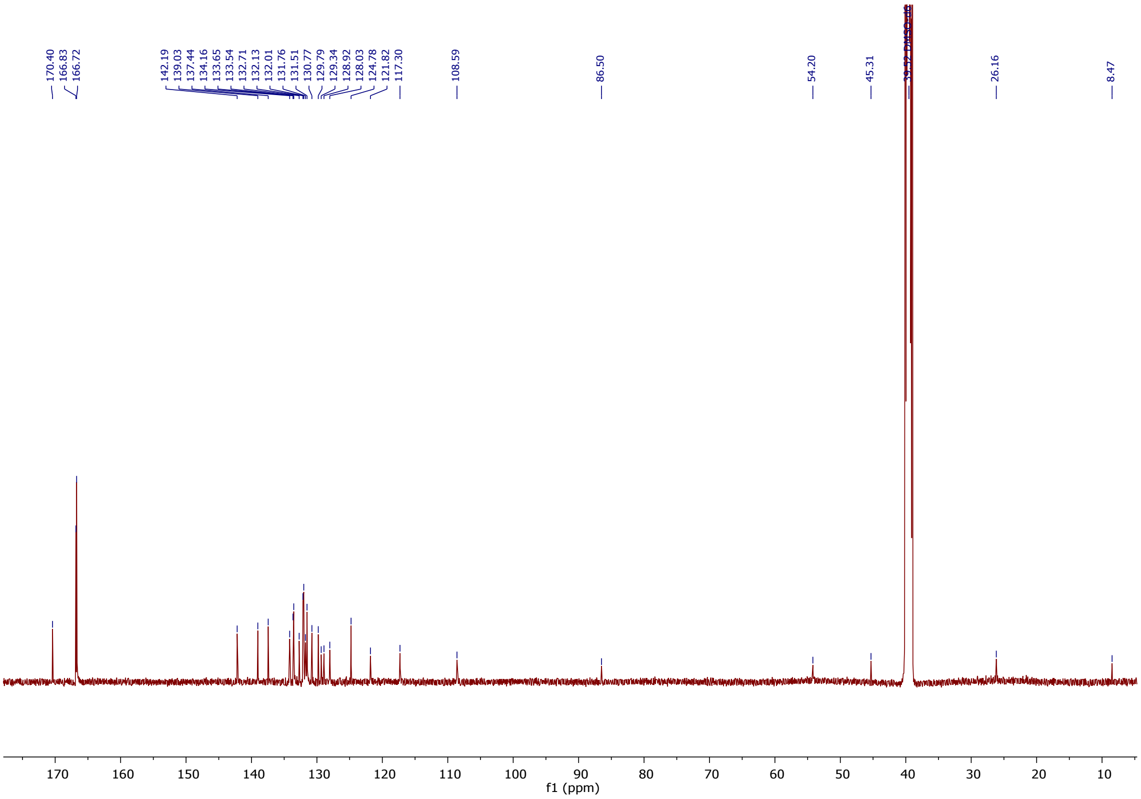

MS Spectrum

Compound Table

Compound Label	RT	Mass	Abund	Formula	Tgt Mass	Diff (ppm)
Cpd 1: C35 H25 N2 O14 Cl	0.281	732.09918	1805	C35 H25 N2 O14 Cl	732.09943	-0.34

Compound Label	RT	Algorithm	Mass
Cpd 1: C35 H25 N2 O14 Cl	0.281	Find By Formula	732.09918

MS Zoomed Spectrum

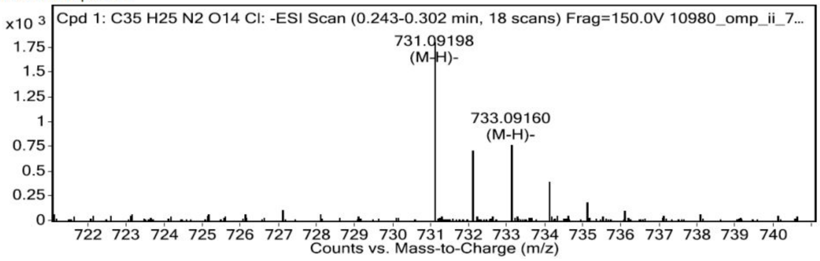

1.2. *HPLC chromatograms of the final compounds*

Compound **14**

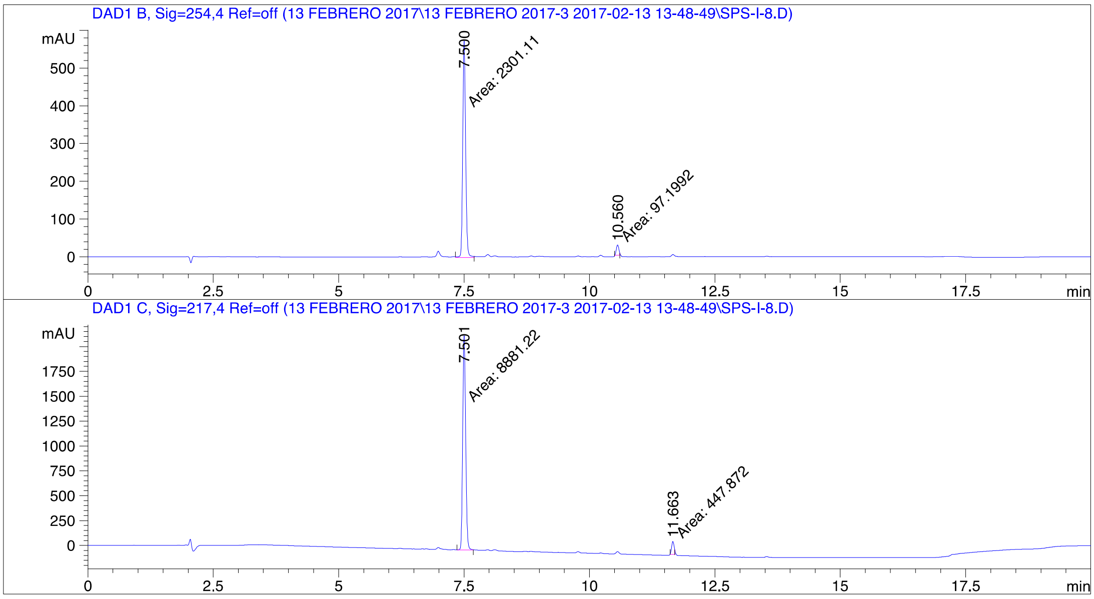

===

Area Percent Report

===

Sorted By : Signal
Multiplier: : 1.0000
Dilution: : 1.0000
Use Multiplier & Dilution Factor with ISTDs

Signal 1: DAD1 B, Sig=254,4 Ref=off

Peak #	RetTime [min]	Type	Width [min]	Area [mAU*s]	Height [mAU]	Area %
1	7.500	MM T	0.1049	2301.10669	573.39423	95.9472
2	10.560	MM T	0.0589	97.19923	27.52111	4.0528

Totals : 2398.30592 600.91533

Signal 2: DAD1 C, Sig=217,4 Ref=off

Peak #	RetTime [min]	Type	Width [min]	Area [mAU*s]	Height [mAU]	Area %
1	7.501	MM T	0.0685	8881.22266	2159.72485	95.1992
2	11.663	MM T	0.0568	447.87231	131.50743	4.8008

Totals : 9329.09497 2291.23228

===

*** End of Report ***

Compound **15**

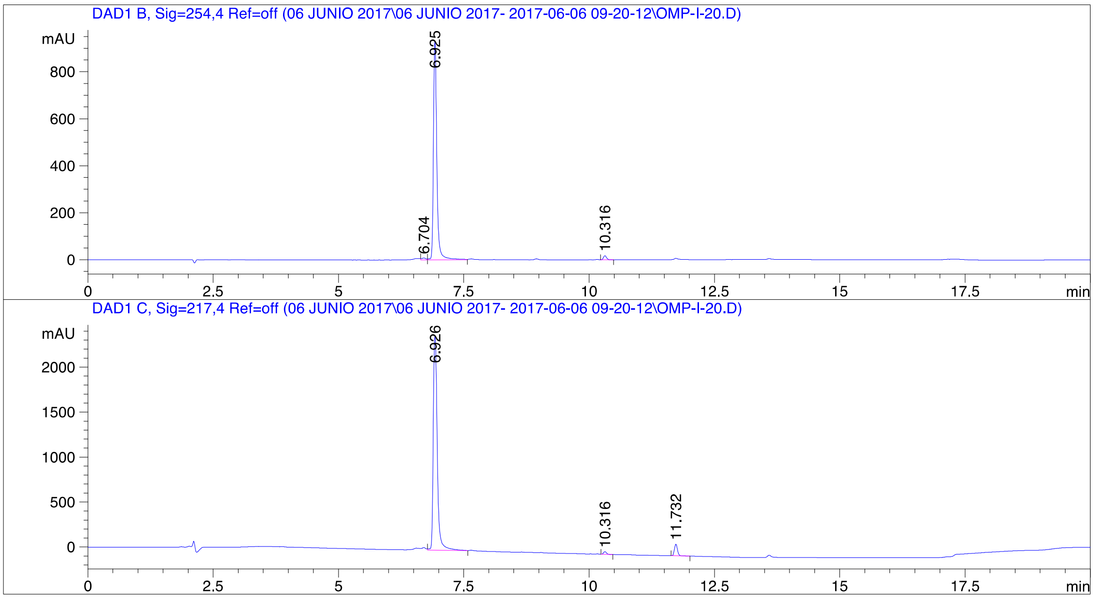

===

Area Percent Report

===

Sorted By : Signal
Multiplier: : 1.0000
Dilution: : 1.0000
Use Multiplier & Dilution Factor with ISTDs

Signal 1: DAD1 B, Sig=254,4 Ref=off

Peak #	RetTime [min]	Type	Width [min]	Area [mAU*s]	Height [mAU]	Area %
1	6.704	VV	0.0858	48.42249	7.86913	1.0800
2	6.925	VV	0.0706	4360.35938	932.47852	97.2477
3	10.316	VB	0.0657	74.98486	17.59751	1.6724

Totals : 4483.76672 957.94516

Signal 2: DAD1 C, Sig=217,4 Ref=off

Peak #	RetTime [min]	Type	Width [min]	Area [mAU*s]	Height [mAU]	Area %
1	6.926	VB	0.0865	1.31697e4	2381.74048	95.1146
2	10.316	VB	0.0663	130.12022	30.17480	0.9398
3	11.732	BV	0.0656	546.31470	128.60342	3.9456

Totals : 1.38462e4 2540.51870

Compound **16**

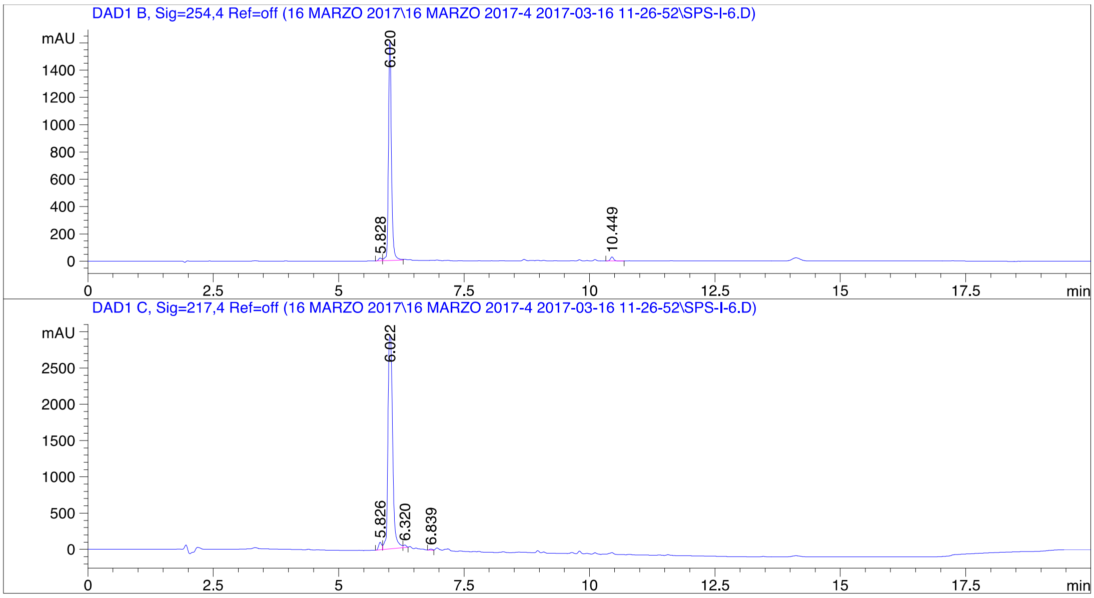

===
Area Percent Report
===

Sorted By : Signal
Multiplier: : 1.0000
Dilution: : 1.0000
Use Multiplier & Dilution Factor with ISTDs

Signal 1: DAD1 B, Sig=254,4 Ref=off

Peak #	RetTime [min]	Type	Width [min]	Area [mAU*s]	Height [mAU]	Area %
1	5.828	BV	0.0653	89.56109	21.20127	1.3036
2	6.020	VV	0.0621	6638.66650	1609.85986	96.6273
3	10.449	VB	0.0713	142.15880	29.99994	2.0692

Totals : 6870.38639 1661.06107

Signal 2: DAD1 C, Sig=217,4 Ref=off

Peak #	RetTime [min]	Type	Width [min]	Area [mAU*s]	Height [mAU]	Area %
1	5.826	BV	0.0624	432.45148	104.15251	2.1997
2	6.022	VV	0.1014	1.90298e4	2945.89697	96.7969
3	6.320	VB	0.0598	133.79588	34.06579	0.6806
4	6.839	BV	0.0671	63.46068	15.08763	0.3228

Totals : 1.96595e4 3099.20291

Compound **21**

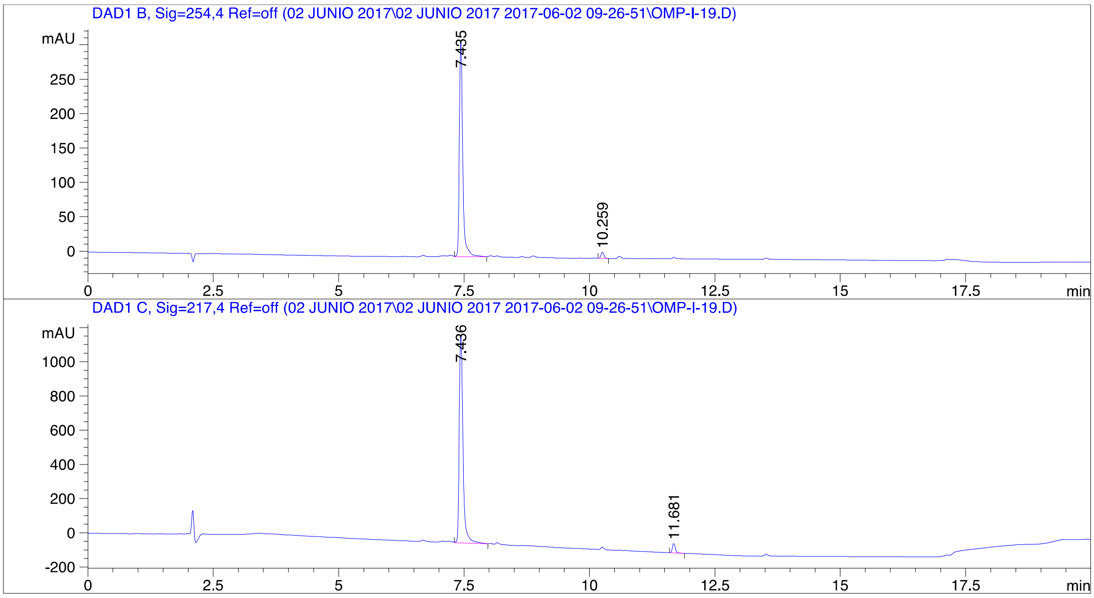

===

Area Percent Report

===

Sorted By : Signal
Multiplier: : 1.0000
Dilution: : 1.0000
Use Multiplier & Dilution Factor with ISTDs

Signal 1: DAD1 B, Sig=254,4 Ref=off

Peak #	RetTime [min]	Type	Width [min]	Area [mAU*s]	Height [mAU]	Area %
1	7.435	VB	0.0706	1473.40234	314.91418	97.5358
2	10.259	VB	0.0644	37.22524	8.97926	2.4642

Totals : 1510.62758 323.89345

Signal 2: DAD1 C, Sig=217,4 Ref=off

Peak #	RetTime [min]	Type	Width [min]	Area [mAU*s]	Height [mAU]	Area %
1	7.436	VV	0.0810	6361.39746	1216.09241	96.5940
2	11.681	BB	0.0639	224.30923	54.62875	3.4060

Totals : 6585.70670 1270.72116

Compound **22**

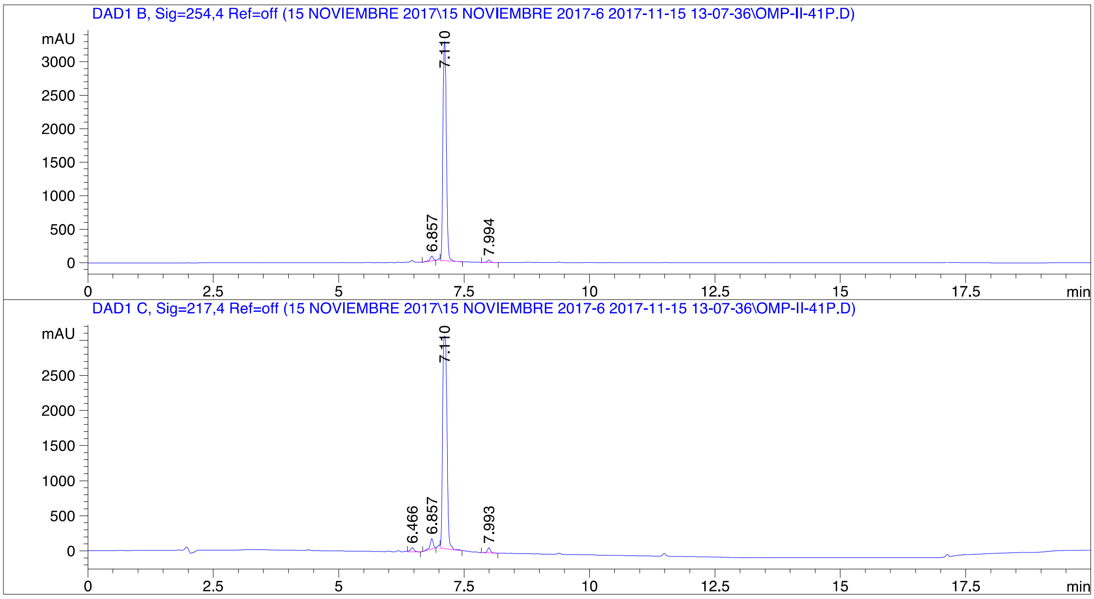

===

Area Percent Report

===

Sorted By : Signal
Multiplier: : 1.0000
Dilution: : 1.0000
Use Multiplier & Dilution Factor with ISTDs

Signal 1: DAD1 B, Sig=254,4 Ref=off

Peak #	RetTime [min]	Type	Width [min]	Area [mAU*s]	Height [mAU]	Area %
1	6.857	BB	0.0671	313.05878	71.41973	1.9485
2	7.110	VB	0.0773	1.55943e4	3288.47388	97.0591
3	7.994	BV	0.0650	159.45134	36.46962	0.9924

Totals : 1.60668e4 3396.36322

Signal 2: DAD1 C, Sig=217,4 Ref=off

Peak #	RetTime [min]	Type	Width [min]	Area [mAU*s]	Height [mAU]	Area %
1	6.466	VB	0.0716	290.91547	58.90950	1.4961
2	6.857	BB	0.0663	627.44574	145.40039	3.2268
3	7.110	VB	0.1001	1.82069e4	3031.51392	93.6331
4	7.993	BB	0.0647	319.67426	73.51591	1.6440

Totals : 1.94449e4 3309.33972

Compound **23**

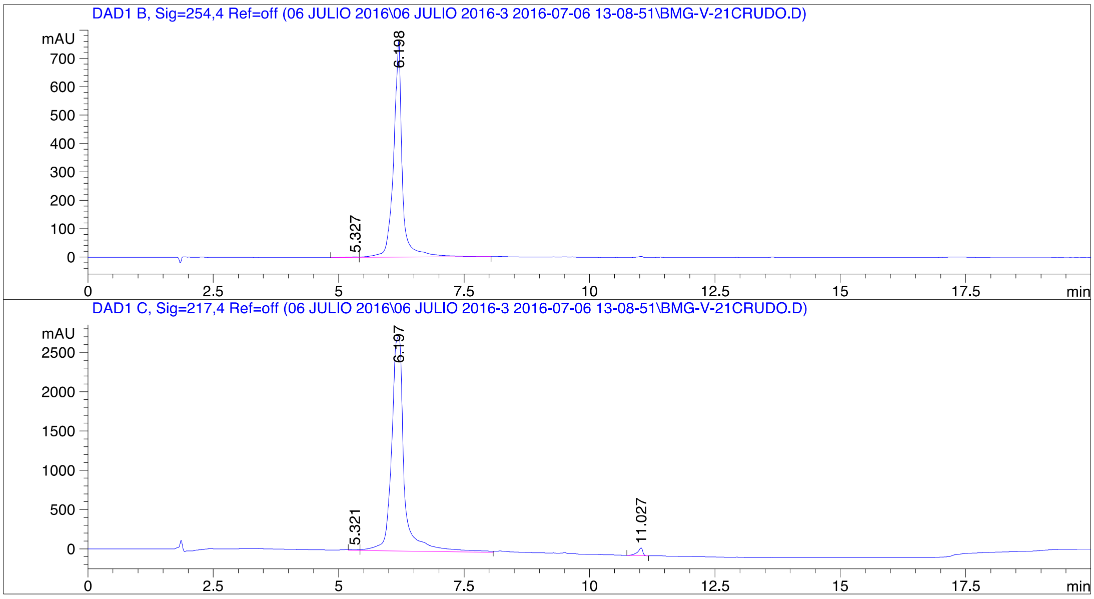


```
=====================================================================
                         Area Percent Report
=====================================================================

Sorted By             :      Signal
Multiplier:                   :      1.0000
Dilution:                     :      1.0000
Use Multiplier & Dilution Factor with ISTDs


Signal 1: DAD1 B, Sig=254,4 Ref=off

Peak RetTime Type  Width     Area      Height     Area
  #   [min]        [min]   [mAU*s]     [mAU]        %
----|-------|----|-------|----------|----------|--------|
   1   5.327 BV    0.2077   29.57592    1.89940   0.3192
   2   6.198 VB    0.1573 9236.43555  762.56097  99.6808

Totals :                  9266.01147  764.46038

Signal 2: DAD1 C, Sig=217,4 Ref=off

Peak RetTime Type  Width     Area      Height     Area
  #   [min]        [min]   [mAU*s]     [mAU]        %
----|-------|----|-------|----------|----------|--------|
   1   5.321 VV    0.1590  152.69135   13.39254   0.3154
   2   6.197 VV    0.2527 4.75705e4  2738.62036  98.2526
   3  11.027 BB    0.1029  693.33771   97.59547   1.4320

Totals :                  4.84165e4  2849.60838
```

Compound **31**

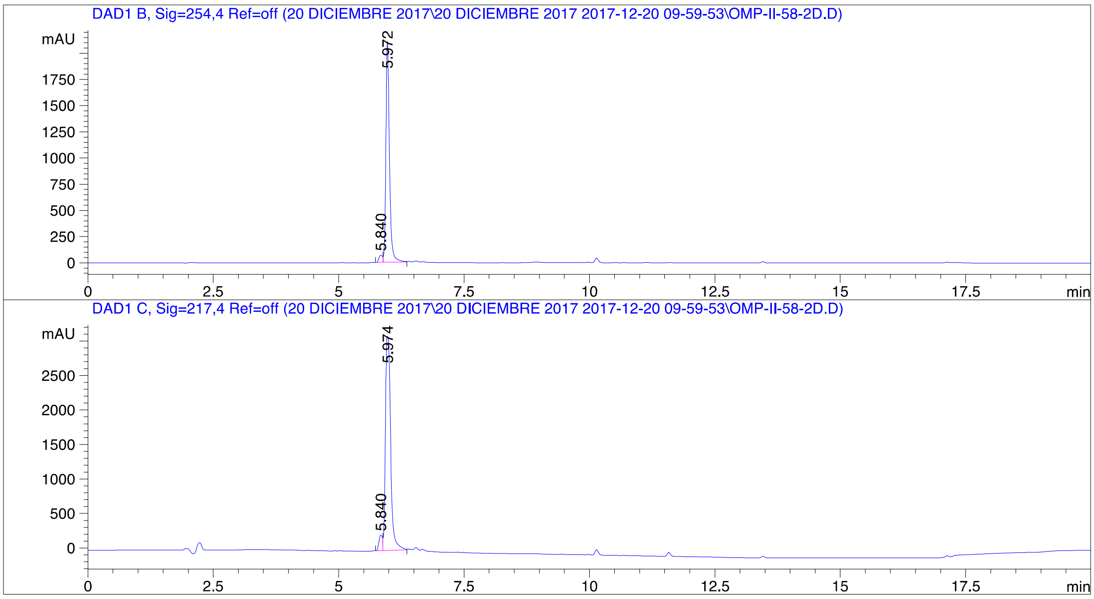

===

Area Percent Report

===

Sorted By : Signal
Multiplier: : 1.0000
Dilution: : 1.0000
Use Multiplier & Dilution Factor with ISTDs

Signal 1: DAD1 B, Sig=254,4 Ref=off

Peak #	RetTime [min]	Type	Width [min]	Area [mAU*s]	Height [mAU]	Area %
1	5.840	BV	0.0769	333.38525	68.39075	3.0787
2	5.972	VV	0.0740	1.04954e4	2109.41113	96.9213

Totals : 1.08288e4 2177.80189

Signal 2: DAD1 C, Sig=217,4 Ref=off

Peak #	RetTime [min]	Type	Width [min]	Area [mAU*s]	Height [mAU]	Area %
1	5.840	BV	0.0746	1086.12329	223.68175	4.5983
2	5.974	VV	0.1147	2.25339e4	3103.50195	95.4017

Totals : 2.36201e4 3327.18370

Compound **32**

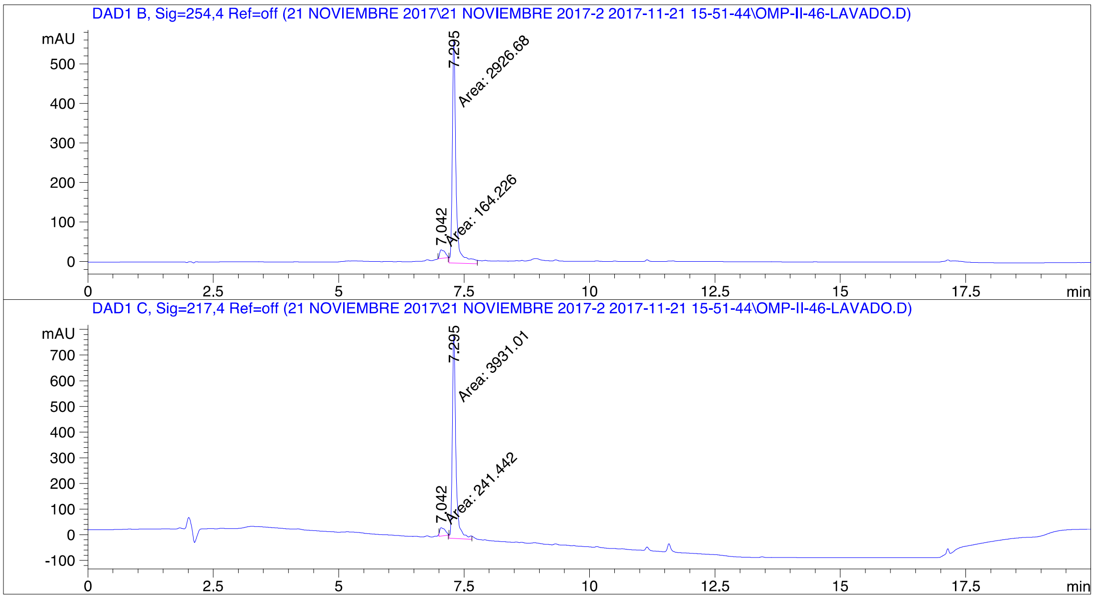

===

Area Percent Report

===

Sorted By : Signal
Multiplier: : 1.0000
Dilution: : 1.0000
Use Multiplier & Dilution Factor with ISTDs

Signal 1: DAD1 B, Sig=254,4 Ref=off

Peak #	RetTime [min]	Type	Width [min]	Area [mAU*s]	Height [mAU]	Area %
1	7.042	MM T	0.1232	164.22618	22.20918	5.3132
2	7.295	MM T	0.0865	2926.67651	563.69714	94.6868

Totals : 3090.90269 585.90633

Signal 2: DAD1 C, Sig=217,4 Ref=off

Peak #	RetTime [min]	Type	Width [min]	Area [mAU*s]	Height [mAU]	Area %
1	7.042	MM T	0.1215	241.44183	33.10678	5.7866
2	7.295	MM T	0.0826	3931.01025	792.73907	94.2134

Totals : 4172.45209 825.84586

Compound **33**

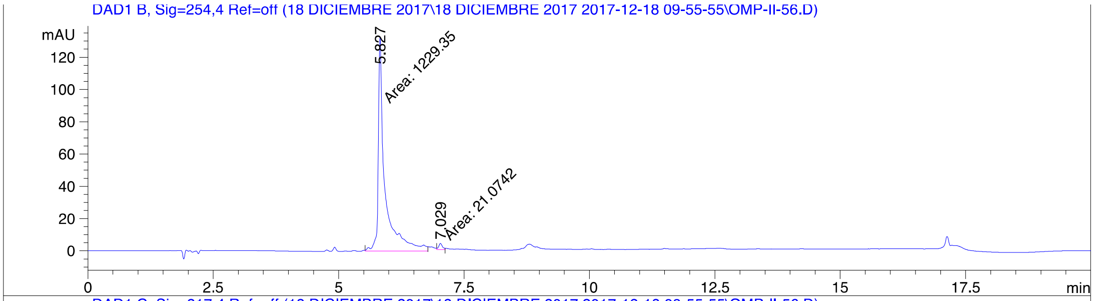

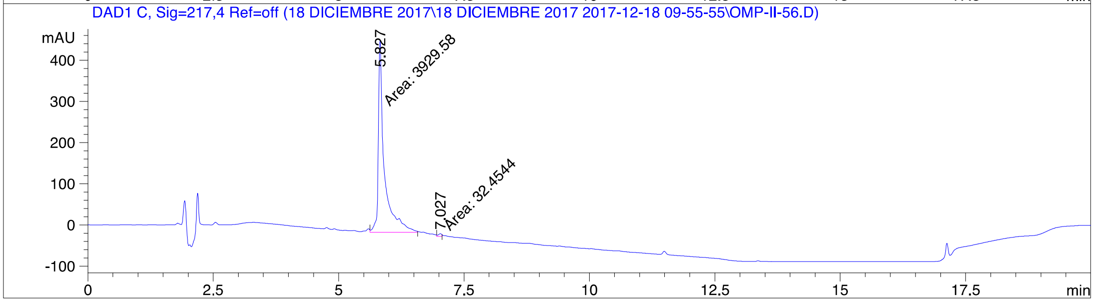

===

Area Percent Report

===

Sorted By : Signal
Multiplier: : 1.0000
Dilution: : 1.0000
Use Multiplier & Dilution Factor with ISTDs

Signal 1: DAD1 B, Sig=254,4 Ref=off

Peak #	RetTime [min]	Type	Width [min]	Area [mAU*s]	Height [mAU]	Area %
1	5.827	MM T	0.1546	1229.35229	132.57002	98.3146
2	7.029	MM T	0.0896	21.07415	3.91994	1.6854

Totals : 1250.42645 136.48996

Signal 2: DAD1 C, Sig=217,4 Ref=off

Peak #	RetTime [min]	Type	Width [min]	Area [mAU*s]	Height [mAU]	Area %
1	5.827	MM T	0.1637	3929.58203	467.08563	99.1809
2	7.027	MM T	0.0854	32.45444	6.71945	0.8191

Totals : 3962.03647 473.80508

1.3. *Two-dimensional spectra of trimer **14** and tetramers **21** and **32***

Compound **14**

COSY

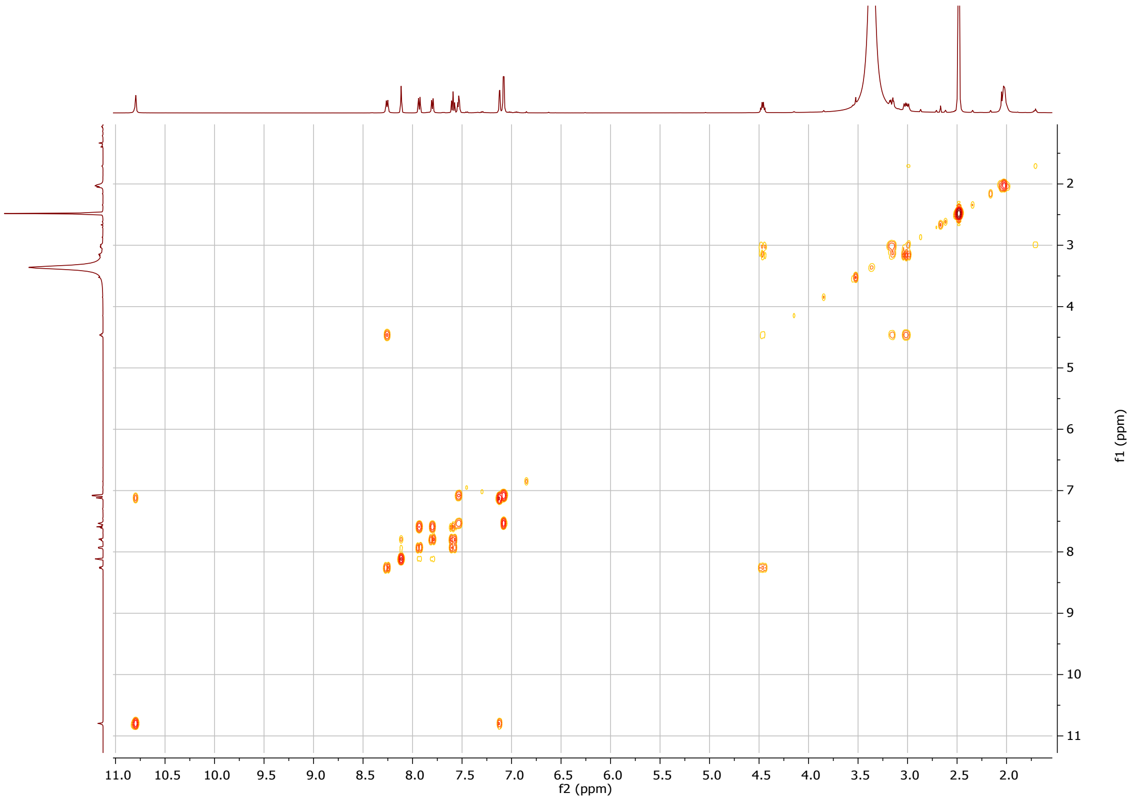

HSQC

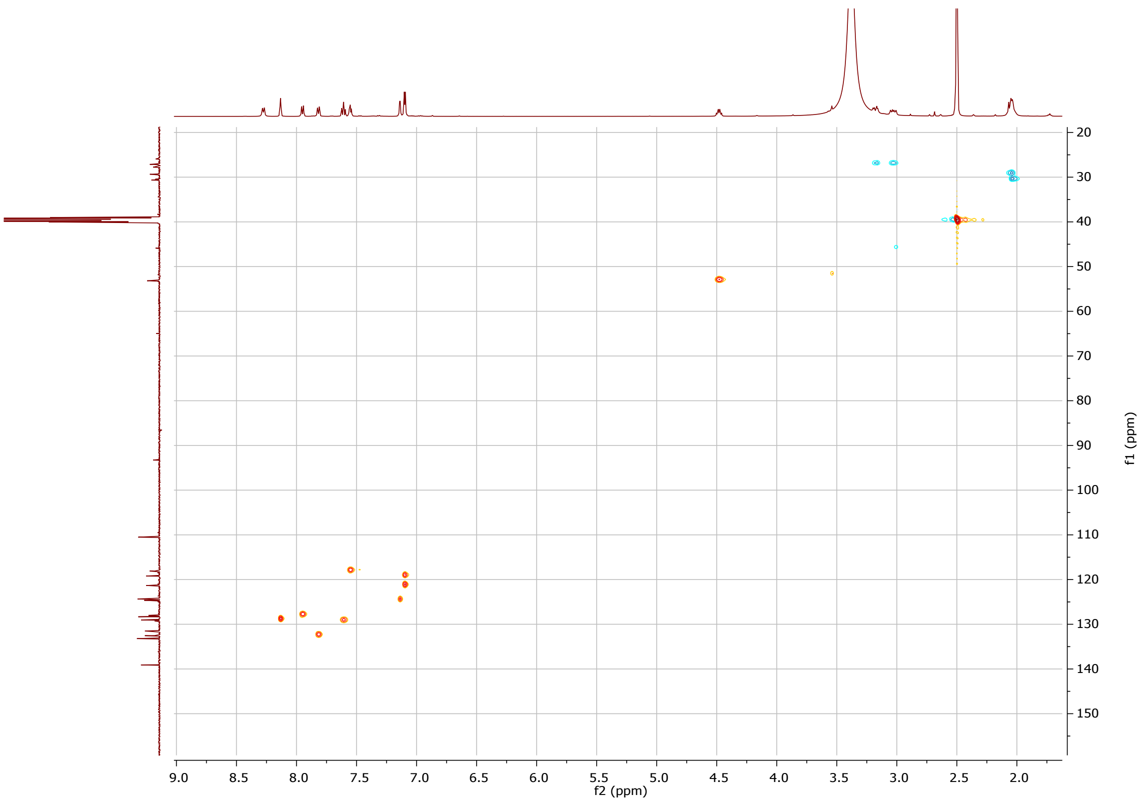

Compound **21**

COSY

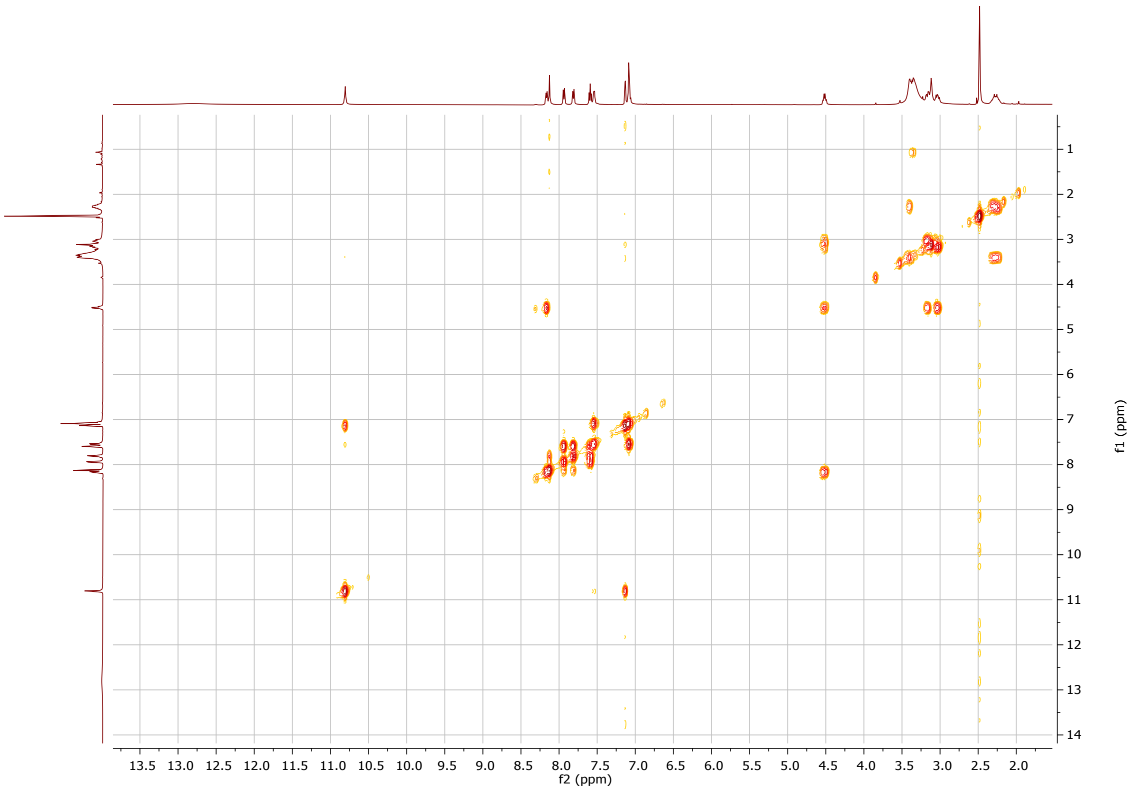

HSQC

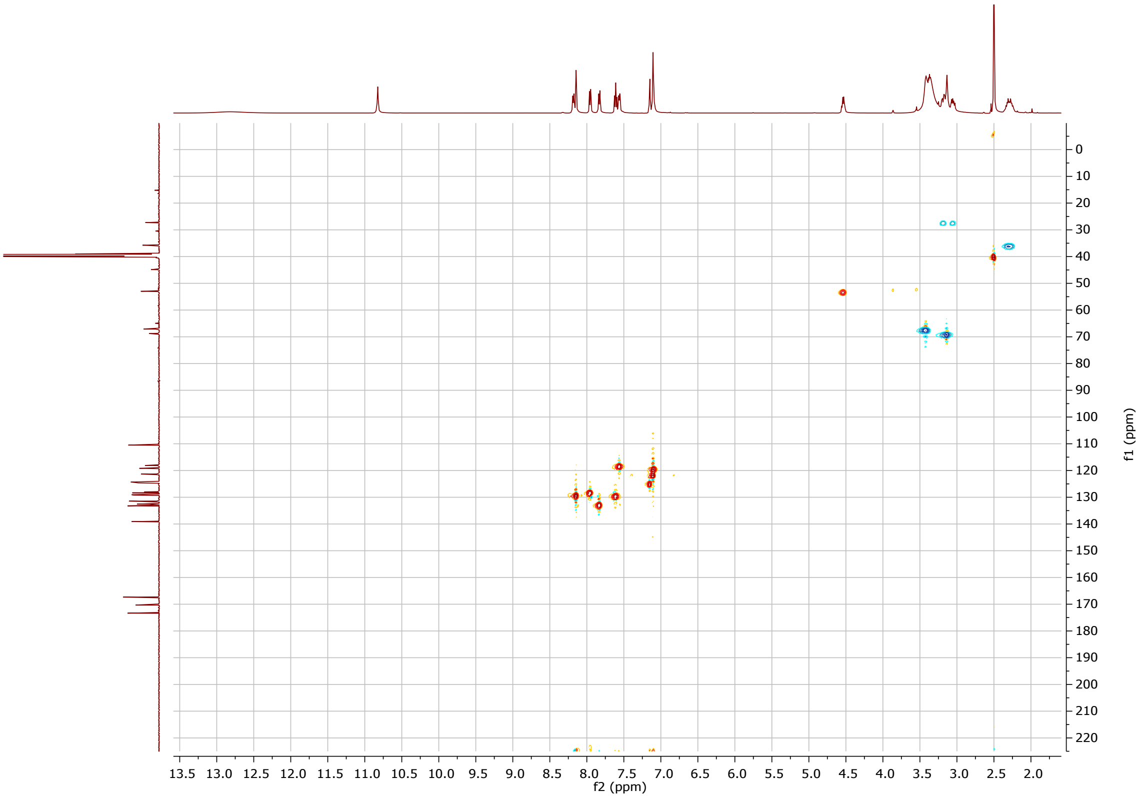

HMBC

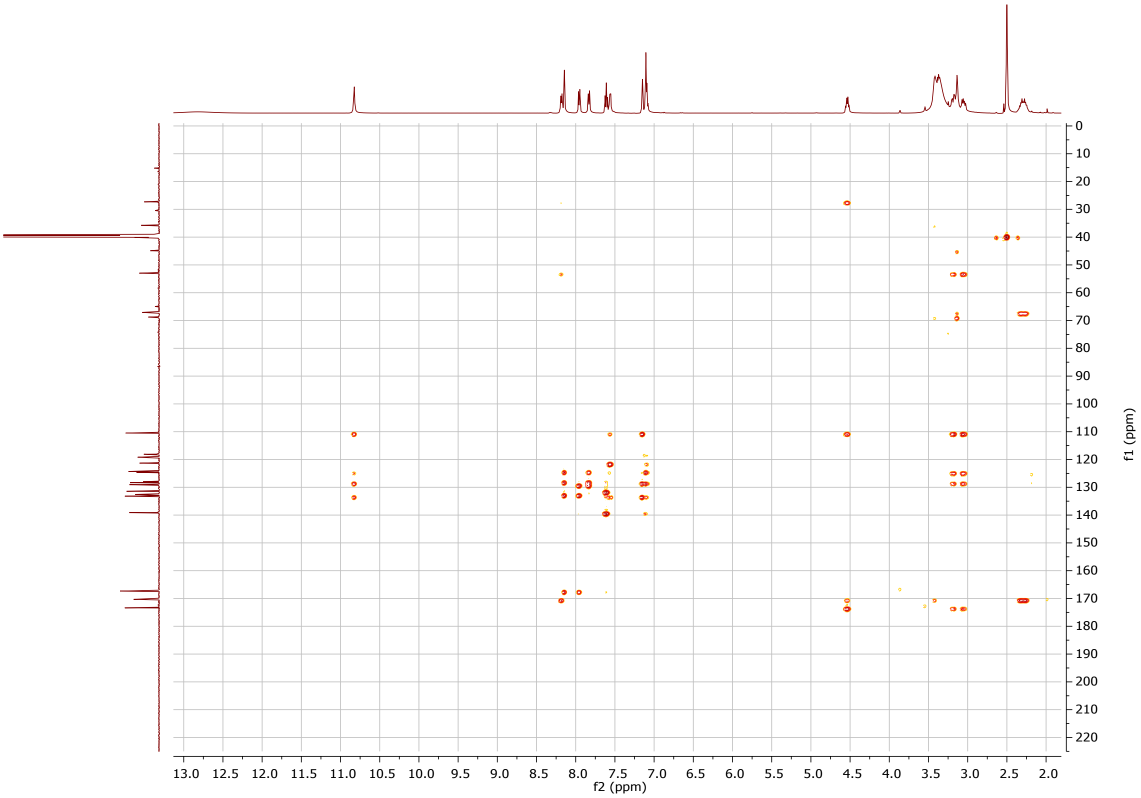

Compound **32**

COSY

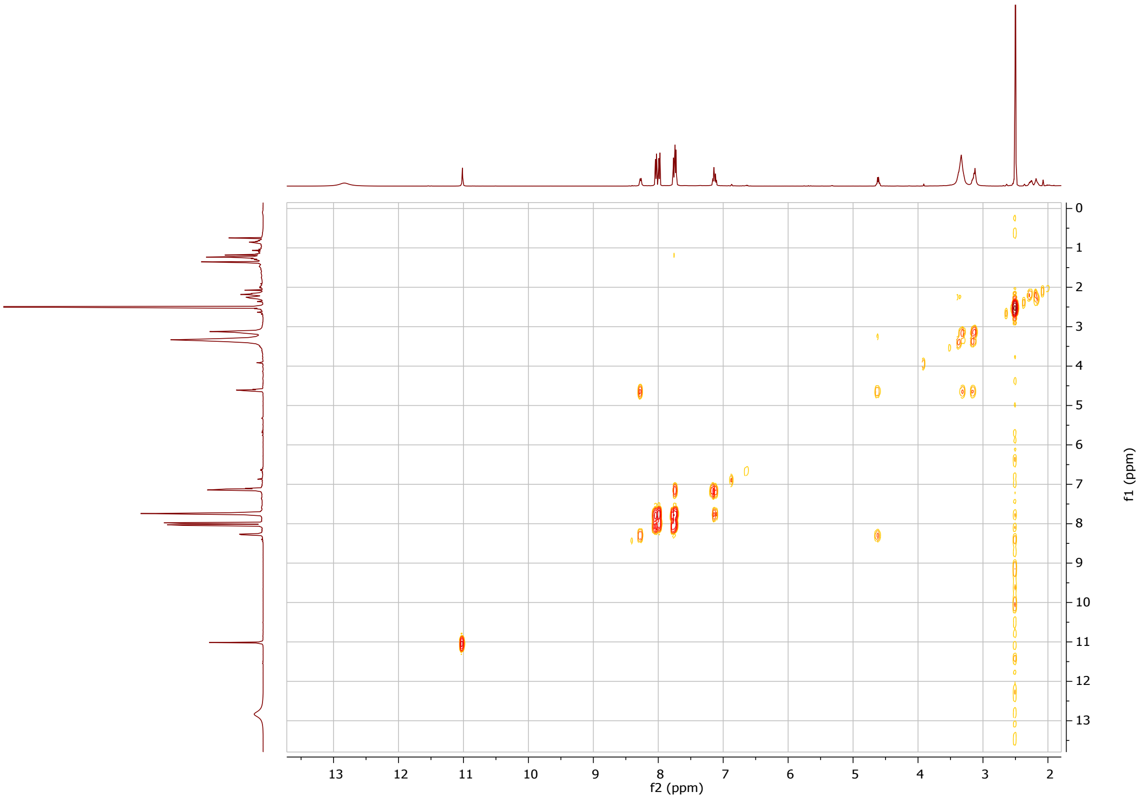

HSQC

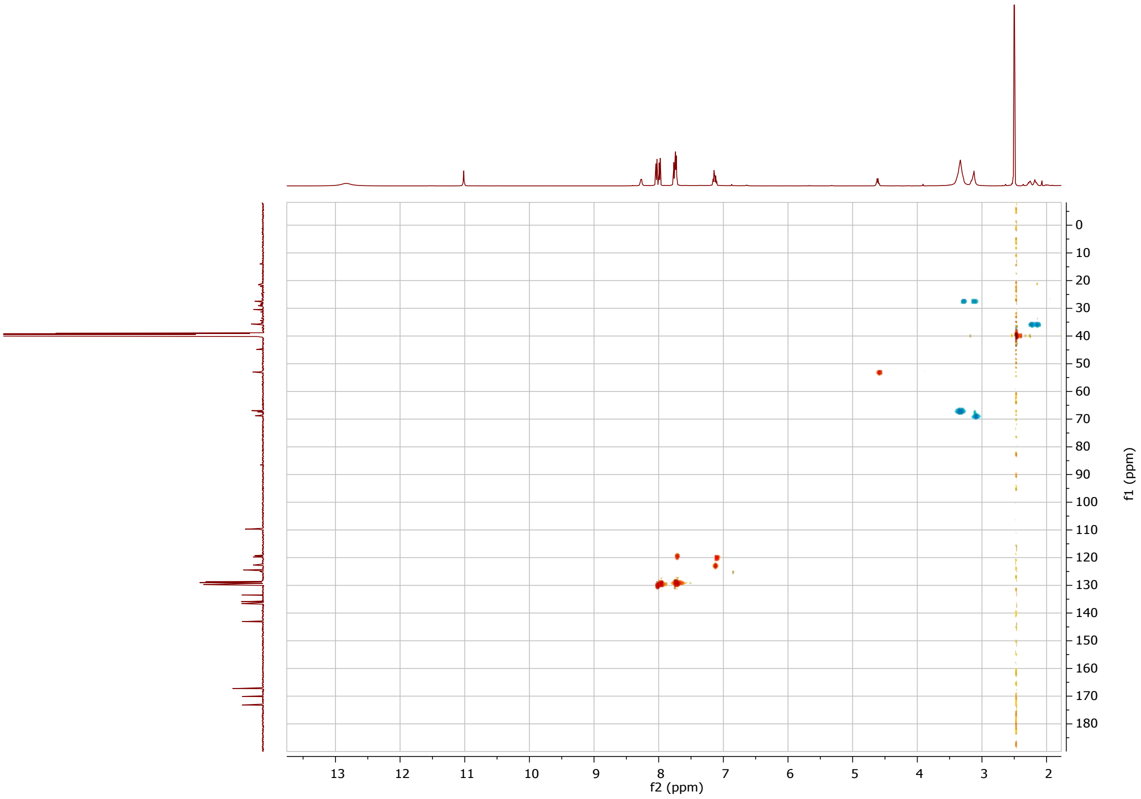

HMBC

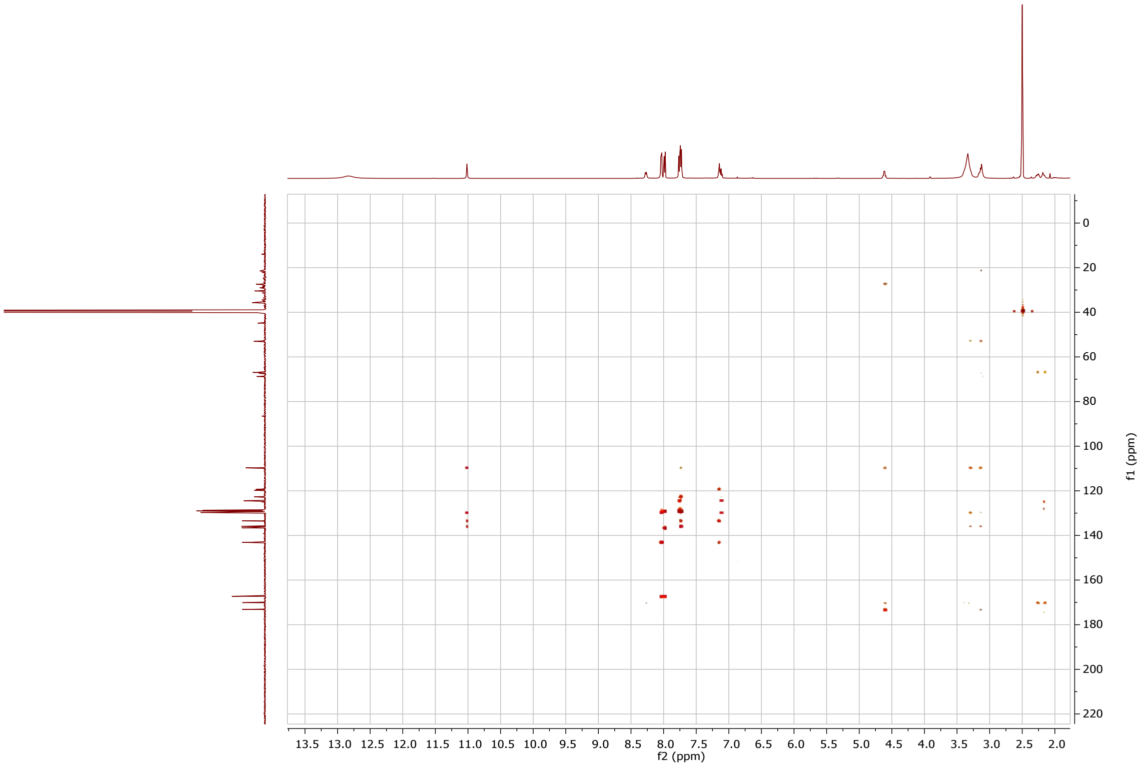

13.5 13.0 12.5 12.0 11.5 11.0 10.5 10.0 9.5 9.0 8.5 8.0 7.5 7.0 6.5 6.0 5.5 5.0 4.5 4.0 3.5 3.0 2.5 2.0
f2 (ppm)
0 20 40 60 80 100 120 140 160 180 200 220
f1 (ppm)

2. ANTIBODY STUDIES

Table S1. *Inhibition of gp120 binding of mAb clone 447-52D by **AL-471** and **33(AL-518)***

Compound	Concentration	Mean fluorescence intensity	Mean fluorescence intensity- mean NC	% inhibition of antibody binding[c]	IC_{50}
NC[a]		715	0		
PC[b]		7366	6651		
**AL-471**	100 µM	5554	4839	27.2	
	20 µM	4829	4114	38.1	>100 µM
	4 µM	5795	5080	23.6	
**33(AL-518)**	20 µM	2248	1533	76.9	
	4 µM	4939	4224	36.5	**6.85 µM**
	0.8 µM	5773	5058	23.9	
	0.16 µM	6239	5524	16.9	
**HHA**	100 µg/ml	7370	6655	0	>100 µg/ml

[a]Negative control. HIV-1 infected MT-4 cells stained only with the secondary antibody (Alexa-fluor-488 goat-anti-mouse mAb). This is background staining or called negative control (NC).
[b]Positive control. HIV-1 infected cells stained with the first (anti-HIV-1 envelope antibody) and then the secondary antibody (Alexa-fluor-488 goat-anti-mouse mAb). This is the positive control (PC).
[c]Percentage of **compound**-mediated inhibition of binding of mAb antibody to the HIV-1 infected MT-4 cells. It was calculated as: mean fluorescence intensity - mean NC/6651 = X, then (1–X) x 100. For example, 4839:6651 = 0.7275, then 1–0.7275 = 0.2725 [x 100 = 27.2% inhibition].

As shown in Table S1, the binding of mAb clone 447-52 to gp120-expressing HIV-1-infected $CD4^+$ T cells was inhibited by 17% up to 77 % in the presence of (sub)micromolar concentrations of **AL-518** (0.16 to 20 µM). On the contrary, **AL-471** had no or only a weak effect on the binding of this antibody to gp120. Finally, the mannose-specific lectin *Hippeastrum hybrid* agglutinin (HHA), used as control, had no effect on virus interaction with the antibody.

Similarly (Table S2), (sub)micromolar concentrations of **AL-518** (0.16 µM to 20 µM) inhibited the binding of mAb clone 9284 by 25% up to 61 % while **AL-471** displayed almost no effect at a concentration up to 100 µM.

Table S2. *Inhibition of gp120 binding of mAb clone 447-52D by **AL-471** and **33(AL-518)***

Compound	Concentration	Mean fluorescence intensity	Mean fluorescence intensity- mean NC	% inhibition of antibody binding[c]	IC_{50}
NC[a]		727	0		
PC[b]		2642	1915		
**AL-471**	100 µM	2423	1696	11.4	
	20 µM	2337	1610	15.9	>100 µM
	4 µM	2563	1836	4.1	
**33(AL-518)**	20 µM	1472	745	61.1	
	4 µM	1657	930	51.4	**3.37 µM**
	0.8 µM	1915	1188	37.9	
	0.16 µM	2161	1434	25.1	

[c]Percentage of **compound**-mediated inhibition of binding of mAb antibody to the HIV-1 infected MT-4 cells. It was calculated as: mean fluorescence intensity - mean NC/1915 = X, then (1–X) x 100. For example, 1696:1915 = 0.8856, then 1–0.8856 = 0.1144 [x 100 = 11.4%].

3. COMPUTER-ASSISTED MOLECULAR MODELING

Figure S1. Mode of engagement of CD4 (cyan) by gp120 (olive green).[1]

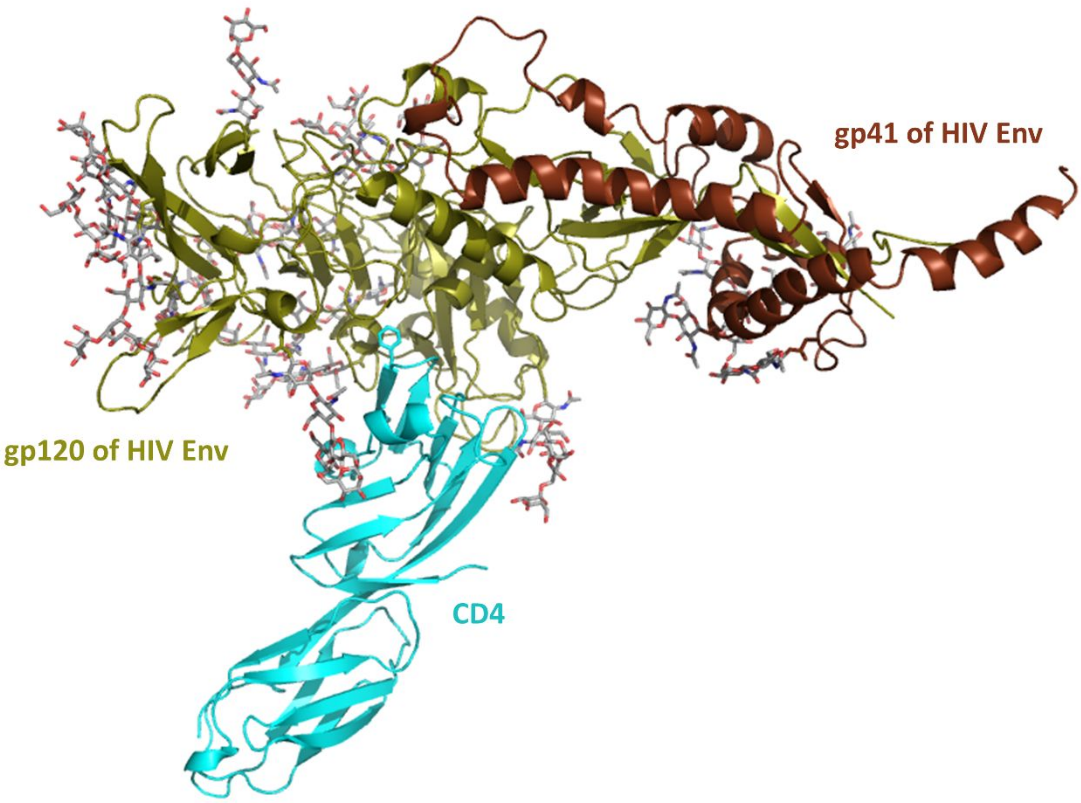

3.1. **Supplementary Movie 1**. Interactions between the isophthaloyl moieties attached to C2 and C7 positions of Trp in **33** (**AL-518**) and four molecules of a model Man$\alpha_{1\text{-}2}$Man disaccharide used as a simplified version of the oligomannose type *N*-glycans present in gp120 and gp41. The snapshots (which have been overlaid using the tetrapodal core for superimposition) correspond to the last 100 ns of the post-equilibration period of the unrestrained molecular dynamics simulations in the presence of explicit water molecules and counterions.

3.2. **Supplementary Movie 2**. Protein surface coverage provided by the highly mobile glycan "cloud" (sticks, with C atoms colored in grey) around gp120 (green cartoon) and gp41 (brown cartoon) in a membrane-embedded HIV-1 Env protein (a trimer of dimers). This glycan shield is likely to be encountered by any approaching ligand, including tetrapodal Trp derivative **33** (**AL-518**). The rotating view shows ten superimposed snapshots of the molecular ensemble taken every 20 ns from the post-equilibration 200-400 ns period of the unrestrained molecular dynamics simulations. Water molecules and counterions on both sides of the membrane have been omitted for clarity.

3.3. **Supplementary Movie 3**. Clustering of solutions and alternative accommodations of **33** (**AL-518**) interacting with the glycans surrounding the V3 loop of gp120 throughout the unrestrained molecular dynamics simulations (200-400 ns post-equilibration period) in the presence of explicit water molecules and counterions.

3.4. **Supplementary Movie 4**. Cartoon representation of an HIV-1 glycosylated gp120:gp41 dimer (olive and brown, respectively) in complex with a two-domain fragment of the human CD4 receptor (cyan). The rotating view shows ten superimposed snapshots of the molecular ensemble taken every 5 ns from the post-equilibration 100-150 ns period of the unrestrained molecular dynamics simulations. Water molecules have been omitted for clarity.

References

(1) Duan, L. W.; Zhang, H.; Zhao, M. T.; Sun, J. X.; Chen, W. L.; Lin, J. P.; Liu, X. Q., A non-canonical binding interface in the crystal structure of HIV-1 gp120 core in complex with CD4. *Sci. Rep.* **2017,** *7*, 46733.
